# New genomic insights into long-chain alkenone biosynthesis by the coccolithophorid marine alga *Gephyrocapsa huxleyi*

**DOI:** 10.1186/s12864-026-12930-5

**Published:** 2026-05-30

**Authors:** Ruth Pérez Gallego, F. A. Bastiaan von Meijenfeldt, Marc A. Besseling, Marcel T. J. van der Meer, Jaap S. Sinninghe Damsté, Laura Villanueva

**Affiliations:** 1 Department of Marine Microbiology and Biogeochemistry, NIOZ Royal Netherlands Institute for Sea Research, PO Box 59, Den Burg, 1790 AB The Netherlands; 2 Department of Earth Sciences, Faculty of Geosciences, Utrecht University, PO Box 80.021, Utrecht, 3508 TA The Netherlands; 3 Department of Biology, Faculty of Sciences, Utrecht University, Utrecht, 3584 CS The Netherlands; 4 Present Address: Hogeschool Leiden, Leiden Centre for Applied Bioscience (LCAB), Leiden, 2333 CR The Netherlands

**Keywords:** Long chain alkenones, *Gephyrocapsahuxleyi*, Polyketide synthase, Desaturase, Lipid biosynthesis, RNAseq, Genomic assembly

## Abstract

**Background:**

*Gephyrocapsa huxleyi* is a coccolithophoric haptophyte widespread across most marine ecosystems that plays an important role in the carbon cycle. *G. huxleyi* is one of the few haptophytes known to produce long-chain alkenones (LCAs), which are lipids composed of C_35_-C_42_ *n*-alkyl chains, with two to four trans-double bonds and a keto group at either the 2nd or 3rd carbon positions, which are widely used as proxies for paleotemperature reconstruction. Despite the biomarker value of LCAs, little is known about their biosynthesis, which severely limits the understanding of the regulation of their production under different conditions, and thus their predictive nature. Differences in gene expression under different conditions may help to unravel the LCA biosynthetic pathway, but require annotated reference genomes. Although a large number of *G. huxleyi* strains inhabiting diverse ecosystems exist, only one such genome (i.e. of strain CCMP1516) is currently available.

**Results:**

To evaluate differences in LCA biosynthesis under changing conditions, we developed a tailored approach to decontaminate and assemble the genomes of two additional non-axenic *G. huxleyi* strains (strains CCMP1742 and CCMP2758) that produce LCAs with two to four double bonds, one of which (CCMP2758) also produces unusually short (C_35_-C_36_) LCAs. We identified a polyketide synthase (PKS) in strain CCMP1516, which we propose as a candidate to carry out LCA formation based on the structure and composition of its modules. Additionally, we identified several CCMP1742 and CCMP2758 PKSs that were differentially expressed between temperatures and across time that closely resemble this PKS and are likely to be involved in LCA formation. Based on differences in gene expression between two different growth temperatures, we also identified a limited number of desaturases which are potentially responsible for the addition of the third and fourth double bonds in the LCAs produced by these strains.

**Conclusions:**

Here, we advance the understanding of biosynthesis of LCAs, important molecules for paleotemperature reconstruction, in the marine haptophyte *G. huxleyi*. By assembling the genomes of two LCA-producing strains, we were able to overcome the limitations of the scarcity of reference genomes of this group, enabling the analysis of gene expression under varying growth conditions and the identification of PKSs and desaturases potentially involved in LCA biosynthesis. These findings constitute a step forward in elucidating the genetic basis for LCA production and regulation in *G. huxleyi*, which not only advances our understanding on how LCAs are formed but also aids in the improvement of LCA-based paleotemperature reconstructions.

**Supplementary Information:**

The online version contains supplementary material available at 10.1186/s12864-026-12930-5.

## Introduction

**Fig. 1:**
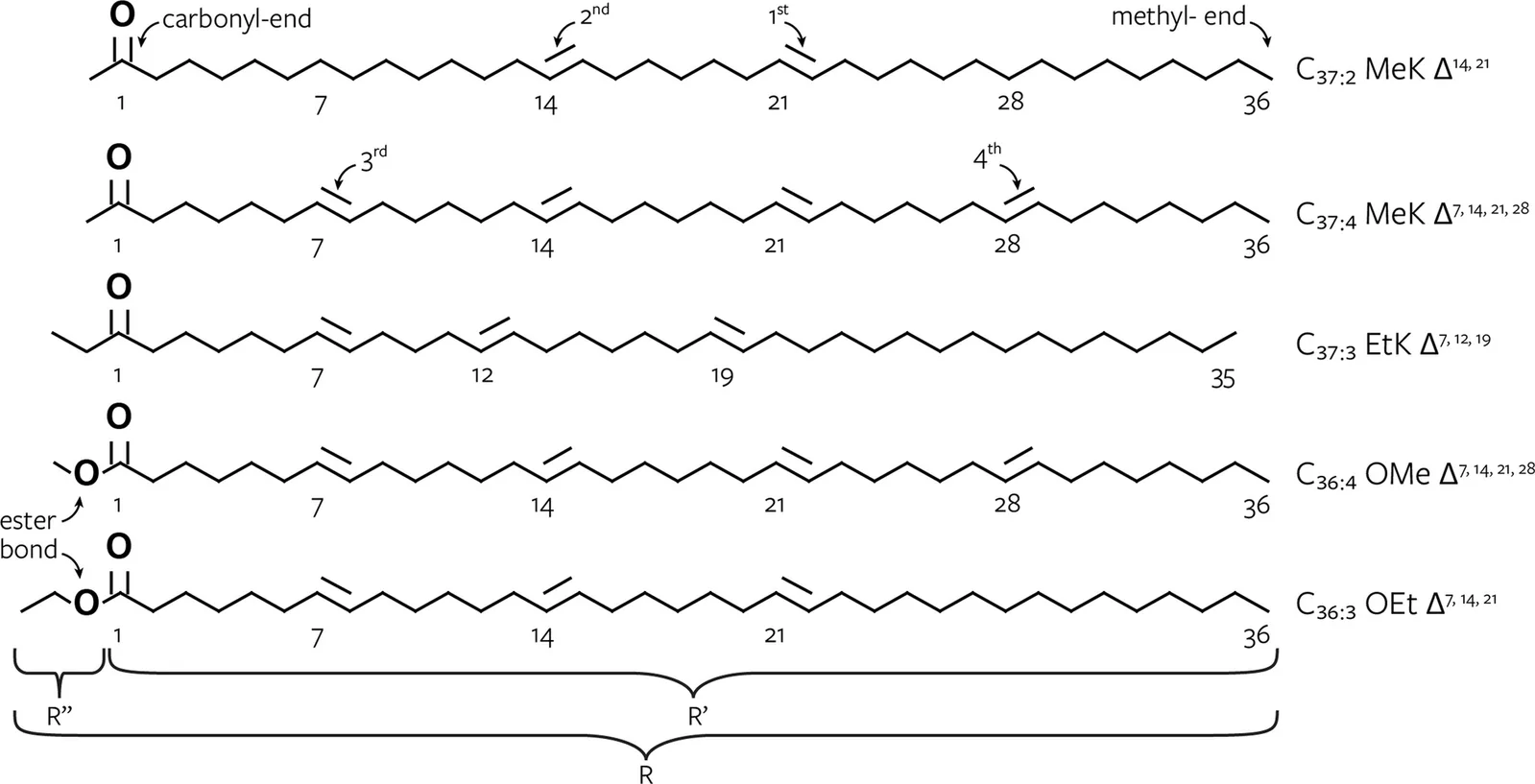
Molecular structure of methyl LCAs C_37:2_ (C_37:2_ MeK) and C_37:4_ (C_37:4_ MeK), ethyl LCA C_37:3_ (C_37:3_ EtK), and of methyl LCE C_36:4_ (C_36:4_ OMe) and ethyl LCE C_36:3_ (C_36:3_ OEt). Although structurally similar, LCAs and LCEs follow different naming conventions. While LCAs are named according to the total number of carbons present in the molecule (depicted as R), LCEs are named according to the number of carbons in the molecule excluding the methyl or ethyl group attached to the ester bond (depicted as R’). For example, a molecule containing a total of 37 carbons and a methyl headgroup will be named C_37_MeK if it’s an LCA and C_36_OMe if it’s an LCE. However, because of the differences in naming between LCAs and LCEs potential changes in chain length (and thus in the biosynthetic pathway) related to temperature or growth stage may not be readily apparent from the compound names. Double bonds are usually separated by five methylene groups (equivalent to seven carbon atoms) from the carbonyl end and between each other, except in C_37:3_ Et where the second and third double bonds are separated only by three methylene groups (5 carbon atoms). Double bond positions are enumerated according to their position in the LCA structure counting from the ketone group and are indicated under the carbon chain. The order in which the double bonds are likely introduced in the molecule is indicated with arrows above the molecule structure. LCE double bond positions are inferred from double bond positions in LCA structures

*Gephyrocapsa huxleyi* (formerly known as *Emiliania huxleyi*) [1] is a coccolithophoric haptophyte widespread across most marine ecosystems that plays an important role in the carbon cycle. *G. huxleyi* is one of the few haptophytes known to produce long-chain alkenones (LCAs) and structurally related long-chain esters (LCEs) [2, 3]. LCAs are lipids composed of C_35_-C_42_ *n*-alkyl chains [4, 5], with at least two and up to four unusual *trans*-double bonds (unsaturations) [5–8] and a keto group at either the 2nd (methyl ketones, MeK) or 3rd (ethyl ketones, EtK) carbon position (Fig. 1, Figure S1) [5]. Long chain esters (LCEs also known as alkenoates) are structurally *c*losely related to LCAs and are defined by the presence of an ester group. LCAs are widely used in paleotemperature reconstructions as part of the sea surface temperature (SST) proxy ${U}_{37}^{k{\prime}}$ (${U}_{37}^{k{\prime}}$ = [C_37:2_]/([C_37:2_] + [C_37:3_]) [9–11]. The ${U}_{37}^{k{\prime}}$ index is based on the observation that the degree of unsaturation of alkenones varies depending on the temperature in which the organisms reside, i.e. decreasing temperatures result in increasing abundances of C_37:3_ relative to C_37:2_ [10]. Initially, the correlation between temperature and unsaturation degree led to the hypothesis that LCAs were involved in membrane fluidity regulation [12]. However, *c*hemical synthesis [7] showed that the double bonds of LCAs possess the unusual *trans* stereochemistry—in contrast to most fatty acids present in membrane lipids—and probably do not act as membrane lipids. Subsequent work indeed showed that LCAs are not located in the cell membrane [13], but instead they accumulate in the cytoplasm in so-called alkenone bodies [14–16] and are likely to function as energy storage [17, 18]. Additionally, physiological factors other than temperature, such as growth phase [19] and salinity [20], and genetic factors such as species composition and their genotypes [21, 22], have also been shown to affect the LCA unsaturation ratios to some degree.

Despite their importance in paleoclimatology and as biomarkers of haptophyte algae, the LCA biosynthetic pathway is still uncertain [22, 23], which severely limits the understanding of LCA production under different conditions, and thus their predictive nature as biomarkers. Currently, two potential pathways have been proposed, both resembling mechanisms found in fatty acid biosynthesis and involving dedicated position-specific desaturases to form each of the four double bonds [22, 23]. Additionally, ^13^C-tracer experiments have shown that sudden temperature changes trigger the conversion of di-unsaturated LCAs into tri-unsaturated LCAs, suggesting the involvement of a desaturase in the formation of this double bond [16], which was later identified as AKD1 in the haptophyte *Tisochrysis lutea* [24]. Paleostratigraphic evidence suggests that this mechanism likely evolved as an adaptation to changing environmental conditions [25]. However, a deeper understanding of the biosynthetic mechanisms involved in the formation and regulation of the chain length and the formation of the *trans*-double bonds under different conditions is still required. Identification of the genes involved in these processes could help to trace their biological producers (within haptophyte algae or beyond) in the environment based on their genomic potential.

To elucidate a biosynthetic gene pathway, one approach is to determine the changes in the expression of genes under physiological conditions leading to differences in the products of interest. This approach is challenging when focusing on eukaryotic organisms as gene expression analysis using transcriptomics requires a well annotated reference genome or transcriptome, and although *G. huxleyi* comprises numerous strains inhabiting diverse ecosystems, only one annotated genome (i.e., that of strain CCMP1516) is currently available [26]. Although the overall number of sequenced algal genomes has increased in recent years, generating complete and well annotated algal genome assemblies is still challenging due to the large genome size, repetitive content, ploidy of algal genomes, and due to the small number of axenic cultures available [26–28]. In addition, another complication is the lack of standardized procedures to assemble, annotate and evaluate algal genomes.

To further constrain the biosynthetic pathway of LCAs, we investigated changes in gene expression and lipid composition over time of three different *G. huxleyi* strains grown at different temperatures, including two strains with previously unavailable genomes. Thus, to be able to constrain changes in gene expression under the different conditions tested, we first generated *G. huxleyi* draft genomes by developing a sequencing, assembly, decontamination and annotation approach tailored to our data. By analysing these genomes using homology searches, phylogenetics, protein structure analysis and differential gene expression analysis focusing on genes potentially involved in LCA formation and desaturation—such as genes annotated as fatty acid synthases (FAS), polyketide synthases (PKSs), and desaturases—we constrained the number of potential gene candidates responsible for LCA biosynthesis. This process comprises two main steps: (1) the biosynthesis of the main LCA chain (referred to hereafter as “formation”) and (2) the addition of the double bonds (“desaturation”). Based on these results, we propose a new putative biosynthetic pathway.

## Results and discussion

**Fig. 2:**
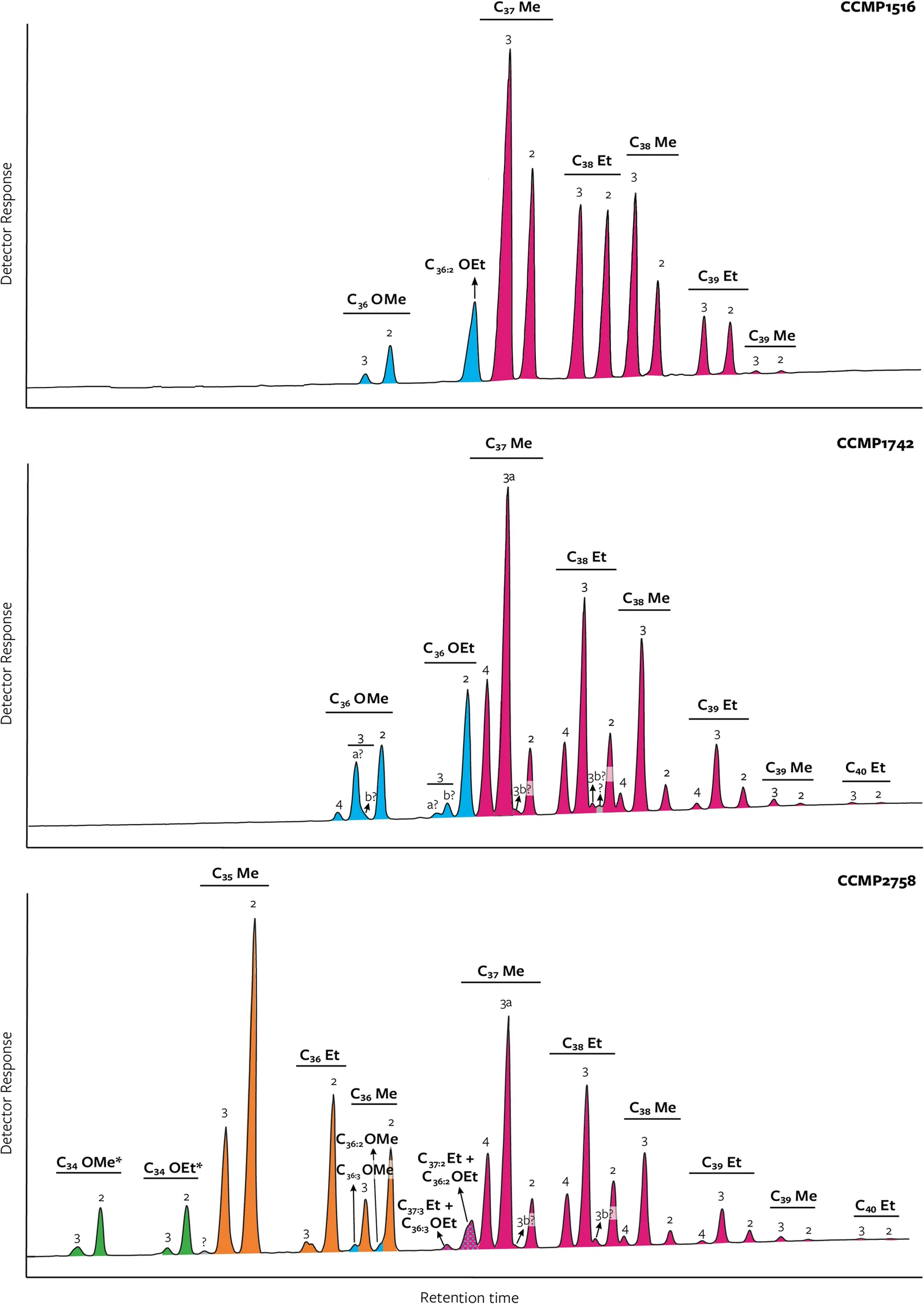
Chromatograms of the ketone fraction obtained from CCMP1516 grown at 15 °C and kept for four days at 3 °C, and CCMP1742 and CCMP2758 grown at 7 °C. The number of double bonds in each compound is indicated above the peak and beneath the compound name. Blue indicates LCEs, dark pink LCAs and patterned print indicates mixtures of LCAs and LCEs. Green and orange indicate LCEs and LCAs, respectively, detected only in strain CCMP2758. Double bond positions in tri-unsaturated compounds are labelled “a” when the third double bond is located closer to the ketone group and “b” when located closer to the methyl terminal group. Asterisks (*) indicate compounds not previously reported in the literature for the respective strain (see Supplementary Information). Files used to generate this chromatogram are available in Data S1. Figure generated with the R package OrgMassSpecR (v 0.5–3) [32]

In this study, we focused on three *G. huxleyi* cultured strains, i.e. CCMP1516, CCMP1742 and CCMP2758, which exhibit different LCA compositions (Table 1). Whilst strain CCMP1516 only synthesizes LCAs with up to three double bonds [29], CCMP1742 and CCMP2758 can also produce tetra-unsaturated LCAs [12, 23] (Table 1 and Fig. 2). CCMP2758 is a natural mutant derived from CCMP1742 that produces shorter chain (C_35_-C_36_) LCAs [30] in addition to the C_37_-C_39_ LCAs that are also produced by CCMP1516 and CCMP1742 [16, 30, 31] (Table 1, Fig. 2 and Data S1). We aimed to constrain the LCA formation pathway by evaluating changes in the production of LCAs over time and across temperatures coupled to the expression of specific genes in these genetically closely related strains exhibiting marked changes in LCA distributions. Since CCMP2758 is closely related to CCMP1742, by evaluating differences in the sequence of the genes tentatively involved in LCA formation, we sought to pinpoint the key genetic elements determining LCA length. Given that the unsaturation degree increases with decreasing temperatures, we also aimed to identify candidate genes encoding the enzymes involved in the addition of the third unsaturation (in all three strains) and fourth unsaturation (in strains CCMP1742 and CCMP2758) to LCAs by comparing changes in expression of genes annotated as, or related to, desaturases under different growth temperatures. To do so, we used two different approaches: cold shock (e.g. abruptly transferring cultures from 20 °C to 10 °C) based on earlier observations that sudden temperature changes trigger the conversion of di-unsaturated LCAs into tri-unsaturated LCAs [16], and temperature adaptation (i.e. change of incubation temperature from 15 °C to 20 °C and 7 °C followed by an acclimatation period of several weeks). Since two of the three tested strains did not have an available genome, we first sequenced them to generate the genomic assemblies needed to carry out our gene expression analysis.

**Table 1:** *Gephyrocapsa huxleyi* strains used in this study

Strain	Collection site	Ocean	Max. number of double bonds	LCA length
CCMP1516	−2.6667°N −82.7167°W	South Pacific	3	C_37_—C_39_
CCMP1742	50.1833°N −144.95°W	North Pacific	4	C_37_—C_40_
CCMP2758				C_35_—C_40_

### Genomic analysis of different LCA-producing strains of *G. huxleyi*

**Fig. 3:**
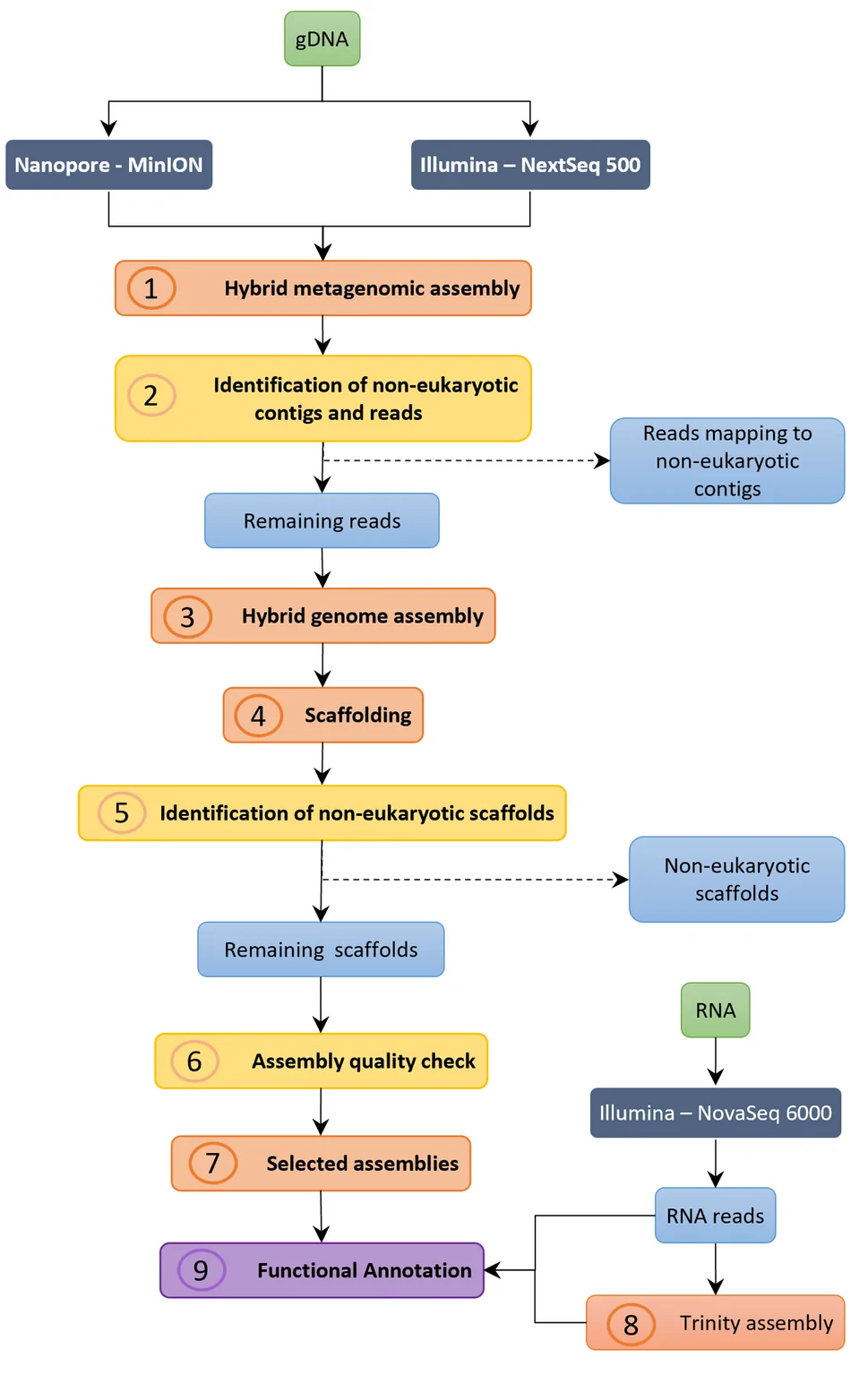
Diagram illustrating the workflow used to assemble the CCMP1742 and CCMP2758 draft genomes, including the steps for identifying and removing contaminant reads. See Figure S3 for details

Previous studies of *Gephyrocapsa* have shown large genomic differences between and within species [1, 26]. Additionally, the only genome assembly available of *G. huxleyi* at the time of the start of this study was that of strain CCMP1516, which only synthesizes C_37_-C_39_ LCAs and does not synthesize tetra-unsaturated alkenones. Therefore, the first essential step was to sequence (via short and long read whole genome sequencing), assemble, and annotate the genomes of strains CCMP1742 and CCMP2758, in order to be able to use these strains in gene expression (transcriptomics) experiments. A complication in this effort is that both cultures are non-axenic, and thus also contain bacterial and/or archaeal DNA. After testing and evaluating several long-read, short-read and hybrid genome and metagenomic assemblers (Supplementary Information, Figure S2, Tables S1, S2 and Data S2), we designed an approach to remove contamination, whilst preserving as much information of the *G. huxleyi* genomes as possible. Our approach involved: (1) assembling long and short reads as a metagenome using the metaSPAdes assembler [33], (2) filtering out reads belonging to likely contaminants, (3) assembling our genomes into contigs using hybridSPAdes and biosyntheticSPAdes [34, 35], (4) assembling contigs into scaffolds using OPERA-LG [36], and (5) removing scaffolds belonging to likely contaminants (Fig. 3, Supplementary Information, Figures S3–S5, Tables S3, S4, and Data S3). The resulting genomes, although fragmented, showed high completeness based on BUSCO (Benchmarking Universal Single-Copy Orthologs) [37] results: ~68% of eukaryotic BUSCOs were present in both strains (Table S5), only slightly lower than the CCMP1516 reference genome, which contains 73% of the eukaryotic BUSCOs (Supplementary Information). We compared the results of the SPAdes assembler when using long and short reads in default mode (hybridSPAdes) [34] and in bio mode (biosyntheticSPAdes) [35], which generates an additional file (“gene_clusters” file) containing predicted biosynthetic gene clusters (BGCs), that are potentially relevant for lipid biosynthesis (Supplementary Information, Figure S6, Tables S6– S8). Assemblies for the genomes of both strains obtained using hybridSPAdes and biosyntheticSPAdes were highly similar (Supplementary Information, Tables S4 and S5). Given that some genes of interest might be located within a BGC (e.g. Fatty acid synthases (FASs) and polyketide synthases (PKSs), see below), all downstream analyses exclusively used assemblies generated with biosyntheticSPAdes. Assemblies were annotated with Funannotate, an annotation pipeline that integrates multiple software tools to predict and functionally annotate genes (Data S4) [38]. The resulting de novo assemblies were 130–133 Mega base pairs (Mbp) in size, approximately 17–19% smaller than the CCMP1516 reference genome (160 Mbp); yet Funannotate predicted a slightly larger number of genes (2–8%) and proteins (8–14%), than those predicted by the JGI annotation pipeline for the CCMP1516 reference genome (Table 2, Supplementary Information, Table S4 and S5).

**Table 2:** Genome assemblies and annotations used in this study

Strain	Assembler	Assembly size (Mbp)	Annotation pipeline	Number of genes	Number of proteins	LCA desaturases	LCA biosynthesis
CCMP1516	Arachne	167.7	JGI	38,554	38,554	22	53
			Funannotate	32,526	37,317	52	178
CCMP1742	biosyntheticSPAdes	130.3		39,344	41,570	28	209
CCMP2758		133.4		41,557	43,849	37	248

To assess the impact of using different functional annotation pipelines on our results, we re-annotated the CCMP1516 genome using Funannotate and compared it to the reference genome annotation (hereafter “reference annotation”) using two different methods; the “compare” command within the Funannotate pipeline and OrthoFinder within the OrthoVenn3 platform [39, 40]. ‘Funannotate compare’ results showed that the annotation generated using Funannotate contains 6,018 genes and 1,237 proteins less than the reference annotation (Table S5) [26]. Additionally, only 67–73% of these proteins are identified as orthologues to the reference annotation by OrthoFinder, thus highlighting significant discrepancies between pipelines. These results underscore the need for caution when comparing annotations generated using different annotation pipelines, since a gene detected by one pipeline might be missed by another, thus, apparent gene 'absences' may simply reflect differences in gene prediction rather than true biological differences.

### Identification of candidate genes involved in the initial step of LCA formation

**Fig. 4:**
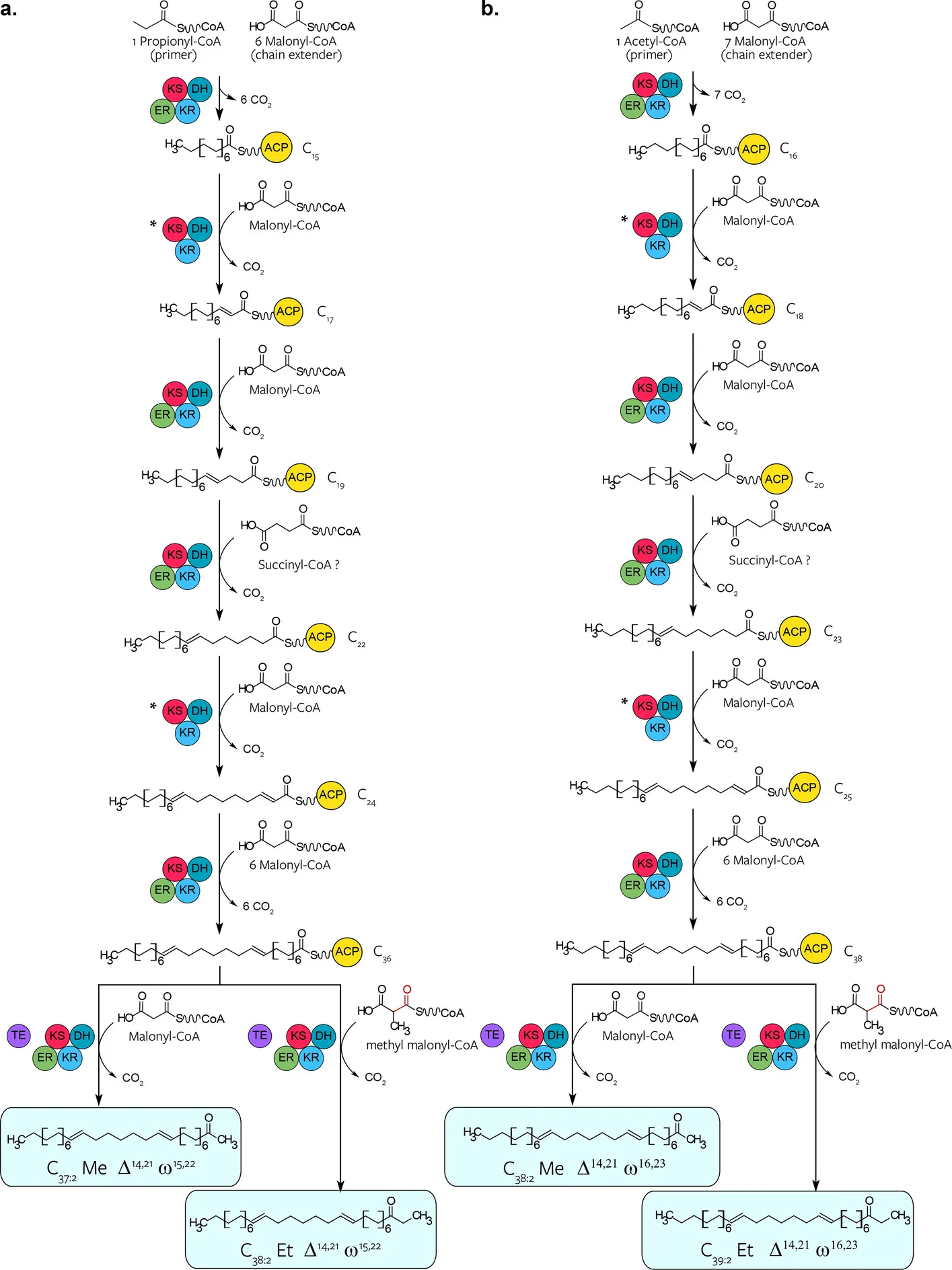
Proposed pathway for the synthesis of **a**) C_37:2_Me ∆^14,21^ and C_38:2_ Et ∆^14,21^ LCAs using Propionyl-CoA as starter unit and **b**) C_38:2_Me ∆^14,21^ and C_39:2_ Et ∆^14,21^ LCAs using Acetyl-CoA as starter unit. Modules lacking an ER domain are indicated with a star (*). See Results and Discussion for further details

It has been proposed that LCAs are synthesized as fully saturated chains and that subsequently double bonds are introduced by the action of at least four position-specific desaturases [22, 23]. However, the (almost) complete absence of saturated and mono-unsaturated LCAs in combination with the predominant abundance of LCAs with at least two double bonds (Table S9) [5–7, 22, 30, 41–46] suggests an alternative possibility, i.e. that LCAs are synthesized directly in their di-unsaturated form. Hence, we propose an LCA biosynthetic pathway where LCAs are synthesized directly in their di-unsaturated form, and where the insertion of the third and fourth double bond is carried out later in the biosynthetic process by two position-specific desaturases (Fig. 4).

FASs and PKSs are good candidates for carrying out LCA biosynthesis. They operate in similar ways; both types of enzymes possess several subunits, an acyltransferase (AT) subunit—which can be encoded by genes located in close proximity to the other PKS and FAS genes or located elsewhere in the genome—that catalyzes the attachment of a substrate to the acyl carrier protein (ACP), and a ketosynthase (KS) that catalyzes the condensation of two substrates, while the ketoreductase (KR), dehydratase (DH) and enoylreductase (ER) subunits catalyze the stepwise processing of the polyketide intermediate to reduce the β-keto group (KR), form a double bond (DH)—which usually has the *trans* stereochemistry [47]—and reduce the double bond to a single bond (ER) generating a fully saturated chain. While KR, DH and ER are always present in FASs—thus resulting in a fully saturated product—these subunits are not always present in PKSs. Additionally, while FASs usually possess only one of each of these subunits, some PKSs known as “assembly line PKSs” can contain multiple copies of each subunit arranged in so-called modules [48]. The modular architecture of these PKSs can give an approximate indication of the chemical structure of the final compound [49, 50]. These assembly line PKSs possess the structural, mechanistic and functional characteristics that are also required for LCA biosynthesis, namely the use of iterative Claisen condensations to build long *n*-alkyl chains [49] and the existence of a modular architecture that can be tailored to produce compounds of a certain length and to introduce special features including *trans*-double bonds and keto groups [48, 49], such as the ones found in LCAs. The role of PKSs in the production of *n*-alkyl compounds with variable structure has already been shown for other compounds such as heterocyte glycolipids in heterocytous cyanobacteria [51, 52]. If LCAs are indeed directly synthesized in their di-unsaturated form, we propose here that PKSs could carry out LCA biosynthesis via a chain elongation process in which the reduction step is omitted twice, resulting in the presence of two *trans*-double bonds. However, if LCAs are synthesized as initially proposed [22, 23], FASs are also likely candidates to carry out LCA formation. We, therefore, searched the genomes for both PKSs and FASs.

We identified different numbers of genes encoding proteins related to FASs and PKSs based on functional annotation: 209 in CCMP1742, 248 in CCMP2758, and 178 in CCMP1516 when annotated with Funannotate, and 53 in the CCMP1516 reference annotation (Table 2). Upon manual inspection of the CCMP1516 reference annotation, we identified a protein, PKS7, which—based on its domain architecture—is a promising candidate for catalyzing the “one-step” formation of LCAs. PKS7 (XP_005762094 encoded by EMIHUDRAFT_632072) is a very large type I modular assembly-line PKS composed of 21,165 amino acids (aa) arranged in 13 modules, most of which contain a nearly complete set of domains—containing KS, KR, DH, ER, and ACP, but lacking AT—that would generate an *n*-alkyl chain with no double bonds, whilst two lack the ER domain responsible for the removal of the (usually *trans-*) double bond—like the ones found in LCAs—during chain elongation (Figure S7) [47]. As all modules lack an AT domain, the necessary AT activity is therefore likely provided in *trans* by a separate acyltransferase subunit encoded by a distinct gene. This biosynthesis mechanism would thus explain the unusual *trans*-configuration of LCAs, at least for the first two double bonds [47].

In addition to PKS7, the CCMP1516 reference annotation contains another large assembly-line PKS, PKS9 (XP_005764102 encoded by EMIHUDRAFT_632088) which is composed of 20,899 aa and—based on bi-directional best BLAST hit—is the most closely related protein to PKS7 in the genome. However, its domain architecture does not seem compatible with our proposed LCA biosynthetic mechanism, as some of its modules lack KR domains, suggesting that the resulting molecule would contain keto groups throughout the *n-*alkyl chain.

We thus propose that PKS7 could catalyze the synthesis of C_37:2_ and C_38:2_ MeK by using either Propionyl-CoA or Acetyl-CoA as starter units, followed by successive elongation rounds with Malonyl-CoA units—provided by an AT located elsewhere in the genome—and carried out by complete elongation modules, interspersed with two ER-deficient modules—that would give rise to the ∆^14,21^ unsaturations (Fig. 4, Supplementary Information and Figures S7–S9). C_38:2_ and C_39:2_ EtK ∆^14,21^ could be synthesized following the same mechanism—and perhaps even by the same protein—but using a Methylmalonyl-CoA extender unit during the last elongation step. Additionally, fewer elongation rounds could also explain the biosynthesis of C_35_–C_36_ LCAs, eliminating the need to invoke chain shortening steps, as previous LCA biosynthetic pathway hypotheses have suggested [22, 23]. LCEs could be synthesized via a similar pathway, as it was also suggested in previous studies [30], and perhaps even by the same enzyme. While the general composition of the final molecule can be anticipated [53] it is not yet possible to accurately determine its exact final structure based solely on PKS architecture [49, 50]. Thus, the reactions and final products of PKS7 mentioned here are tentative and would require further experimental validation to confirm or refute this hypothesis.

Given our hypothesis that PKS7 is involved in LCA formation in CCMP1516, we performed BlastP searches using PKS7 as query in the genomes of strains CCMP1742 and CCMP2758. This search yielded 92 hits in CCMP1742 and 94 in CCMP2758, which were then added to our list of PKS and FAS-related candidate genes potentially involved in LCA formation if they were not in the list yet. However, the total length of most of these protein hits was small in comparison with the very large PKS7 protein (21,165 aa). Only two hits for CCMP1742 corresponded to proteins larger than 3,000 aa (Table S10), whilst for CCMP2758 five hits were proteins that are at least half as long as PKS7 (~10,000 aa), of which four correspond to two sets of identical proteins found within the BGC “gene_cluster” file generated by biosyntheticSPAdes (i.e. BGC-CCMP2758_FUN_000059-T1 = BGC-CCMP2758_FUN_000060-T1 of 10,886 aa each, and BGC-CCMP2758_FUN_000061-T1 = BGC-CCMP2758_FUN_000062-T1, of 10,904 aa each, Table S8 and S10). Closer examination of these four proteins revealed that they originated from a single cluster, and they thus represent different possible arrangements of the BGC. For example, the proteins that were identical also shared the same nucleotide sequence and only differed in the positions of their protein domains as predicted by biosyntheticSPAdes (Table S11). The four proteins may thus represent a single gene and might be an artifact caused by difficulties encountered by the assembly method when trying to reconstruct long repetitive sequences [35]—such as those found in PKSs—where different assembly paths resulted in the same nucleotide sequence (albeit with different protein domain predictions), and in two slightly divergent protein sequences (i.e. CCMP2758_FUN_000059-T1 and BGC-CCMP2758_FUN_000061-T1). Alternatively, the four predicted proteins may represent two nearly identical genes that are difficult to assemble and were collapsed into a single BGC by biosyntheticSPAdes. Additionally, to evaluate possible differences caused by different annotation pipelines, we also performed this search in the CCMP1516 genome annotated with Funannotate. This yielded 21 hits, none of which were as large as the PKS7 identified in the CCMP1516 reference annotation. The largest hit corresponded to a protein ~4,000 aa shorter (CCMP1516_FUN_006723 of 17,001 aa), and the second largest was approximately half the length of PKS7 (CCMP1516_FUN_008488 of 10,287 aa) (Table S10).

Thus, although no single ~21,100 aa homolog of PKS7 was detected in CCMP1742 nor CCMP2758, it was also not detected in CCMP1516 when annotated with Funannotate, despite using the same nucleotide sequence as the reference annotation. Therefore, we suggest that some of the smaller homologs identified here might be part of a PKS7-like gene that has not been fully assembled or annotated —likely due to the very large size of the gene and to its repetitive structure—resulting in only partial structures or individual subunits being identified.

### Identification of candidate desaturases

Analysis of the chemical structures of LCAs previously reported indicates that the position of the double bonds in relation to the carbonyl moiety is identical for most LCAs (Fig. 1, Figure S1 and Table S9) [5–7, 22, 30, 41–46, 54]. LCAs usually contain two to four double bonds separated by five methylene units; i.e. the C_37:4_ MeK and C_38:4_ EtK contain double bonds at ∆^7,14,21,28^ (Fig. 1) [54]. In our LCA biosynthesis hypothesis, the double bonds at positions ∆^14,21^ are already determined during LCA formation by the PKS protein, and the two additional double bonds at ∆^7^ and ∆^28^ will have to be introduced by desaturases. However, in some strains double bonds are separated by three methylene groups, for example, in C_35:2_ and C_36:2_ LCAs from *G. huxleyi* CCMP2758 the second unsaturation occurs closer to the carbonyl end at positions ∆^12,19^ [22, 30], while the position of the third unsaturation remains unchanged at ∆^7^ [23]. Since the distance between the keto group and the third unsaturation remains constant amongst LCA structures (i.e. ∆^7^) it is likely that the desaturase responsible for the addition of this double bond is a desaturase acting at a fixed distance from the carbonyl group (∆-desaturase) [23]. Additionally, the position of the fourth unsaturation—found almost exclusively at ∆^28^, closest to the methyl end of the molecule and separated by 5 methylene units from the ∆^21^ double bond (Fig. 1, Supplementary Information and Table S9)— and the lack of tetraunsaturated C_35_ and C_36_ LCAs in *G. huxleyi* CCMP2758 [22] suggests that the enzyme responsible is likely a ∆^28^ desaturase requiring the three existing double bonds to be at particular positions (∆^7,14,21^) to function.

We identified 28, 37, 52 and 22 different genes encoding potential desaturases in the genomes of strains CCMP1742, CCMP2758 and CCMP1516 annotated with Funannotate, and the CCMP1516 reference annotation, respectively (Table 2). For the CCMP1516 genome assembly annotated with Funannotate this is more than twice the number of desaturase-encoding genes found in the CCMP1516 reference annotation. This underscores again the challenges of comparing genomes annotated with different annotation pipelines.

In the haptophyte *Tisochrysis lutea,* alkenone desaturase 1 (AKD1) has been identified as the desaturase responsible for the conversion of C_37:2_ to C_37:3_ LCAs [24]. To identify its orthologue(s) in *G. huxleyi* we performed a BlastP homology search to the NCBI nr database (accessed on 10th August 2023) limiting the search to “*Emiliania huxleyi* CCMP1516 (taxid:280,463)” [26, 55, 56]. The homology search identified five putative homologs meeting our significance thresholds of the alignment metrics (≥ 50% query coverage, ≥ 30% aa identity and bitscore > 150), which included EMIHUDRAFT_454486 and the four desaturases previously also identified in [24] as likely candidates for the formation of the third double bond in CCMP1516. Of these, EMIHUDRAFT_196284 and EMIHUDRAFT_454486 displayed the strongest alignment score to AKD1, achieving the highest values across all three alignment metrics (Supplementary Information, and Table S12 and S13), thus suggesting that one of these two proteins may correspond to the desaturase responsible for the addition of the third double bond in LCAs. In the upcoming sections we will describe our approach to confirm this hypothesis and propose candidate desaturases responsible for the addition of the fourth double bond in LCAs.

### Evaluating candidate genes through culturing: monitoring LCA profiles and gene expression

The aim of our culture experiments was to determine which changes in growth conditions would be most appropriate to induce significant changes in LCA formation and subsequent unsaturation between two contrasting growth conditions [16]. The assumption is that under contrasting growth conditions the genes involved in LCA formation and subsequent further desaturation are differentially expressed, further aiding or confirming the genes responsible for these biosynthetic steps.

#### Effect of cold shock on LCA formation and subsequent desaturation in strain CCMP1516

**Fig. 5:**
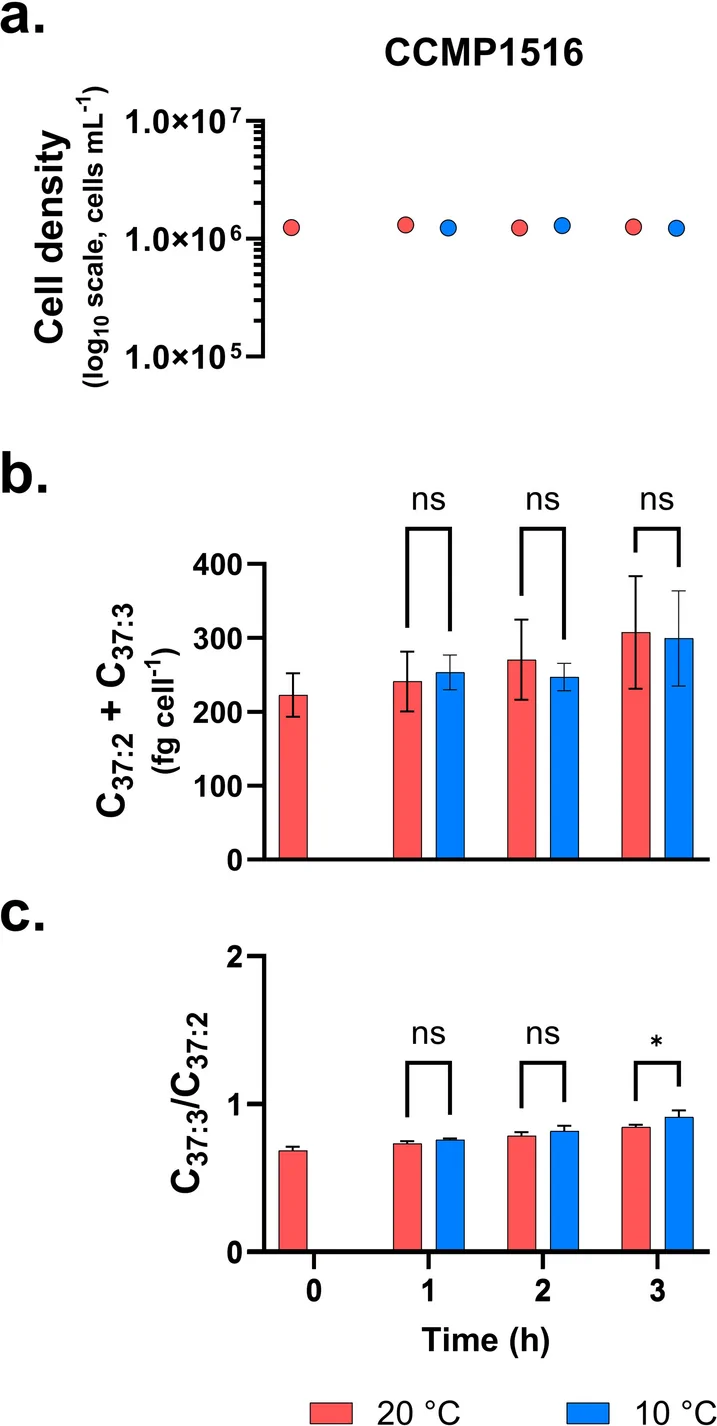
Cell abundances (raw values displayed on a log_10_-scaled axis) (**a**), C_37:2_ + C_37:3_ abundance in femtograms (fg) per cell (**b**) and ratio of C_37:3_/_C37:2_ alkenones (**c**) during cold-shock experiment carried out with *G. huxleyi *strain CCMP1516 grown at 20 °C and transferred to 10 °C. X axis indicates time in hours after the cold shock. Capped bars indicate standard deviations of measurements of triplicate cultures. Cell densities were measured in triplicate cultures; however, standard deviation bars are so small that they are not visible on the graph. Significant differences in total LCA abundances were tested using multiple unpaired t-tests with Holm-Šídák method, and the C_37:3_/C_37:2_ alkenones ratio were tested using a 2-way ANOVA and the Šídák multiple comparisons test. ns, non significant; *, *p*-value ≤ 0.05

The first so-called cold shock experiment was performed with *G. huxleyi* CCMP1516, a strain isolated from the temperate south Pacific Ocean, whose LCA profile includes C_37:2_ and C_37:3_, but not C_37:4_ [3, 5] (Table 1). We initially opted for cold shock experiments because a previous ^13^C-labelling study showed that sudden temperature changes trigger the conversion of di-unsaturated LCAs into tri-unsaturated LCAs in *G. huxleyi* [16]. Cultures were grown at 20 °C, then half of the culture flasks were suddenly transferred to a 10 °C culture room for 3 h (Supplementary Information). Three independent flasks kept at 20 °C were immediately harvested to serve as reference point (t0), then cultures were harvested hourly in triplicate at both temperatures to perform lipid and differential gene expression analysis (Fig. 5). Cell concentrations remained stable throughout the experiment for both the control (20 °C) and cold-shocked (10 °C) cultures (Fig. 5a, Data S5 and Data S6). The C_37:2_ + C_37:3_ LCA concentration per cell, which in this and subsequent cold-shock experiments were used as an indicator of overall LCA formation, did not change throughout this experiment in neither condition (Fig. 5b, Figure S10, Data S1 and Data S6). Thus, LCAs are likely not being synthesized, and no differential expression of the genes involved in C_37:2_ LCA formation can be expected. However, already after 3 h, the C_37:3_/C_37:2_ LCA ratio of the cold-shocked cultures was significantly higher (*p*_*adj*_ ≤ 0.05, detailed statistical tests for all figures are provided in Data S6) than in the control cultures at 20 °C (Fig. 5c). Thus, since the observed change in the C_37:3_/C_37:2_ LCA ratio was most likely caused by an enzymatic conversion of the C_37:2_ into the C_37:3_ LCA as previously shown in *T. lutea* [16], we expect to see increased expression of the desaturase responsible for the addition of the third double bond to LCAs in these conditions.

From the five putative desaturase homologs of AKD1 identified here and in [24], EMIHUDRAFT_454486 was the only one that was significantly overexpressed in CCMP1516 when transferred from 20 °C to 10 °C (log2-fold change = 2.4, *p*_*adj*_ = 2.4 e-27) (Supplementary Information, Figures S11–S14 and Table S14). Thus, based on its homology to ADK1 and overexpression in cold temperatures, EMIHUDRAFT_454486 is likely the desaturase that carries out the third unsaturation (at position ∆^7^) in *G. huxleyi* CCMP1516. This hypothesis was recently confirmed experimentally in another study where a significant increase in the C_37:3_/C_37:2_ ratio was observed when EMIHUDRAFT_454486 was heterologously expressed in *T. lutea* [57].

#### Effect of cold shock on LCA formation and subsequent desaturation in strains CCMP1742 and CCMP2758

**Fig. 6:**
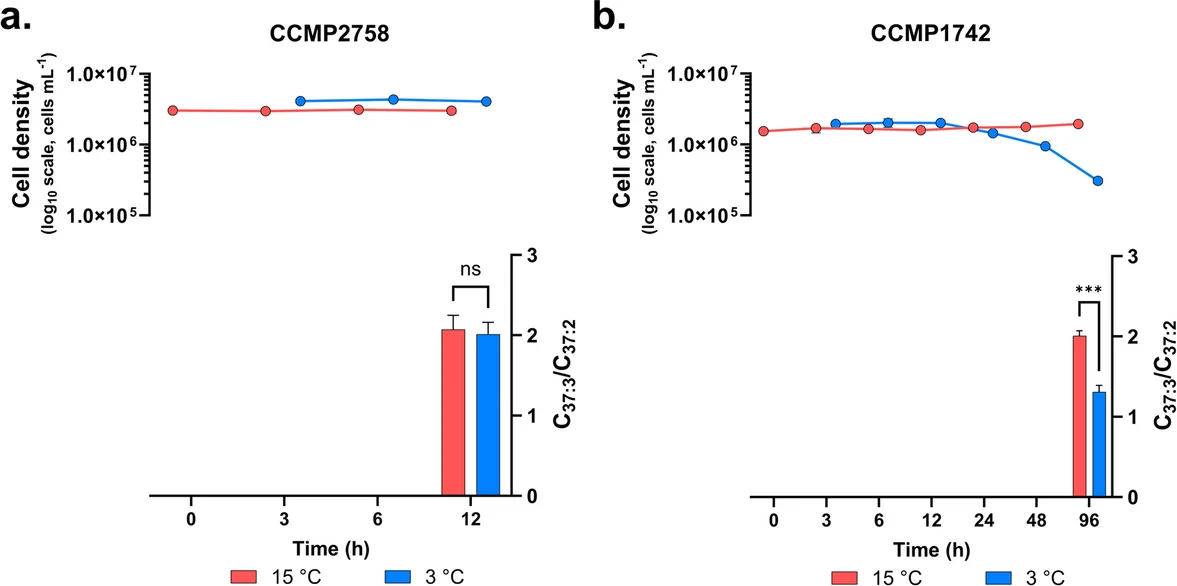
Cell abundances (raw values displayed on a log_10_-scaled axis) (circles, top) and ratio of C_37:3_/C_37:2_ alkenones (bars, bottom) during cold-shock experiments carried out in *G. huxleyi *strains. **a)** CCMP2758 and **b**) CCMP1742 grown at 15 °C and transferred to 3 °C. X axis indicates time in hours after the cold shock. Capped bars indicate standard deviations of measurements of triplicate cultures. Significant differences in the C_37:3_/C_37:2_ alkenones ratios were tested using a paired t-test. ns, non significant, *p*-value > 0.05; *, *p*-value ≤ 0.05; **, *p*-value ≤ 0.01; ***, *p*-value ≤ 0.001; ****, *p*-value ≤ 0.0001

To elucidate the gene(s) responsible for the insertion of the fourth double bond in LCAs, we also carried out cold shock experiments using *G. huxleyi* strains isolated from higher latitudes (Table 1) and known to synthesize tetra-unsaturated LCAs when grown at temperatures below 15 °C, i.e., strains CCMP2758 and CCMP1742 [12, 30]. CCMP2758 cultures were grown in triplicate at 15 °C, then half of the cultures were transferred to a 3 °C culture room and monitored for 12 h (Fig. 6). Unexpectedly, no significant changes (*p*_*adj*_ > 0.05, Data S6) in the cellular C_37:4_ LCA concentration, nor in the C_37:3_/C_37:2_ ratio were observed in the cultures transferred to 3 °C after 12 h (Fig. 6a). Thus, in the next experiment, this time carried out with strain CCMP1742 (a strain very closely related to CCMP2758), we extended the cold shock exposure time to 96 h. After 96 h at 3 °C cellular concentrations of di-, tri- and tetra-unsaturated C_37_ LCAs increased (Figure S15 and Data S6). However, the C_37:3_/C_37:2_ ratio was significantly, and unexpectedly, lower for cold shocked cultures than for cultures kept at 15 °C (*p*_*adj*_ ≤ 0.001, Data S6), indicating that at 15 °C more C_37:3_ in relation to C_37:2_ was produced than at 3 °C (Fig. 6b). This coincided with a decrease in cell abundances at 3 °C, suggesting that the per-cell alkenone increase resulted from population decline caused by cell death. As cells died, the cell abundances detected by flow cytometry declined, while cellular debris containing alkenones was collected during the culture filtration step for alkenone quantification. Due to cell death and the accompanying cell lysis (as indicated by the decrease in cell counts), RNA integrity may have been compromised, rendering this experiment unfortunately unsuitable for gene expression analysis. Additionally, in both experiments after only 3 h we observed an increase in cell abundances in cultures transferred to 3 °C. Given that *G. huxleyi* divides faster at warm temperatures (12–24 h doubling time at 18–20 °C), than at cold temperatures (32–96 h doubling time at 6–9 °C) [21], and that this increase occurred in a very short time span and only in the flasks kept at 3 °C, it seems unlikely to have resulted from sudden cell division. While the exact reason of this increase is unknown, it might have been caused by changes in the cell culture affecting the cell counting method, such as increased cell sedimentation (as observed in later experiments; see Supplementary Materials and Methods) or cell lysis, either of which might have led to an overestimation of cell numbers. These results suggest that while cold shock is an effective approach to study the increase of unsaturation degree of LCAs in strains from the temperate ocean such as CCMP1516, drastic temperature changes do not seem to have the same effect on strains originating from colder environments, which might be caused by a slower response to sudden environmental changes.

#### Impact of temperature acclimation on LCA formation and subsequent desaturation in CCMP1742 and CCMP2758

**Fig. 7:**
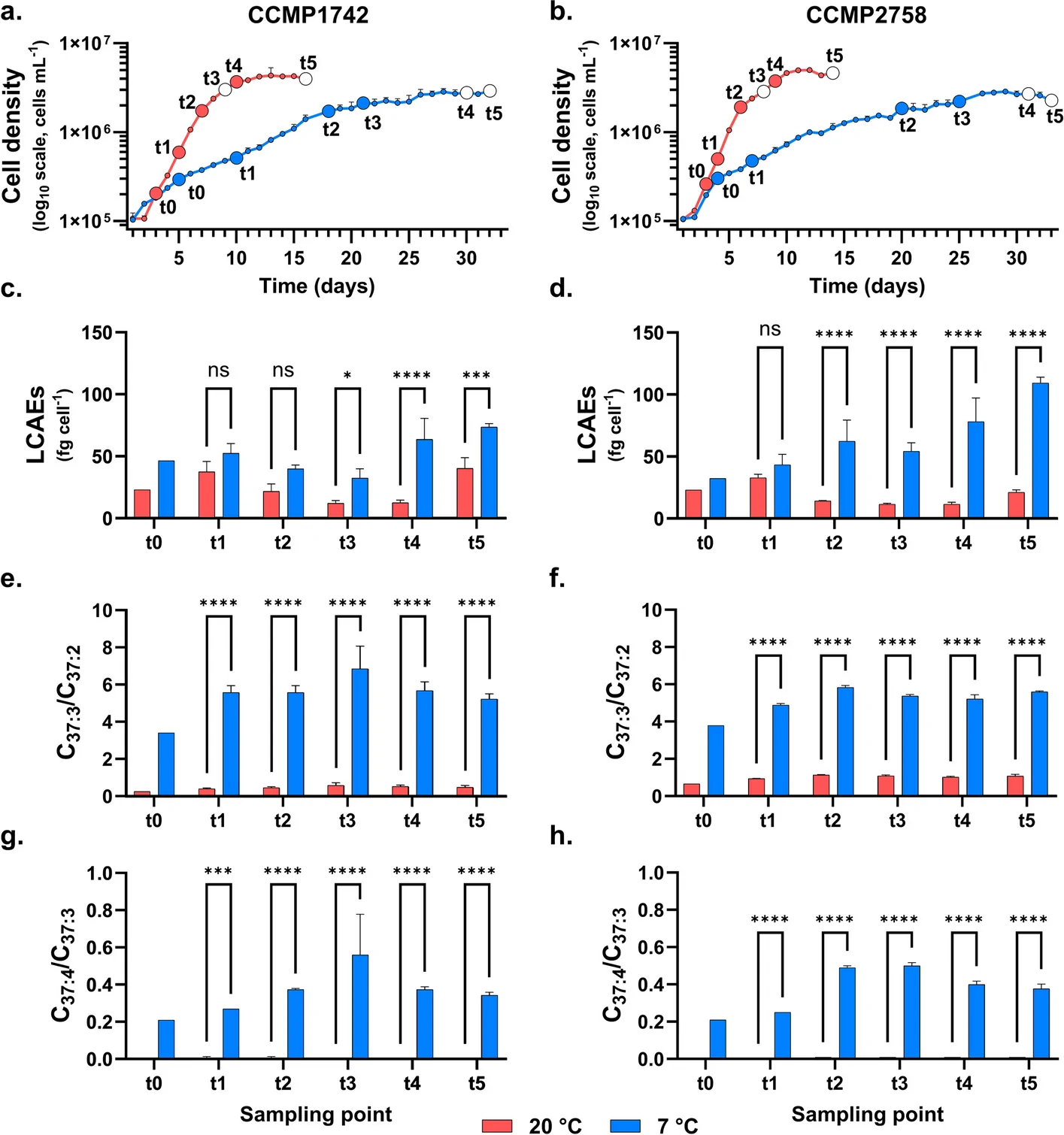
Cell and LCAE abundances and ratios of *G. huxleyi* strains CCMP1742 and CCMP2758 grown at 20 °C and 7 °C. **a**, **b** Cell abundances (raw values displayed on a log_10_-scaled axis). Cultures were sampled for lipid analysis at six time points (t0-t5, large circles) based on cell density and growth stage, large solid circles indicate sampling points used for lipid and RNA extraction, and large empty circles indicate sampling points used only for lipid analysis. **c**, **d** LCAE concentrations (femtograms per cell). **e**, **f** C_37:3_/C_37:2_ MeK LCA ratio. **g**, **h** C_37:4_/C_37:3_ MeK LCA ratio. The x-axis in (**c**-**h**) shows the sampling points from (**a**, **b**). Error bars indicate standard deviations of measurements of three replicates, except at t0 (single sample from the main three-liter culture before distribution into 15 flasks). Significant differences in t1-t5 in panels c-h were tested using a 2-way ANOVA and the Šídák multiple comparisons test. n.s, *p*-adjusted value > 0.05; *, *p*_*adj*_ ≤ 0.05; **, *p*_*adj*_ ≤ 0.01; ***, *p*_*adj*_ ≤ 0.001; ****, *p*_*adj*_ ≤ 0.0001

Because the cold shock experiments did not have the desired effect on the strains from colder environments, we performed additional experiments using strains CCMP1742 and CCMP2758 acclimated*—*i.e. grown at the desired temperature for a period of several weeks prior to the experiment*—*to milder temperatures (20 °C control and 7 °C cold-adapted). To ensure that we find at least one point in which abundances of C_37:3_ and C_37:4_ would be higher at 7 °C than at 20 °C we collected samples at different growth stages. Since LCA concentration per cell has been reported to increase through the exponential and the stationary phase [58, 59], this approach should also allow us to evaluate LCAE synthesis over time and potentially identify the responsible genes. Cultures were grown in three-liter batches in preparation for the experiment and then distributed into smaller flasks at the start of the experiment (t0). Both strains grew slower at 7 °C than at 20 °C, reaching maximum cell density after 31–32 days at 7 °C compared to 12–13 days at 20 °C (Fig. 7 and Figure S16). Thus, given the slower growth observed when strains were grown at 7 °C, triplicate samples were collected after different incubation times (labelled t1-t5) to ensure comparable cell densities (Fig. 7a,b). LCA abundances were analyzed for all sampling points (Table S15).

##### Changes in lipid composition

**Fig. 8:**
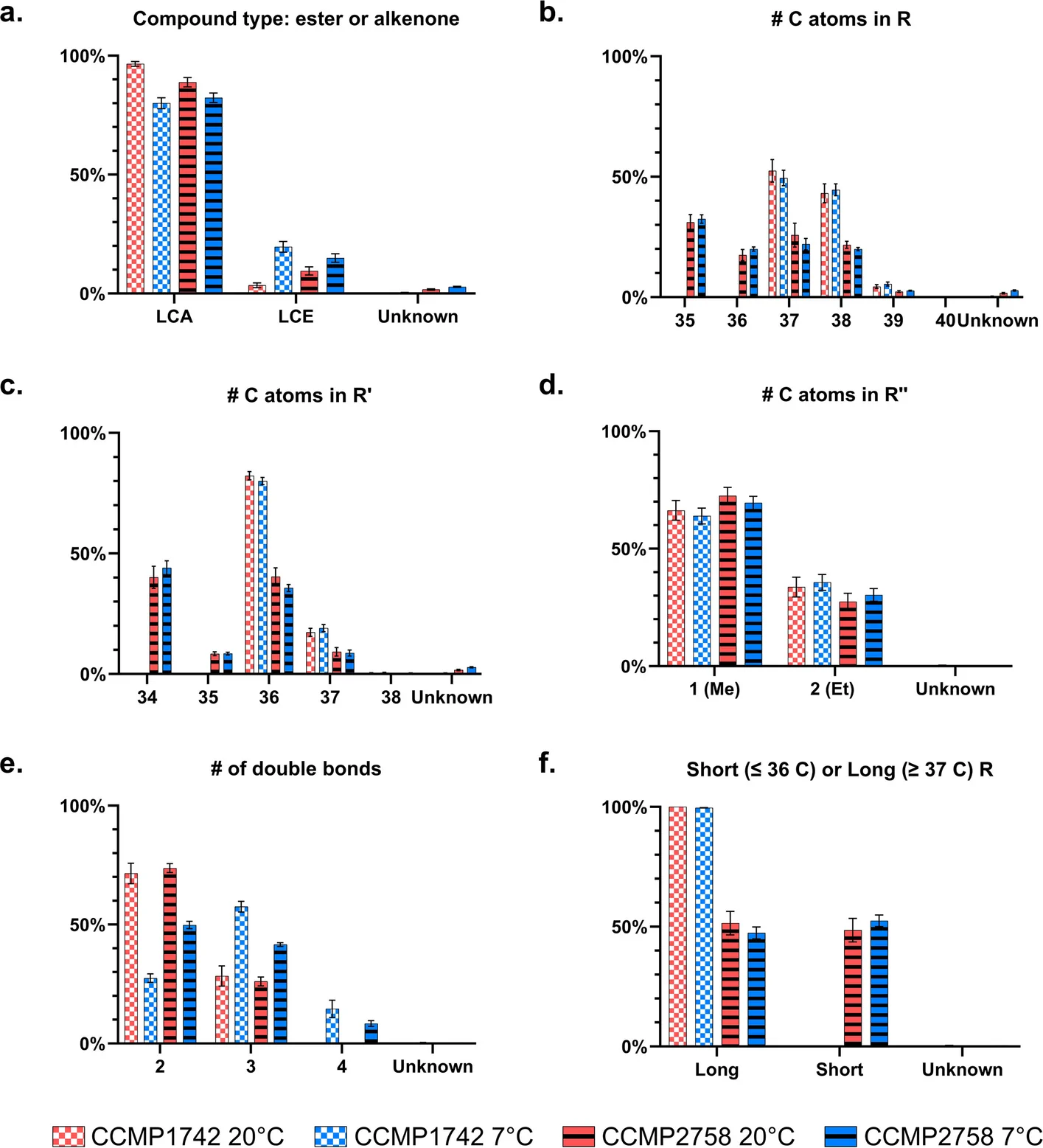
Average percentages across all sampling points of LCAEs in strains CCMP1742 (white checkers) and CCMP2758 (black stripes), grown at 20 °C (red) and 7 °C (blue). As shown in Fig. 1, the total number of carbon atoms of the molecule is represented by R, the longer side of the molecule including the keto group is represented by R’, and the small chain of the molecule is represented by R’’. Panel **a**) shows the average percentage of compounds with two, three, four or an unknown number of double bonds, panel **b**) shows the average percentage of compounds with only a keto group (LCAs) or a keto group and an additional ester group (LCEs), panel **c**) shows the average percentage of compounds that contain a total (R) of 35 to 40 C atoms, panel **d**) shows the average percentage of compounds that contain a total of 34 to 39 C atoms on the long side of the molecule (R’), panel **e**) shows the average percentage of compounds that contain one (Me) or two (Et) carbon atoms on the short side of the molecule (R’), panel **f**) shows the average percentage of compounds containing a total of 35 or 36 C atoms (short) or 37 to 40 C atoms (long). Numerical averages and standard deviations used in this figure can be found in Table S19 section ii. Each bar contains the average of three biological replicates sampled across five time points (t1 to t5) (*n* = 15)

All LCAs and LCEs present in both strains were identified (Fig. 2, Supplementary Information, Figures S17–S22 and Table S16 and S17), and their total cellular abundance (LCAs + LCEs, hereafter referred to as “Long chain alkenones/esters” abbreviated as LCAEs) was quantified across time and between temperatures (Figure S23). For each strain, the temporal variation in LCAE abundance followed a similar trend, and significant differences across samples for which transcriptomic data are also available were only observed at 20 °C (Figure S23a,c). Additionally, significantly higher LCAE concentrations were observed when strains were grown at 7 °C than at 20 °C from t2 (CCMP2758, *p*_*adj*_ ≤ 0.0001) and t3 (CCMP1742, *p*_*adj*_ ≤ 0.05) onwards (Fig. 7c,d). Although the relative abundance of LCEs in both strains when grown at 7 °C increased slightly, LCAs were overall more abundant than LCEs (Fig. 8a, Tables S18, S19, and Figure S24). Both the C_37:3_/C_37:2_ and C_37:4_/C_37:3_ ratios of C_37_ MeKs increased significantly at 7 °C compared to those at 20 °C for both strains and for all sampling points (Fig. 7e-h). The C_37:3_ MeK was always detected at 20 °C, the highest abundances of the C_37:3_ MeK occurred at t1, whilst in cultures grown at 7 °C, the C_37:3_ MeK abundance either decreased or remained stable from t1 to t3 before increasing during t4 and t5 (Figure S25). The C_37:4_ MeK was primarily detected in cultures grown at 7 °C, where its cellular concentration increased over time, whereas only trace amounts were detected at 20 °C (Table S16 and Figure S25).

In addition to modifying their degree of unsaturation, lipids can also adapt to temperature changes by altering their acyl chain length [60, 61]. Our analysis revealed that whilst colder temperatures favor biosynthesis of LCEs, LCAE length remained stable, thus suggesting that the number of elongation rounds is not influenced by growth stage nor temperature (Supplementary Information, Fig. 8b-e and Figures S17, S24, S26–S28, and Tables S18 and S19). However, comparison between strains shows that while both strains synthesize LCAEs containing 37 to 39 C atoms (C_37–39_), the proportion of shorter LCAEs containing 35 and 36 C atoms (C_35–36_) in CCMP2758 accounts for approximately 50% of the LCAEs produced (Fig. 8f, Figure S29).

##### Changes in gene expression of FAS and PKSs

Gene expression (transcriptomic) data were determined for three time points in each experiment (7 °C; t1, t2 and t3, and 20 °C; t1, t2 and t4, Figure S16 and Table S15). At 20 °C a significant decrease in the cellular concentrations of LCAEs was observed from t1 to t4, followed by an increase at t5, (Figure S23). We assume that this change in concentration coincides with a change in expression of the gene involved in LCAE formation. Since changes in phenotype can lag behind changes in gene expression [62], our analysis comprised genes that were either up- or downregulated across time (t1 vs t4) for the cultures grown at 20 °C. Additionally, when strains were grown at 7 °C significantly higher concentrations of LCAEs (compared to 20 °C) were noted from t2 (CCMP2758, *p*_*adj*_ ≤ 0.0001) and t3 (CCMP1742, *p*_*adj*_ ≤ 0.05) onwards (Fig. 7c,d, Data S6). Therefore, we focused our differential gene expression analysis on PKSs and FAS-related genes (Table 2 and Table S10)—including genes closely related to PKS7—that were differentially expressed across time at 20 °C (t4 20 °C vs t1 20 °C), and between temperatures at t2 or t3/t4 (t2 7 °C vs t2 20 °C, and t3 7 °C vs t4 20 °C) (Supplementary Information, Figures S30–S32, Table S20, Data S6 and Data S7).

The results of differential gene expression analysis showed that 36 PKS- and FAS-related genes in CCMP1742 and 76 in CCMP2758 were downregulated at t4 compared to t1 when grown at 20 °C (Supplementary Information and, Tables S21 and S22). For the differential gene expression at 7 °C vs 20 °C, we observed that 66 PKS and FAS-related genes in CCMP1742 and 76 in CCMP2758 were upregulated at 7 °C coinciding with higher LCAE abundances (Figure S23 and 30, Table S21). These genes include the largest PKS7 homolog identified in the CCMP2758 genome (the gene encoding BGC-CCMP2758_FUN_000054-T1, 13,548 aa). Only 12 genes in CCMP1742 and 53 in CCMP2758—most related to PKSs—were differentially expressed under both conditions (Table S22). Of the genes that were differentially expressed across temperatures and time, two in CCMP1742 and 30 in CCMP2758 encoded PKS7 homologs (Table S10, S21, and S22), of which the largest were a 8,918 aa protein (BGC-CCMP2758_FUN_000094-T1) and the two sets of identical 10,886 aa and 10,904 aa protein pairs in CCMP2758 that originated from the same BGC as discussed earlier (i.e. BGC-CCMP2758_FUN_000059-T1 = BGC-CCMP2758_FUN_000060-T1, and BGC-CCMP2758_FUN_000061-T1 = BGC-CCMP2758_FUN_000062-T1). These four proteins may represent a single gene whose sequence was ambiguously resolved during assembly, or two highly similar genes that were collapsed in the assembly. All the other hits were considerably shorter (Table S10). Given the length of these putative proteins, their annotated functions, their similarity to PKS7 and their differential gene expression—which correlates with changes in LCAE concentration—these experiments provide support for our hypothesis that these proteins might be involved in the first step of LCAE formation.

*G. huxleyi* is a diploid organism; it carries two sets of chromosomes and thus two alleles of each gene. Given that CCMP2758 originated from CCMP1742 [30], and that the uncommon short-chain (C_35_-C_36_) LCAEs constitute approximately 50% of the total LCAEs in CCMP2758 (Fig. 8f and Figure S29), we propose that the biosynthesis of these shorter LCAEs is the result of a mutation in one of the alleles involved in LCAE formation. In this hypothesis, CCMP1742 would carry two copies of the original or *wild type* (*wt*) gene responsible for the synthesis of C_37–39_ LCAEs, whereas CCMP2758 would carry a copy of the *wt* allele responsible for the synthesis of C_37–39_ LCAEs, and an altered allele responsible for the synthesis of the shorter C_35–36_ LCAEs characteristic of CCMP2758. Given the shorter distance (two methylene units less) between the second double bond and the carbonyl group in the ∆^12,19^ C_35–36_ LCAs versus the ∆^14,21^ C_37–38_ LCAs, this mutation may have resulted in the lack of one of the modules responsible for the addition of one Malonyl-CoA extender unit after the second double bond has been introduced (Figure S7b). The three large CCMP2758 genes differently expressed across time and temperatures identified here (the gene encoding BGC-CCMP2758_FUN_000094-T1 and the BGC with two sets of identical predicted protein pairs) may represent that allelic diversity. Additionally, the three distinct predicted proteins could correspond to an incomplete assembly of the full PKS7 homolog, with approximately half of the sequence encoded by BGC-CCMP2758_FUN_000094-T1, and the other half—possibly representing the allelic diversity—encoded by the other BGC (BGC-CCMP2758_FUN_000059-T1 or BGC-CCMP2758_FUN_000061-T1). This would result in a large gene with two potential alleles that encodes proteins of a similar size to PKS7: BGC-CCMP2758_FUN_000094-T1::BGC-CCMP2758_FUN_000059-T1 (19,804 aa) and CCMP2758_FUN_000094-T1::BGC-CCMP2758_FUN_000061-T1 (19,822 aa). Alternatively, shorter PKS7 homologs identified here may be responsible for LCA and LCE formation, for example because they resemble a highly incomplete assembly of the full gene. Further long-read sequencing efforts of the CCMP1742 and CCMP2758 genomes are required to resolve these PKS sequences.

The correlation between total LCAE abundance and the expression pattern across time and temperature of the candidate genes proposed here, their functional annotation, and (in some cases) sequence similarity to PKS7 suggests their involvement in LCAE formation, making them good targets for future functional pathway characterization studies—via for example knockouts—that could definitively determine the genes that are involved in LCAE formation and the mechanisms regulating their length (Supplementary Information and Tables S21–S25).

##### Changes in gene expression of desaturases

**Fig. 9:**
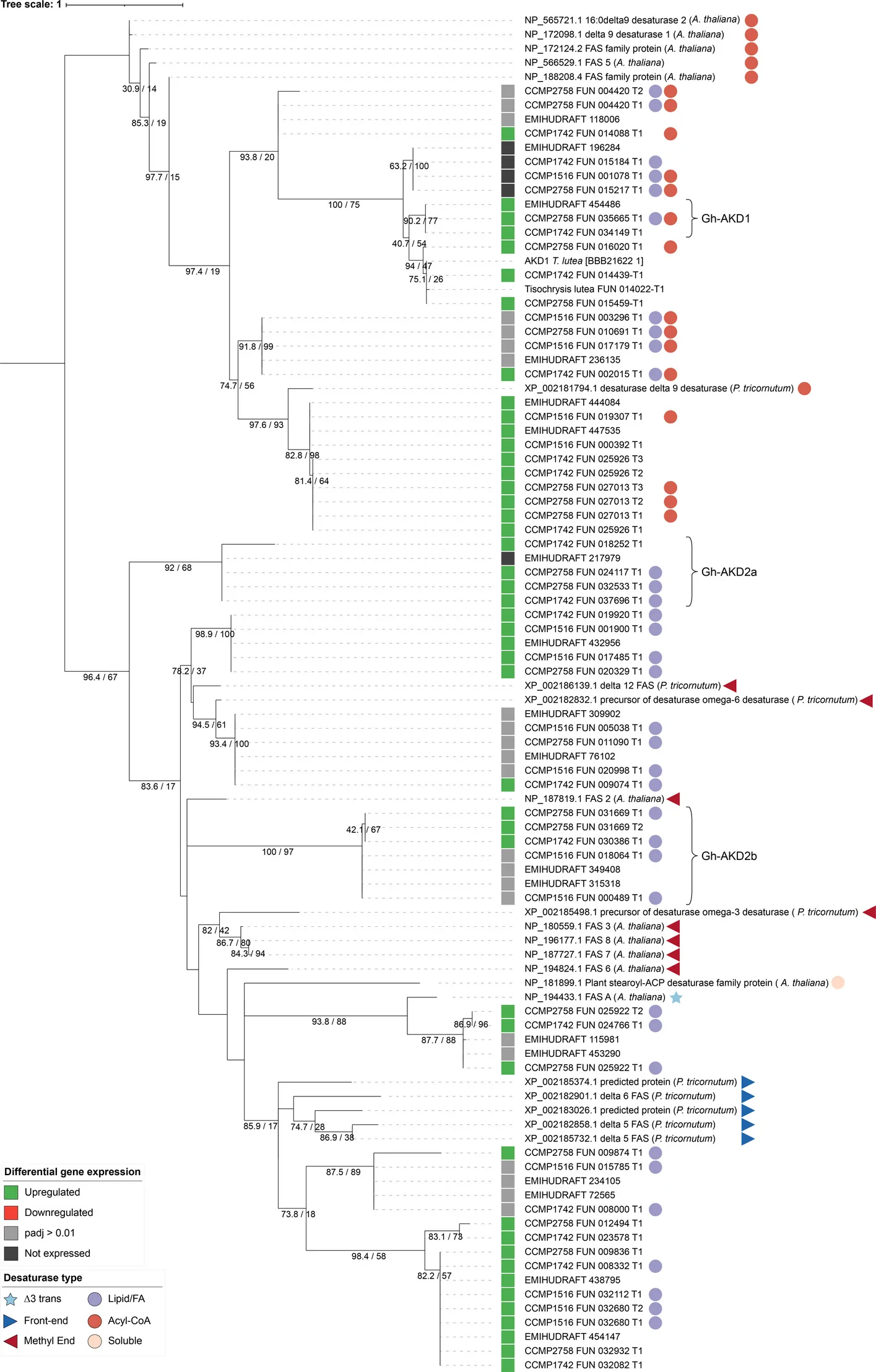
Phylogenetic protein tree showing the *Tisochrysis lutea* AKD1 (bold), a subset of the already characterized or annotated desaturases from *Phaeodactylum tricornutum* and *Arabidopsis thaliana* used in Supplementary Fig. 1 in ref [24], the *G. huxleyi* CCMP1742 and CCMP2758 upregulated genes annotated as desaturases, and their orthologues in CCMP1516, CCMP1742 and CCMP2758. Data S8 contains the source files. Coloured squares indicate the differential expression of each gene. Differential gene expression annotation is shown on a per gene basis, therefore different protein isoforms originating from the same gene but noted differently by their “T” identifier will be represented as having the same differential gene expression, and thus will be shown as a square of the same colour. Coloured circles, rectangles and stars indicate the annotated function of each desaturase. The tree was generated using the substitution model PMB + R3 as selected by Model Finder based on the Bayesian Information Criterion in IQ-tree. Values on branches indicate non-parametric bootstrap supports (left side of the slash) and the SH-aLRT (right side of the slash), only values > 10 are shown. Scale bar represents the mean number of substitutions per site. The tree is rooted at midpoint

Next, we focused on the identification of the genes encoding the proteins responsible for the introduction of the third and fourth double bond of LCAs in *G. huxleyi* CCMP1742 and CCMP2758, hereafter referred to as Gh-AKD1 and Gh-AKD2 (*Gephyrocapsa huxleyi* Alkenone desaturase 1 and 2). We first identified all the genes annotated as desaturases that were significantly overexpressed at 7 °C. A striking difference in the number of annotated desaturases between the closely related strains CCMP1742 and CCMP2758 was detected. Therefore, we performed reciprocal homology searches using OrthoFinder for each overexpressed desaturase (Table S26). This approach ensured detection even if orthologs were misannotated and were thus not identified as desaturases based on their functional annotation—for example, if GeneX were an overexpressed desaturase in CCMP1742, and GeneY its unannotated ortholog in CCMP2758, both genes would be identified using this approach. Additionally, for comparison we also searched for homologs of these annotated desaturases in CCMP1516. To evaluate potential annotation pipeline effects on our results, we performed these homolog searches in both the CCMP1516 reference annotation and the Funannotate-derived annotation. This comparison could reveal potential artifacts arising from the use of different annotation pipelines, such as cases where a gene is present in only one of the annotations, or cases where a single gene in the reference annotation appears as two in its Funannotate version. To improve interpretability and infer the potential function of these proteins based on sequence similarity and annotated function, we constructed a phylogenetic tree using the identified sequences, along with AKD1, EMIHUDRAFT_454486 and its orthologues, and a set of characterized and annotated desaturases from *Arabidopsis thaliana* and the pennate diatom *Phaeodactylum tricornutum* (a dataset previously used in the identification of AKD1 in *T. lutea* [24]) (Fig. 9, Figure S33, Table S27, and Data S8). This revealed that BGC-CCMP1742_FUN_034149-T1 and BGC-CCMP2758_FUN_035665-T1 (two nearly identical proteins which slightly differ in length) are the closest homologs to EMIHUDRAFT_454486 and are also, not unexpectedly, upregulated when grown at 7 °C (Fig. 9, Figures S33–S35, Table S26 and S27). This indicates that these two genes likely encode the desaturases responsible for introducing the third unsaturation (∆^7^, Gh-AKD1) in strains CCMP1742 and CCMP2758.

While the absence of detectable C_37:4_ LCA in CCMP1516 suggests that the protein responsible for introducing the fourth unsaturation (∆^28^, Gh-AKD2) may not be synthesized, the gene could still be present in the genome but remain unexpressed or expressed at low levels—due to for example mutations in the untranslated regions, promoter regions, in potential enhancer sequences or by mutations affecting RNA stability—rendering C_37:4_ LCA undetectable. To identify Gh-AKD2 we analyzed putative desaturases that were overexpressed in strains CCMP1742 and CCMP2758 when cultures were grown at 7 °C but absent or not differentially expressed in CCMP1516. We identified two phylogenetic clusters that followed these criteria and could contain Gh-AKD2 (Fig. 9 and Figure S33). Cluster Gh-AKD2a contains a non-expressed CCMP1516 protein (EMIHUDRAFT_217979), two upregulated CCMP2758 proteins annotated by Funannotate as fatty acid desaturases (BGC-CCMP2758_FUN_024117-T1 and BGC-CCMP2758_FUN_032533-T1) and two upregulated CCMP1742 proteins, one also annotated as a fatty acid desaturase (BGC-CCMP1742_FUN_037696-T1) and one without an annotated function (BGC-CCMP1742_FUN_018252-T1, Table S27). Even though the cluster contains a protein from the CCMP1516 reference genome, it does not contain any protein from the CCMP1516 genome annotated with Funnanotate, showing that its gene prediction models might have overlooked this (and potentially other) gene(s). Cluster Gh-AKD2b contains two CCMP1516 proteins that were not differentially expressed, annotated as EMIHUDRAFT_349408 and EMIHUDRAFT_315318 when using the reference annotation, and as CCMP1516_FUN_000489-T1 and CCMP1516_FUN_018064-T1 when annotated with Funannotate, one upregulated CCMP1742 putative desaturase (BGC-CCMP1742_FUN_030386-T1) and two upregulated CCMP2758 proteins, which are isoforms of the same gene (BGC-CCMP2758_FUN_031669-T1 and -T2) (Table S27). The expression pattern of the proteins found in these two clusters suggests that these genes could be Gh-AKD2 and makes them good candidates for future enzyme characterization studies.

## Conclusion

In this study, we evaluated changes in LCA production of three different *G. huxleyi* strains under different conditions, combined with transcriptome analyses to identify differentially expressed genes that may be associated with those changes. Given that CCMP1516 was the only strain with an available annotated genome at the start of this study and does not produce tetra-unsaturated nor shorter (C_35_ and C_36_) LCAs, we sequenced non-axenic strains CCMP1742 and CCMP2758, and developed a computational approach tailored to remove contamination and obtain substantially complete draft genome assemblies for algae. Potential gene candidates for the formation of LCAEs as well as for the addition of the third and fourth double bonds were identified based on differential expression, homology searches, phylogenetics, and detailed analysis of PKS protein modules. Based on these results, we propose a new biosynthetic pathway for LCAE biosynthesis.

Based on its structure and the similarity of its predicted biosynthetic compounds to LCAEs, we identified the PKS7 protein as potential candidate involved in the first step of LCAE formation generating LCAEs with two *trans* double bonds (i.e., C_37:2_ MeK). A subset of similar proteins was identified in CCMP1742 and CCMP2758 (BGC-CCMP2758_FUN_000094-T1, CCMP2758_FUN_000059-T1 to BGC-CCMP2758_FUN_000062) based on their annotated function and expression pattern (Supplementary Table 22). We also identified EMIHUDRAFT_454486 as the desaturase responsible for the introduction of the third unsaturation (∆^7^) in CCMP1516, which was experimentally confirmed recently in another study [57], as well as corresponding homologs in CCMP1742 (BGC-CCMP1742_FUN_034149-T1) and CCMP2758 (BGC-CCMP2758_FUN_035665-T1). Based on their functionality, sequence similarity and expression pattern, potential genes that could be responsible for introducing the fourth unsaturation (∆^28^) in C_37_ LCAs were also identified in CCMP1742 (BGC-CCMP1742_FUN_037696-T1, BGC-CCMP1742_FUN_018252-T1, and BGC-CCMP1742_FUN_030386-T1) and CCMP2758 (BGC-CCMP2758_FUN_024117-T1, BGC-CCMP2758_FUN_032533-T1, BGC-CCMP2758_FUN_031669-T1 and -T2).

To confirm whether these genes are involved in LCA biosynthesis and confirm our proposed biosynthetic pathway, further in vivo experiments, such as heterologous expression or deletion of the candidate gene(s), are required. While in vivo experiments are still challenging in *G. huxleyi* due to the lack of a stable genetic transformation system, they can already be achieved using the transformable and alkenone-producing haptophyte *T. lutea* and its recently developed heterologous expression system [24, 57]. Thus, our study constitutes a stepping stone on the journey towards the complete elucidation of the LCA biosynthetic pathway.

## Material and methods

### Strains and culture conditions

Three strains of the haptophyte *Gephyrocapsa huxleyi*—CCMP1516, CCMP1742 and CCMP2758—were obtained from the National Center for Marine Algae and Microbiota (NCMA), and cultured in L1-Si medium [63]. All cultures were non-axenic. CCMP1742 and CCMP2758 cultures were treated with antibiotics to remove the contaminants albeit unsuccessfully (Supplementary Information). Cell growth was monitored by measuring cell abundances of fresh samples using a Beckton Dickinson Accuri™ C6 flow cytometer (BD Biosciences, New Jersey, US) equipped with a 488 nm argon laser, with the trigger set on chlorophyll red autofluorescence (FL4 filter, 675 ± 25 nm). The phytoplankton cells were distinguished in a scatter plot using the chlorophyll red autofluorescence versus forward side scatter. The raw flow cytometry counts are available in Data S5.

### Growth experiments

#### Cold shock experiments

*G. huxleyi* strain CCMP1516 was initially grown in batch culture in five-liter glass Erlenmeyer flasks at 20 °C, under a light:dark cycle of 16:8 h at 100–170 μmol photons m^−2^ s^−1^. When the culture had reached a cell density of 6.5 × 10^5^ cells ml^−1^ it was split in 250 mL Erlenmeyer flasks, and kept at 20 °C for 1 day, after which half of the Erlenmeyer flasks were randomly selected and immediately transferred to a room at 10 °C with the same light regime and light–dark cycle. Immediately after the transfer, three Erlenmeyer flasks maintained at 20 °C were filtered over different pre-combusted glass fiber filters (T0, GF/F pore-size of 0.7 µm, 47 mm in diameter. Whatman, Maidstone, UK) using a vacuum pump. Filters were flash-frozen in liquid nitrogen immediately after filtration and stored at −80 °C until further processing.

Eight-liter cultures of *G. huxleyi* strains CCMP1742 and CCMP2758 were grown in a 10-L polycarbonate bottle at 15 °C under a 16:8 light:dark regime at 100–170 μmol photons m^−2^ s^−1^. Cultures were continuously stirred and air was constantly provided using an aquarium air pump through a 0.2 µm pore size filter (Sartorius, Göttingen, Germany). When the culture reached a cell density of 1.7–2.5 × 10^5^ cells mL^−1^ it was split in 250 mL Erlenmeyer flasks and kept at 15 °C overnight, then half of the flasks were transferred to 3 °C under a 16:8 light:dark regime at 80–120 μmol photons m^−2^ s^−1^. Immediately after transferring the flasks to 3 °C, the contents of three Erlenmeyer flasks were harvested via filtration. Filters were then wrapped in aluminum foil and immediately stored at −80 °C (see Supplementary Information for details).

#### Temperature adaptation experiments

Cultures were acclimated from 15 °C to 20 °C and 7 °C for at least six months prior to the experiments. Three-liter cultures were grown in five-liter Nalgene bottles under the same light intensity, light regime and stirring conditions described above (see Supplementary Information for details). Cell growth was monitored daily using flow cytometry. Upon reaching a cell density of 1.8–2.8 × 10^5^ cells mL^−1^, the three-liter culture was distributed in 160 mL aliquots amongst fifteen 250 mL Erlenmeyer flasks with a wide opening. To capture differences in the metabolic state of the cells at different growth stages, cultures were harvested at six different sampling (T) points based on their cell densities; T0 (1.8–2.8 × 10^5^ cells mL^−1^), T1 (4.7–5.6 × 10^5^ cells mL^−1^), T2 (1.5–1.9 × 10^6^ cells mL^−1^), T3 (1.8–2.9 × 10^6^ cells mL^−1^), T4 (2–3.7 × 10^6^ cells mL^−1^), T5 (2.3–3.4 × 10^6^ cells mL^−1^). Cells were harvested by filtering 50 mL of culture over GF/F filters as described above and immediately stored at −80 °C until further processing.

#### Culturing of algal biomass for DNA extraction for Nanopore and Illumina sequencing

Eight liters of CCMP1742 and CCMP2758 cultures were grown in 10-L polycarbonate bottles using the same cultivation set up as described for the cold shock experiments, five to six liters were harvested using a continuous centrifuge (Heraeus Contrifuge Stratos, Thermo Electron LED, Osterode, Germany), washed and used for extraction of DNA for long read Nanopore and short read Illumina sequencing (see Supplementary Information for details).

### Lipid extraction and analysis

LCAs and LCEs were extracted using half of each GF/F filter as described elsewhere [64] (see Supplementary Information for details). Ketone fractions of *G. huxleyi* CCMP1516 were analyzed using an Agilent 6890 gas chromatography (GC) instrument equipped with an Agilent CP-Sil 5 CB column (50 m × 0.32 mm i.d.; 0.12 µm film thickness). The temperature program was: 70–200 °C at 20 °C min^−1^, then at 3 °C min^−1^ to 320 °C (held for 25 min). The ketone fractions of *G. huxleyi* CCMP1742 and CCMP2758 were analyzed using an Agilent 7890B GC with a flame ionization detector equipped with an RTX-200 column (Restek, 60 m × 0.32 mm × 0.5 µm). Compounds were identified using the same GC instrument and column coupled to a mass spectrometer (GC–MS, Agilent Technologies 7000 C GC/MS Triple Quad), using the following temperature program: 70 °C-250°C at 15 °C/min, 250–320 °C at 1.5 °C/min, then kept at 320 °C for 25 min at a flow rate of 1.5 mL min^−1^. Peaks containing coeluting LCAs and LCEs were disentangled by removing LCEs from the ketone fraction using base hydrolysis (see Supplementary Information for details). Compounds present in base hydrolyzed extracts were identified using GC–MS as described above. To correct for shifts in retention times, GC peak data from multiple files were aligned using GCalignR (v 1.0.3) [65] followed by manual curation. Raw GC-FID and GC–MS datafiles are available in Data S1.

### Nucleic acids extraction and sequencing

RNA was extracted from selected samples using ½ or ¼ of each GF/F filter using the Qiagen RNeasy mini kit (Qiagen, Valencia, CA, USA) according to the manufacturer’s instructions with minor modifications (see Supplementary Information for details). PCR was performed on RNA extracts to confirm absence of DNA. RNA concentrations were measured using Nanodrop ND-1000 Spectrophotometer (NanoDrop Technologies Inc. Wilmington, DE, USA) or Qubit RNA HS Assay Kit on a Qubit 3.0 Fluorometer (Qubit, Thermo Fischer Scientific, Waltham, MA, USA). The quality and integrity of the RNA extracts was assessed using an Agilent RNA 6000 Nano Kit on a Bioanalyzer 2100 (Agilent Technologies, Santa Clara, CA, USA).

TruSeq Stranded mRNA polyA library preparations were carried out by Utrecht Sequencing Facility (USEQ, Utrecht, The Netherlands). RNA fragments of CCMP1742 and CCMP2758 were barcoded using Unique Molecular Identifiers (UMIs) (9 bp length) during library preparation. Samples were sequenced using either two runs of 1 × 75 bp on an Illumina Nextseq 500 platform (CCMP1516) or a S2, 2 × 150 bp run on an Illumina NovaSeq 6000 platform (CCMP1742 and CCMP2758). 

High molecular weight DNA was extracted from strains CCMP1742 and CCMP2758 using CTAB method. DNA library preparations and sequencing of the genomic extracts were carried out by USEQ. Each strain was sequenced using individual Nanopore MinION runs (Oxford Nanopore Technologies, Oxford, UK) and one Illumina NextSeq 500 Mid output flow cell, 2 × 150 bp (see Supplementary Information for details).

### Genome assemblies of CCMP1742 and CCMP2758

Quality of Nanopore reads was assessed using pycoQC [66] and Nanoplot (v 1.33.1) [67], only reads with Q ≥ 7 were used. Low quality bases (Phred score ≤ 33) and adapter sequences from Illumina short reads were removed using Trimmomatic (v 0.36) [68] keeping only reads with a minimal length of 40 bp, and remaining adapters and polyG tails were removed using Cutadapt (v 1.16) [69]. Quality of the resulting trimmed sequences was evaluated using FastQC [70].

Several genome assemblers were initially tested to determine the most suitable tool for our datasets (Data S2). These were long read assemblers: CANU (v 1.8) [71–77], flye (v 2.8.1) [78], shasta (v 0.5.1) [79] and wtdbg2 (v 2.3) plus consenser wtpoa-cns (v 2.3) [80]; short read assemblers (SPAdes (v 3.14.1) [81, 82]); and hybrid assemblers using short and long reads: Wengan (v 0.2) [83]—using DiscovarDeNovo (WenganD) or Minia3 (WenganM) as short read assemblers—hybridSPAdes (v 3.14.1) [34], biosyntheticSPAdes (v 3.14.1) [35] and HASLR (v 0.8a1) [84]; and a metagenomic assembler (OPERA-MS (v 0.8.2) in hybrid mode using MegaHIT as short read assembler) (Figure S2). Additionally we tested two scaffolders: WenganD and OPERA-LG (v 2.0.5) [36, 85] on two selected assemblies (hybridSPAdes and biosyntheticSPAdes), and tested WenganD alone on a third assembly (Short read SPAdes). The quality of the assemblies was assessed using Quast (v 5.0.2) [86], and the presence of bacterial contigs was evaluated using a combination of parameters: taxonomic classification of each contig, using CAT (v 5.2.3, and running Prodigal (v 2.6.3) [87] and DIAMOND (v 2.0.6) [88]) [89], contig coverage using Minimap2 (v 2.17) [90] and the GC content of each contig using a custom script.

De novo assembly of CCMP1742 and CCMP2758 was carried out by (1) assembling the reads into metagenomes using two metagenomic hybrid assemblers; MetaSPAdes (v 3.14.1) [33] and OPERA-MS, using SPAdes as short read assembler (2) mapping the reads to the assembly using Minimap2 (3) Identifying contigs from likely contamination using the CAT classification, contig coverage and GC content (4) removing reads mapping to non-eukaryotic contigs (5) generating a hybrid assembly with SPAdes and biosyntheticSPAdes using only the selected reads (6) using OPERA-LG to generate scaffolds (7) identifying non-eukaryotic scaffolds as described above and (7) removing non-eukaryotic scaffolds (Fig. 3, Figure S3, and Data S3). Quality of the resulting assemblies was assessed using QUAST, BUSCO (v 5.1.1) with the OrthoDB v10 (odb10) database [37, 91], CAT, scaffold coverage and GC content (Figure S5). Based on these quality assessments we chose the assemblies obtained using metaSPAdes as metagenomic assembler.

Genome assemblies were functionally annotated using Funannotate (v 1.8.7) [38] incorporating RNA-seq data from Illumina reads and a transcriptome generated using Trinity (v 2.11.0) [92] to improve the accuracy of gene model predictions (see Supplementary Information for a detailed description of the commands used during genome annotation). Prior to functional annotation the “gene clusters” file generated by biosyntheticSPAdes and containing the BGCs, was concatenated with the main assembly file. To identify potential artificial duplications arising from this approach we carried out a blastN search against the mRNA transcripts of the annotated genome using the genes that originated from the “gene_clusters” file as query. Funannotate output files are provided in Data S4.

### Genome comparison and identification of candidate genes involved in LCA biosynthesis

Analysis of orthologous gene clusters amongst selected haptophytes was carried out using the OrthoFinder [40] algorithm within OrthoVenn3 [39] with default parameters (E-value: 1e-2, Inflation value: 1.5). The genomes compared included the genomes *G. huxleyi* strains CCMP1742 and CCMP2758 (sequenced in this study), *G. huxleyi* CCMP1516 [26] and *T. lutea* CCAP 926/14 [93] annotated with Funannotate and additionally the reference annotation of CCMP1516 (GCF_000372725.1). Genomes annotated with Funannotate were also compared using the ‘Funannotate compare’ utility.

To identify genes potentially involved in LCA desaturation we searched the genome annotations using the inclusion and exclusion keywords described in Table S28 (Supplementary Information).

### Differential gene expression and visualization

Low quality bases (Phred score ≤ 33) and adapter sequences from Illumina short reads obtained from RNA extracts were removed using Trimmomatic (v 0.36) [68] keeping only reads with a minimal length of 36 bp for single-end data and 40 bp for paired-end data (Figure S4). Remaining adapters and polyG tails were removed using Cutadapt (v 1.16) [69]. Quality of the resulting trimmed sequences was evaluated using FastQC (v0.11.9) [70].

UMI sequences from CCMP1742 and CCMP2758 transcriptomic reads were added to the FASTQ header using Barcodex (v 1.0.9) [94] (Data S7). RNA-seq data were aligned to the genome using the Spliced Transcripts Alignment to a Reference (STAR) tool [95]. PCR duplicates within CCMP1742 and CCMP2758 RNA reads (49–90% of the total reads, Data S7) were removed using UMI-tools ’dedup’ command (v 1.1.1) [96] and aligned again using STAR to the reference genome or to the genomes assembled in this study. Transcripts were quantified by generating a transcript database in R using the makeTxDbFromGFF function from the GenomicFeatures package (version 1.50.4) [97], followed by grouping using the exonsBy function. Then reads were assigned to the transcripts in this database using the function summarizeOverlaps from the GenomicAlignments package (v 1.34.1) [97]. Normalization of read counts and differential gene expression analysis were carried out using the DESeq2 package (v 1.38.3) [98]. Importantly, differential expression was analyzed separately for each strain, with comparisons made only within each strain's own experimental contrasts (e.g., cold-shocked vs. control for CCMP1516; cold-shocked vs. control for CCMP1742; etc.). No direct quantitative comparisons of expression levels were performed across strains, as differences in sequencing platforms (NextSeq 500 single-end 75 bp for CCMP1516 vs. NovaSeq 6000 paired-end 150 bp with UMIs for CCMP1742 and CCMP2758), library preparation, and duplicate removal strategies make such cross-strain comparisons unreliable. The presence of outliers was evaluated by assessing differences in gene expression between the control and cold-shocked or cold-adapted cultures using principal component analysis (PCA) on variance stabilizing transformed (vst) normalized gene counts and Cook’s distance (Supplementary Information, Figures S11, S12, S31 and S32) [99]. When outliers were detected they were removed and the PCA and Cook’s distance analysis were repeated. Differential gene expression was carried out using a log fold change estimate ≤ −1 or ≥ 1 and a false discovery rate-adjusted p-value (FDR-*p*_*adj*_) ≤ 0.01 or ≤ 0.05 (see Supplementary Information for details). We used different significance thresholds for the two transcriptome screens to balance confidence with discovery. For desaturases involved in adding the third and fourth double bonds, the effect of temperature was strong and consistent—cold temperatures reliably induced tri- and tetra-unsaturated LCAs—so we applied a stricter threshold (FDR-*p*_*adj*_ ≤ 0.01). In contrast, the search for genes involved in LCA biosynthesis was more exploratory. LCA concentrations fluctuated over time, with levels generally decreasing per cell until t3/t4 (except in CCMP2758 at 7 °C) followed by an increase. To minimize the risk of missing relevant genes due to an overly restrictive cutoff, we used a lower threshold (FDR-*p*_*adj*_ ≤ 0.05) for this search. For temperature comparisons, samples grown at 20 °C served as the reference. For time-course comparisons, samples collected at t1 were used as the reference. Transcripts from all sampling points were included for strain CCMP1516, while only the first time point (t1) of strains CCMP1742 and CCMP2758 was analyzed. We used the same bioinformatics settings for all samples within each strain, ensuring that comparisons between control and treated samples are valid within each strain. The R package ggplot2 (v 3.5.1) [100] was used for the visualization of gene counts and principal component analyses (PCA), and the pheatmap package (v 1.0.12) [101] was used for generating heatmap figures. Cell and lipid abundances were plotted using GraphPad Prism v10.4.2 for Windows.

Protein sequences in the phylogenetic tree (Fig. 9 and Figure S33) were aligned using MAFFT (v7.407) with the L-INS-i iterative refinement method [102] and poorly aligned regions were removed using trimAl [103]. The phylogenetic tree was built using IQ-tree (v 1.6.7) and its in-built nucleotide substitution Model Finder [104], using 1,000 replicates to perform SH-like approximate likelihood ratio test (SH-aLRT) [105] and 1,000 bootstrap replicates, and visualized using iTOL (v 6.7.4) [106].

## Supplementary Information

Supplementary Material 1.

Supplementary Material 2.

## Data Availability

*G. huxleyi* CCMP1516 genome sequence and annotation can be found under National Center for Biotechnology Information (NCBI) Refseq assembly accession number GCF_000372725.1. *T. lutea* CCAP 926/14 contigs were obtained from the SEANOE databank (https:/www.seanoe.org/data/00361/47171/data/47136.fasta) and *T. lutea* transcriptomic reads were obtained from NCBI BioProject PRJNA196557. Sequencing data and genome assemblies generated in this study were deposited in the European Nucleotide Archive (ENA) under project number PRJEB70595. The datasets generated and analyzed in the current study are provided as Supplementary Data S1–S8 in the Zenodo repository 10.5281/zenodo.15844033.

## Abbreviations

LCAs: Long-chain alkenones
LCEs: Long-chain esters
PKSs: Polyketide synthases
FAS: Fatty acid synthases
MeK: Methyl ketone
EtK: Ethyl ketone
SST: Sea Surface Temperature
ACP: Acyl Carrier Protein
KS: Ketosynthase
KR: Ketoreductase
DH: Dehydratase
ER: Enoylreductase
AT: Acyltransferase
BGCs: Biosynthetic Gene Clusters
Bp: Base pairs
Mbp: Mega base pairs
AKD1: Alkenone desaturase 1
Gh-AKD: *Gephyrocapsa* *huxleyi* Alkenone desaturase
