# Supplementary material for: New genomic insights into long-chain alkenone biosynthesis by the coccolithophorid marine alga Gephyrocapsa huxleyi
Source: BMC Genomics. 2026 May 30;27:711. doi: 10.1186/s12864-026-12930-5 (PMC13508280; doi:10.1186/s12864-026-12930-5)
Supplement: Supplementary file 1 [file 12864_2026_12930_MOESM2_ESM.pdf]

New genomic insights into long-chain alkenone biosynthesis by the coccolithophorid marine alga *Gephyrocapsa huxleyi*

Ruth Pérez Gallego, F. A. Bastiaan von Meijenfeldt,
Marc A. Besseling, Marcel T. J. van der Meer,
Jaap S. Sinninghe Damsté, and Laura Villanueva

Supplementary Information

Includes
Supplementary Results and Discussion,
Supplementary Materials and Methods,
Supplementary Figs. S1-S35,
and 70 Supplementary References.

Supplementary Tables S1-S28 are supplied as an additional file,
and Supplementary Data S1-S7 can be found on Zenodo at
https://doi.org/10.5281/zenodo.15844033

Supplementary Results & Discussion

1. Genomic analysis of different LCA producing strains of *Gephyrocapsa huxleyi*

1.1 Nanopore sequencing

To evaluate the output of the Nanopore sequencing run we analyzed several parameters to assess the quality and continuity of a genome assembly, such as the maximum read length, the median read length and N50, a statistical measure that reflects the typical contig size emphasizing the contribution of longer contigs to the assembly. Median read length was approximately 1,300 base pairs (bp) in both strains, N50 was 4,322 bp and 6,576 bp, maximum read length was 241,259 bp and 134,905 bp for CCMP1742 and CCMP2758, respectively.

When we examined the sequencing output over time, we observed that for strain CCMP1742, 90% of the reads were obtained within the initial 15.6 hours, while for CCMP2758, this occurred within the first 11.4 hours. Additionally, half of the reads were acquired between 1.7 and 3.9 hours for both CCMP2758 and CCMP1742 strains, respectively.

Although the sequencing run lasted 72h, no values regarding read length and read quality are available after 30h for strain CCMP1742 and 21.5h for strain CCMP2758, which suggests that active sequencing effectively stopped long before the run was over. Analysis of the channel counts reveals that approximately 40% of the pores were inactive at the start of the sequencing run. Additionally, a significant portion of the pores became unavailable during the first 9h, resulting in a drastic drop in the number of active sequencing pores to only 2-10%. Although great care was taken to obtain clean high-quality unsheared DNA it is possible that the presence of polysaccharides, polyphenols and other phenolic compounds derived from the algal cell wall might have clogged the flow cell pores [1].

1.2 Comparison of genome assemblers

Only one genome assembly of *G. huxleyi* is currently available (strain CCMP1516). However, the existence of a *G. huxleyi* pangenome, in which genome sequences can vary largely across strains, with some strains sharing only 54-77% of their genome sequence with the reference strain CCMP1516 [2], complicates the use of this genomic assembly as reference for the genomic study of other *G. huxleyi* strains. In addition, the reference strain (CCMP1516), unlike strains CCMP1742 and CCMP2758, has not been reported to synthesize some of our compounds of interest such as the tetraunsaturated LCAs (e.g. $C_{37:4}$) nor short chain alkenones (C_{35}-C_{36}) (Table 1). Thus, to find the desaturase responsible for the addition of the fourth unsaturation and to identify the gene(s) responsible for the synthesis of C_{35}-C_{36} LCAs we sequenced and assembled the genome of *G. huxleyi* strains CCMP1742 and CCMP2758 before

proceeding with the analysis of gene expression of the RNAseq data. Given the large variety of genome assemblers available nowadays, each using a different approach to obtain contigs and scaffolds, their potential differences in their performance across different organisms [3], and the lack of a standardized method for obtaining good quality algal genomes [4] we tested different assemblers using either only long reads, only short reads, or a combination of long and short reads, and compared them to the CCMP1516 reference genome using the Quality Assessment Tool for Genome Assemblies (QUAST) [5] in order to select the most suitable tools to assemble our genomes of interest (Figure S2). QUAST calculates assembly quality measures like contig length distributions including N50 and identifies for example potential misassemblies (like inversions and translocations) and duplications based on the aligned closely-related reference genome provided.

Initially the genomes were sequenced using only long Nanopore reads and assembled using four different assemblers (Figure S2 and Data S2): CANU (v 1.8) [6], flye (v 2.8.1) [7], shasta (v 0.5.1) [8] and wtdbg2 (v 2.3) plus the consenser wtpoa-cns (v 2.3) [9]. For strain CCMP1742, flye, CANU, and wtdbg2 produced assemblies of 174.5 megabases (Mb), 126.6 Mb and 130.6 Mb, respectively, which are comparable to the 141.7 Mb of the CCMP1516 reference genome (Table S1). However, these assemblies contained a high number of potential misassemblies; 81%, 61% and 77%, of the total assemblies generated by flye, CANU and wtdbg2, corresponded to misassembled contigs according to QUAST. For strain CCMP2758 these assemblers produced much smaller assemblies of 95.4 Mb (flye), 42.19 Mb (CANU) and 20.46 Mb (wtdbg2) which were also largely misassembled, where 87%, 62% and 71% of the total assemblies´ length generated by flye, CANU and wtdbg2 corresponded to potentially misassembled contigs. Lastly, shasta generated unrealistically small assemblies for both strains: 8 Mb for CCMP1742 and 0.2 Mb for CCMP2758.

To improve the quality of the assemblies, we also sequenced our strains using short-read Illumina sequencing (Figure S2 and Data S2). We then evaluated various assembly methods including: a) using SPAdes to assemble only the short reads, b) hybrid genome assemblers (WenganD, WenganM, HASLR, OPERA-LG, SPAdes and SPAdesBio) and, c) scaffolders (OPERA-LG and WenganD) (Table S2). Assemblies obtained with WenganD and WenganM were ½ to ⅓ of the total length of assemblies obtained using hybridSPAdes. Assemblies obtained using HASLR were five to ten times larger than assemblies obtained with hybridSPAdes but contained a high estimated duplication ratio (4.4 for strain CCMP1742 and 6.6 for strain CCMP2758). Assemblies created using SPAdes with only short reads were similar to those obtained using the hybrid assemblers hybridSPAdes and BiosynthicSPAdes. Assemblies obtained using hybridSPAdes and scaffolded with OPERA-LG showed larger total assembly length (~236 Mb for CCMP1742 and ~204 Mb for CCMP2758), longer largest contig (~4.4 Mb for CCMP1742 and CCMP2758), and a lower percentage of potentially misassembled contigs (20–24% CCMP1742 and

33–39% CCMP2758). When WenganD was used as scaffolder, the percentage of potentially misassembled contigs increased considerably (~34%), resulting in up to 64% of the contigs being misassembled. However, when OPERA-LG was used as scaffolder, the percentage of potentially misassembled contigs only increased slightly (~6%) whilst the contiguity of the assemblies increased considerably, as shown by the increase of NG50 and the decrease of LG50 (Table S2). Therefore, we chose hybridSPAdes and biosyntheticSPAdes for assembly, and OPERA-LG for scaffolding to proceed with downstream analyses.

1.3 Genomic assembly and removal of prokaryotic contamination

Strains CCMP1742 and CCMP2758 are not axenic, meaning they contain prokaryotic contamination. Despite efforts to remove prokaryotes using different antibiotic treatments prior to sequencing, archaeal and bacterial contamination could not be completely removed (see BUSCO results in Table S2). Therefore, our original genomic dataset included reads from both *G. huxleyi* and the contaminating prokaryotes, primarily from bacteria.

To maximize the retention of genomic information from *G. huxleyi* while minimizing the presence of reads originating from contaminant prokaryotes (Figure 3, Supplementary Information, Figure S3, Tables S3, S4, and Data S3), we first assembled our reads as a metagenome (using metaSPAdes and OPERA-MS), and then assessed the taxonomy of each contig using CAT. The contigs that were annotated by CAT as non-eukaryotic had distinct GC content and coverage from the contigs that were annotated as eukaryotic (Figure S5), and thus reflect likely contamination. We selected only the reads mapping to contigs that were identified as eukaryotic or had no taxonomy assigned, reassembled them using SPAdes (hybridSPAdes and biosynthheticSPAdes) and created scaffolds with OPERA-LG (Figure S5). We then assessed the taxonomy of the resulting scaffolds with CAT and removed those identified as non-eukaryotic, keeping the contigs that had no taxonomy assigned as they had a GC content and coverage profile consistent with the eukaryotic contigs (Figure S5). Lastly, we evaluated the resulting assemblies using CAT, Benchmarking Universal Single-Copy Orthologs (BUSCO) and QUAST.

Taxonomic annotation of the metagenomic contigs generated by hybrid metaSPAdes revealed that 43% of the Illumina reads and 54% of the Nanopore reads from CCMP1742 were likely contaminants and were removed (Table S3). Remarkably, when using OPERA-MS as the metagenomic assembler, 99.7% of Nanopore reads were considered likely contamination (Table S3), potentially reflecting a high fraction of chimeric sequences in this assembly. For CCMP2758, 11% of the Illumina reads and 38% for Nanopore reads were identified as belonging to contaminants in the assembly generated by hybrid metaSPAdes (Table S3).

Upon removal of non-eukaryotic reads assemblies generated using hybridSPAdes contained approximately 20% less contigs than those assembled using biosyntheticSPAdes. After scaffolding using OPERA-LG, 19-25% of the scaffolds in the assemblies had a CAT taxonomic classification assigned, and 0.3-1% of these scaffolds were identified as belonging to non-eukaryotic organisms and were removed from the assemblies (Table S3).

Upon removal of reads and scaffolds identified as contaminants, all the resulting assemblies showed similar CAT and BUSCO results. CAT classification results remained similar to before removal of the contaminant scaffolds, and again only 19-25% of the scaffolds had a taxonomic annotation (Table S3), and the assemblies now contained ~68% of eukaryotic and 49% of bacterial BUSCO genes (Table S4). These results could suggest a high level of contamination in our assembly, however they are consistent with those obtained when analyzing the CCMP1516 reference genome, which has 73% and 49% of eukaryotic and bacterial BUSCO genes, respectively (Table S4). Although the absence of 25%-32% of eukaryotic BUSCO genes could suggest incompleteness of the assemblies, a 2020 study evaluating the quality of different algal genomes using BUSCO showed comparable results for other algal genomes [10]. In this study, out of the 222 algal genomes available at the time 78 were selected for further evaluation based on an N50 > 65 Kilobases. The authors observed that 68.6% of the eukaryotic BUSCO genes were present in 75% of the algal genomes of the dataset and that 34 BUSCO genes were found in less than 50% of the algal assemblies. Given the large number of genomes tested the authors inferred that it was unlikely that these missing BUSCO genes are conserved in all algal species and that quality metrics using BUSCO may be underestimating algal genome quality [10]. In addition, the presence of bacterial BUSCO genes could be an indication of incomplete decontamination both in our case and in the CCMP1516 reference genome, where bacterial contamination was minimized physically by adding kanamycin to the algal growth media and computationally by filtering out the bacterial scaffolds of the resulting assembly using megaBLAST [2].

The total length of the assembly varies depending on the genome assembler used, regardless of the metagenomic assembler used during the first step. For example, for strain CCMP1742, the assembly using hybridSPAdes resulted in assemblies of 161-165Mb, while using biosyntheticSPAdes yielded assemblies of 130-131 megabases (Mb). For strain CCMP2758, hybridSPAdes produced assemblies of 166Mb, whereas biosyntheticSPAdes generated assemblies of 132-133 Mb. As a reference, the assembly of CCMP1516 is 167 Mb [2] (Table S5). When contigs smaller than 500 bp were not included in the total length counts, the assembly size was reduced 28-41% depending on the assemblers used (Table S4).

Assemblies obtained using a combination of metaSPAdes and hybridSPADES had the highest total lengths, largest scaffolds and least fragmentation, as indicated by higher N50 and lower L50 values.

Therefore, we selected these assemblies and functionally annotated them with Funannotate. Additionally, to identify biosynthetic gene clusters (BGCs) that might not be completely assembled by hybridSPAdes, we also annotated the assemblies obtained using biosyntheticSPAdes despite their smaller assembly sizes.

Besides the main assembly file, biosyntheticSPAdes also generates a file containing putative BGCs, which may originally be dispersed across several contigs in the main assembly. A total of 39 BGC clusters were identified in CCMP1742, while 31 were identified in CCMP2758. Annotation of this file alone was not possible due to the relatively small number of genes it contained. To avoid missing any potential BGCs which could be responsible for LCA biosynthesis, we concatenated the main assembly and the BGC clusters prior to annotation with Funannotate (Figure 3). Since this approach could result in artificial gene duplications, we also evaluated the presence of multiple copies of BGC genes in our concatenated assembly. We identified five genes in each concatenated assembly that were duplicated due to this approach (Table S6). Additionally, we also observed that some genes (four in CCMP1742 and three in CCMP2758) were already duplicated within the file containing the putative BGCs (Tables S7 and S8).

1.4 Comparison of assembled genomes

Comparison of the annotations obtained using Funannotate (Table S5) showed that assemblies from biosyntheticSPAdes—despite being smaller in size—contain more predicted genes and proteins than the ones assembled using hybridSPAdes. When compared to the CCMP1516 reference genome assembly, all our assemblies contain more annotated genes and proteins, although the proportion of unique proteins is smaller than in CCMP1516.

When analysing the similarity between assemblies using OrthoVenn, we found that assemblies for both strains obtained from hybridSPAdes and biosyntheticSPAdes are highly similar, sharing approximately 86% of their proteins (Figure S6). Additionally, the assemblies of CCMP1742 and CCMP2758 shared 81-87% of their proteins with each other. We also observed that, regardless of the assembler used, CCMP1742 and CCMP2758 share nearly half of their encoded proteins with CCMP1516, which, given the smaller number of proteins in CCMP1516, corresponds to approximately 74% of its annotated proteins. However, it is important to note that the CCMP1516 reference genome was annotated using a different pipeline than that applied to CCMP1742 and CCMP2758 in this study.

Given that assemblies for both strains obtained from hybridSPAdes and biosyntheticSPAdes are highly similar, and that some of our genes of interest might be encoded within a BGC, in our subsequent analyses we will only focus on the assemblies obtained using biosyntheticSPAdes.

2. Identification of candidate genes involved in LCA biosynthesis

2.1 Identification of candidate genes involved in LCA formation

To date, two pathways have been proposed for LCA biosynthesis. Both pathways resemble fatty acid biosynthesis and propose that LCAs are synthesized as fully saturated chains to which double bonds are subsequently introduced by the action of at least four position-specific desaturases [11, 12], and that $C_{35\text{-}36}$ LCAs are obtained from longer LCAs via a chain shortening step. Given the structures of all LCAs identified to date (Table S9) and that monounsaturated LCAs have only been detected in a few selected strains and only in trace amounts (≤1.1% of total LCAs) [13], a pathway where LCAs are synthesized directly in their di-unsaturated form seems also possible.

We hypothesize the involvement of Polyketide Synthases (PKSs), which is a large family of multi-domain enzymes involved in the synthesis of polyketides (secondary metabolites) via a pathway with high similarities to fatty acid biosynthesis, but with more structural flexibility that would allow the biosynthesis of compounds with different carbon lengths, and containing double bonds and a keto group, such as the ones found in LCAs (see main text for further details). Thus, we propose an alternative LCA biosynthetic pathway in which the first two desaturations are not added by desaturases after the formation of the LCA backbone is completed, but during the elongation process itself, where only the third and fourth double bond require desaturase activity and whose synthesis mechanism does not require a chain shortening step to produce $C_{35\text{-}36}$ LCAs.

On basis of the proposed role of PKSs in the synthesis of LCAs, $C_{37:2}$ MeK $\Delta^{14,21}$ LCAs could, for example, be synthesized by an assembly line PKS composed of up to 17 modules each performing only one elongation round. Most modules would contain KS, DH, ER and KR subunits, except two modules that would lack the ER subunit, which would give rise to the double bonds found in LCAs and LCEs. Starting from Acetyl-CoA the process would involve 6 elongation steps using Malonyl-CoA, catalyzed by a PKS module containing all necessary subunits (e.g., KS, AT, KR, DH, ER, and ACP), followed by an elongation step with Malonyl-CoA carried out by a PKS module lacking the ER domain (Figure 4a, Figure S8). The absence of the ER domain in this step would prevent the reduction of the double bond, resulting in the Δ^{21} unsaturation present in the final LCA molecule. Next steps would involve elongation with Malonyl-CoA and an extender unit providing an odd number of carbons to the growing acyl chain (for example Succinyl-CoA or Propionyl-CoA) carried out by a PKS module containing all subunits, followed by an elongation step with Malonyl-CoA carried out by a PKS module lacking ER and where the double bound is again not reduced, resulting in the Δ^{14} unsaturation present in the final LCA molecule. Then after six additional elongation steps with Malonyl-CoA followed by decarboxylation, $C_{37:2}$ MeK $\Delta^{14,21}$ would be formed. The modular nature of PKSs may explain the diversity of observed LCAs. If for example, instead

of six elongation steps with Malonyl-CoA there were five elongation steps with Malonyl-CoA followed by one last elongation step with Methylmalonyl-CoA before decarboxylation, the resulting molecule would be $C_{38:2}$ EtK $\Delta^{14,21}$. If the exact same pathway was followed but instead of Acetyl-CoA the starting molecule was Propionyl-CoA (Figure 4b, Figure S9), $C_{38:2}$ Me $\Delta^{14,21}$ would be obtained if the last elongation step was carried out using Malonyl-CoA and $C_{39:2}$ Et $\Delta^{14,21}$ would be formed if the last elongation step was carried out using Methylmalonyl-CoA.

This proposed pathway would explain the synthesis of the most common LCAs, such as $C_{37:2}$ MeK $\Delta^{14,21}$, $C_{38:2}$ EtK $\Delta^{14,21}$, $C_{38:2}$ MeK $\Delta^{14,21}$ and $C_{39:2}$ EtK $\Delta^{14,21}$. However, it is known that the modules of assembly line PKSs can sometimes be used repeatedly (module stuttering) or not used at all (module skipping) [14–16]. Thus the repeated use or skipping of some of the modules could also explain the shorter lengths and different positioning of the two double bonds in some LCAs such as in $C_{35:2}$ MeK, $C_{36:2}$ MeK and $C_{36:2}$ EtK LCAs found in CCMP2758, where the double bonds occur at positions $\Delta^{12,19}$—two carbon atoms closer to the carbonyl end than in the usual $\Delta^{14,21}$ LCAs [12, 17]. Occasional module-skipping during synthesis of $C_{37:2}$ MeK and $C_{38:2}$ EtK, could also explain the presence of trace quantities (≤1.1% of total LCAs) of $C_{35:1}$ MeK and $C_{36:1}$ EtK found in some haptophyte strains (Table S9) [13, 14]. For example, this might occur if the growing acyl chain is prematurely transferred to the next module, skipping the module lacking the ER, resulting in a shorter molecule with only one double bond (Figures S8 and S9) Likewise, module stuttering could explain the positions of the double bonds in $C_{35:2}$ MeK, $C_{37:2}$ MeK and $C_{38:2}$ EtK LCAs found in the Black Sea and in hypersaline and marine costal microbial mats, which contain double bonds separated by three methylene groups—instead of the usual five methylene groups—at positions $\Delta^{14,19}$ [18–21].

PKSs are known for the wide structural diversity of the products they can synthesize. One of the ways they achieve this, is by using different combinations of a variety of starter and extender units. Acetyl-CoA and Propionyl-CoA are the most common starter units in PKSs, and other molecules like for example Succinyl-CoA, Isobutyryl-CoA, Benzoyl-CoA and 4-aminobutanoyl-CoA can also be used as starter units [22, 23]. Additionally, although Malonyl-CoA and Methylmalonyl-CoA are the most common extender units for PKSs, other molecules have also been found to be used as extender units, such as Ethylmalonyl-CoA, Chloroethylmalonyl-CoA, Allylmalonyl-CoA, Benzylmalonyl-CoA and 4-Oxoadipyl-CoA [24]. These extender molecules usually add two carbons to the growing acyl chain, while the rest of the molecule is incorporated as a side chain, e.g. when a Methylmalonyl-CoA extender unit is added, the acyl chain is extended by two carbons, and a branch with one methyl group is created. However, in order to have two double bonds separated by an odd number of carbons—as is the case in LCAs—one of the extender units added to the growing acyl chain between the addition of the first and second double bond should be an odd-numbered molecule. Even though to our

knowledge there are no reports yet of an extender unit capable of adding three carbon atoms to the main chain, such as implied by our proposed biosynthetic mechanism described above, a wide variety of starter and extender molecules has been discovered in the last couple of decades. Given the biosynthetic flexibility of PKSs, it is thus likely that novel extender units can add and odd number of carbons to the growing acyl chain (for example molecules like Succinyl-CoA or Propionyl-CoA).

To summarize, based on the LCA structures detected in haptophyte cultures to date and on the biosynthetic pathway mentioned above we hypothesize that LCA biosynthesis may start from the condensation of an Propionyl-CoA or Acetyl-CoA primer molecule to a Malonyl-CoA extender unit, followed by several elongation rounds with Malonyl-CoA, that MeKs result from the use of a Malonyl-CoA extender unit during the last extension of the alkenoyl-ACP molecule, and that EtKs result from the use of a Methylmalonyl-CoA extender unit during the last extension. Additionally, the stuttering or skipping of some modules could explain differences in chain length and double bond positioning found across LCA structures.

2.1.1 PKS7 as candidate for LCA elongation in CCMP1516 based on its structure

In the CCMP1516 reference genome annotation, we identified 53 genes based on functional annotation that could be involved in LCA biosynthesis, of which 12 are assembly line PKSs. PKSs are common in algae, and *G. huxleyi* CCMP1516 is one of the species with the highest number of annotated assembly line PKSs [25]. The number of candidate genes identified when the genome had been annotated using Funannotate was much higher than in the reference genome annotation (Table 2, Table S5). This again underscores the challenge of comparing genomes annotated using different annotation pipelines.

Upon manual inspection of the CCMP1516 reference genome we found a promising assembly-line PKS for LCA formation, based on its domain structure. PKS7 is a large protein composed of 21,165 aminoacids (aa) and arranged in 13 modules, some possessing identical DNA sequences. Most modules contained ER, KR, ACP, KS and DH domains (Figure S7). However, two of the modules lacked the ER domain, which catalyzes the reduction of the α,β-unsaturated bond to a saturated bond. Thus, when this domain is missing the reduction cycle is not completed, leaving a double bond (usually in *trans*-configuration) in the growing carbon chain [26, 27]. Assuming that each module carries out only one extension cycle using malonyl-CoA as an extender unit, we predict that PKS7 would produce a C_{30} compound resembling an LCA with double bonds at $\Delta^{10,16}$ (Figure S7a). However, as previously mentioned, the modules of assembly line PKSs can sometimes be used repeatedly (module stuttering) or not used at all (module skipping) [14–16]. Thus, taking this into account, we hypothesize that PKS7 could synthesize $C_{37:2}$ Me following these steps: 1) using Propionyl-CoA as starter unit, 2) followed by four elongation rounds using Malonyl-CoA carried out by four complete modules (i.e. modules

containing ER, KR, ACP, KS and DH), 3) two full elongation rounds carried out by the same module (module stuttering), 4) elongation round carried out by a module using Malonyl-CoA as extender unit and lacking ER (which would add the first unsaturation at Δ^{21}), 5) one full elongation using Malonyl-CoA, followed by 6) full elongation round using an extender unit providing three carbons, and 7) another elongation round carried out by a module using Malonyl-CoA as extender unit and lacking ER (which would add the second unsaturation at Δ^{14}), followed by 7) six elongation rounds caried out by three complete modules using Malonyl-CoA (in which at least one module would "stutter" and carry out the full elongation round more than once) , and 8) molecule release carried out by the TE domain (Figure S7b). While the general composition of the final molecule can be anticipated [14] it is not yet possible to accurately determine its exact final structure based solely on PKS architecture [26, 28]. Thus, the reactions and final products of PKS7 mentioned are tentative and would require further experimental validation to confirm or refute them.

Additionally, given the modularity of the sequence and their repetitive structure, we evaluated sequence similarities within PKS7 and observed that some modules possessed identical DNA sequences, suggesting that they likely arose from a duplication event within the PKS. The presence of repetitive sequences can cause issues during gene annotation [29, 30]. On one hand, these sequences can lead to the misidentification of coding regions, resulting in non-coding sequences being mistakenly labelled as genes (false positives). On the other hand, genes containing repetitive sequences may be overlooked entirely (false negatives) or fragmented into multiple separate genes, leading to incomplete or inaccurate gene annotations. This, together with differences in the methods used for gene prediction within the Funannotate pipeline and the unusual large size of assembly line PKSs, could explain the higher number of genes (21 instead of one) predicted to be homologous to PKS7 when using CCMP1516 Funannotate's genome annotation. Furthermore, the low contiguity of the CCMP1742 and CCMP2758 genomes may also contribute to the large number of hits obtained for these two strains due to fragmentation of a gene in multiple contigs.

Given the domain architecture suggesting PKS7's involvement in LCA formation, and to narrow down the number of candidate genes in the other two strains, we searched for homologs of PKS7 using BlastP against CCMP1742 and CCMP2758 genomes assembled using biosyntheticSPAdes. We identified 93 hits for CCMP1742 and 95 hits for CCMP2758. Most proteins homologous to PKS7 in CCMP1742 and CCMP2758 were relatively small in comparison with PKS7 (21,165 aa), potentially reflecting difficulties in gene prediction and fragmentation of this very large gene. Of these PKS7 homologous proteins only 3 in strain CCMP1742 were larger than 2,000 aa, whereas strain CCMP2758 contained at least five proteins larger than 10,000 aa that were homologous to all PKS7's ACP, KS, KR and ER domains, and to 11 out of its 12 DH domains (Table S10).

2.2 Identification of candidate desaturases

Analysis of all LCA structures previously reported in literature (Table S9) indicates that in most tri-unsaturated LCAs the third double bond is located at the Δ^7 position, and the fourth double bond at the Δ^{28} (Figure 1). However, the identification of $\Delta^{14,21,28}$ tri-unsaturated and $\Delta^{7,14,19,28}$ tetra-unsaturated LCAs in Greenlandic lakes suggests that this order can be reversed [31]. Since our LCA analysis cannot determine the position of the double bonds, here we assume that the third unsaturation is found at the Δ^7 position, and the fourth unsaturation at the Δ^{28}.

Fatty acid desaturases can be classified into several groups based on their selectivity and the direction in which they add these double bonds: i) Δ-desaturases measure their distance from the carboxyl end, ii) ω-desaturases measure their distance from the methyl end of the alkyl chain, iii) methyl-end desaturases recognize an existing double bond and insert the next one towards the methyl end, iv) front-end desaturases recognize an existing double bond and insert the next one towards the carboxyl end [32]. While LCAs and fatty acids are structurally similar, LCAs contain a carbonyl group rather than a carboxyl group. It is likely that LCAs may be modified by enzymes analogous to fatty acid desaturases using similar catalytic mechanisms but adapted to target the carbonyl group instead of the carboxyl group. Since the distance between the keto group and the third unsaturation remains constant amongst LCA structures (i.e. Δ^7) [11] it is likely that the desaturase responsible for the addition of this double bond is a Δ-desaturase [11] acting at a fixed distance from the carbonyl group. Additionally, the position of the fourth unsaturation—often found almost exclusively at Δ^{28} closest to the methyl end of the molecule and separated by 5 methylene units from the Δ^{21} double bond (Figure 1, Table S9)—initially indicates that the enzyme responsible for this unsaturation is either a Δ-desaturase acting at a distance of 28 carbons from the carbonyl group, or a methyl-end desaturase acting seven carbon atoms away from the first double bond (Δ^{21}) and towards the methyl end of the molecule (Figure 1 and Table S9) [32]. However, the absence of a fourth double bond in the shorter C_{35-36} $\Delta^{7,12,19}$ LCAs [12], suggests that the enzyme responsible is likely a Δ^{28} desaturase, which requires the other three double bonds to be positioned at Δ^7, Δ^{14} and Δ^{21} to catalyze the reaction.

AKD1 is the desaturase responsible for the conversion of $C_{37:2}$ to $C_{37:3}$ LCAs in the haptophyte *T. lutea* [33], and a closely-related gene may be involved in the same reaction in *G. huxleyi*. To identify homologs of AKD1 in *G. huxleyi*, we performed a BlastP search to the nr database (accessed on 10th August 2023) limiting the search to "*Emiliania huxleyi* CCMP1516 (taxid:280463)" [2, 34, 35], and selected hits with at least 50% query coverage, 30% identity and bitscore > 150 for further analysis, namely: EMIHUDRAFT_196284, EMIHUDRAFT_454486, EMIHUDRAFT_460273, EMIHUDRAFT_96351 and EMIHUDRAFT_444795 (Table S12). The best hit to ADK1 was EMIHUDRAFT_196284, a "putative fatty acid desaturase" based on its annotation, covering 89% of the AKD1 sequence and sharing 48%

of its bases. All other hits had no annotated function according to the reference annotation, but were all described by Uniprot as "Fatty acid desaturase domain-containing protein" (Table S12), except EMIHUDRAFT_454486, which was described as an "uncharacterized protein". EMIHUDRAFT_196284, EMIHUDRAFT_444795, EMIHUDRAFT_460273 and EMIHUDRAFT_96351 have been previously identified as "orphan" desaturases (i.e. sequences with homology to other desaturases already characterized, but that are not annotated and whose substrate is unknown) and were considered as potential candidates for catalyzing the addition of the third double bond in the LCAs of *G. huxleyi* by the authors responsible for the discovery of AKD1 [33]. In addition to these four genes, we thus also included EMIHUDRAFT_454486 as a potential candidate gene for carrying out this reaction based on its sequence homology and high alignment score to AKD1. To identify which genes are responsible for the addition of the third unsaturation in CCMP1742 and CCMP2758, we used OrthoFinder within the OrthoVenn3 platform [36, 37] to search for orthologs of these CCMP1516 "candidate desaturases" in the BGC assemblies of CCMP1742 and CCMP2758. However, since CCMP1742 and CCMP2758 were annotated using a different annotation pipeline than the reference genome in which these "orphan" desaturases had been identified, and to evaluate the effect of using a different annotation pipelines in our analysis—i.e. differences in missing or unannotated genes—we also searched for orthologs of these desaturases in the CCMP1516 and *T. lutea* genomes annotated with Funannotate (Table S13). We identified two orthologous desaturases in the Funannotate *T. lutea* genome annotation, 4 and 6 desaturases (some with multiple potential isoforms) in the genomes of strains CCMP1742 and CCMP2758, respectively and 4 in the CCMP1516 genome assembly annotated with Funannotate (Table S13). Additionally, whilst orthologs of most candidate desaturases were also found when CCMP1516 was annotated using Funannotate, no ortholog for EMIHUDRAFT_454486 was found when using this annotation pipeline, suggesting that this gene might not have been properly predicted during genome annotation with Funannotate. This underscores again the challenge of comparing genomes annotated with different annotation pipelines.

3. Evaluating candidate genes through culturing: monitoring LCA profiles and gene expression

3.1 Effect of cold shock on CCMP1516 gene expression

To identify differences in gene expression between the control and cold-shocked CCMP1516 cultures, we first performed principal component analysis (PCA) on variance stabilizing transformed (vst) normalized gene counts. This showed grouping of the control versus the cold-shocked cultures but also one outlier (one of the transferred cultures obtained after 3 h; Figure S11a). This outlier was further confirmed with Cook's distance Figure S12a [38]. Therefore, we excluded this outlier and repeated the PCA and Cook's distance analysis (Figure S11b and S12b). This again showed a clear separation on the

first principal component (PC1, describing 71% of the variance) between the control and the cold-shocked cultures (Figure S11b). The time of sampling had a relatively small effect (PC2, 8% variance) on gene expression. (Figure S11b). Differential gene expression analysis revealed that the cold shock resulted in a total of 48 up-regulated genes and 157 down-regulated genes (≥1 and ≤-1 Log2 Fold Change (LFC), p-value <0.01).

We examined the expression of the five *G. huxleyi* CCMP1516 candidate desaturases described in section 2.2 above during a cold shock experiment (Figure S13). Most *G. huxleyi* CCMP1516 candidate desaturases exhibited low transcript counts (<10 reads per sample, Figure S14), likely representing background noise, and showed no significant differential expression across temperatures (Table S14). Only EMIHUDRAFT_454486, a hypothetical protein where 90% of its sequence aligns with AKD1 and which shares 42% of its bases, was significantly overexpressed when grown at 10°C (Tables S12 and S14).

3.2 Effect of cold shock on CCMP1742 and CCMP2758 cell abundances

To elucidate which gene(s) is responsible for the addition of the fourth unsaturation in LCAs, we carried out experiments using *G. huxleyi* strains isolated from higher latitudes and known to synthesize tetra-unsaturated LCAs when grown at temperatures below 15°C, i.e., strains CCMP1742 and CCMP2758 [17, 39]. In cold shock experiment carried out with strain CCMP2758 cultures were grown at 15°C, then cold shocked by transferring half of the cultures to 3°C and monitored for 12h. The cell density of cultures kept at 15°C remained stable at 3.0 x 10^6 cells ml^{-1} throughout the experiment (Figure 6a), whilst cell density of cultures transferred to 3°C increased slightly up to 4.3 x 10^6 cells ml^{-1}. No significant changes (p_{adj} > 0.05, Data S1, S4 and S5) in $C_{37:4}$ per cell, nor in the $C_{37:3}$ / $C_{37:2}$ ratio were observed in the cultures transferred to 3°C after 12 h (Figure 6a). Thus, in the next cold shock experiment carried out with strain CCMP1742, a strain closely related to CCMP2758 that also synthesizes $C_{37:4}$, we extended the cold shock exposure time to 96 h. The cell density of cultures kept at 15°C increased slightly from 1.5 x 10^6 cells ml^{-1} at the start of the experiment, to 1.9 x 10^6 cells ml^{-1} after 96h (Figure 6b). However, during the first 12h of the cold shock, cell density in the flasks transferred to 3°C increased to 2 x 10^6 cells ml^{-1}, but then rapidly decreased, reaching 3 x 10^5 cells ml^{-1} after 96h. After 96h, the concentration of $C_{37:2}$, $C_{37:3}$ and $C_{37:4}$ LCAs per cell had increased in flasks transferred to 3°C—a change that might have resulted from the overestimation of their concentrations per cell due to the decrease in cell concentrations coupled with the collection of all cellular debris during filtration for LCA quantification, rather than to an increase in LCA biosynthesis (Figure S15). Since the cultures in all flasks originated from the same culture—and thus contained approximately the same number of cells—we evaluated the concentration of $C_{37:2}$, $C_{37:3}$ and $C_{37:4}$ LCAs per volume of culture filtered instead of per cell (Figure S16f) to see whether new LCAs were synthesized. Although

growth at colder temperatures usually promotes the synthesis of LCAs and triggers the desaturation of $C_{37:2}$ to $C_{37:3}$, here we observed similar concentrations of $C_{37:2}$, and higher concentrations of $C_{37:3}$ and $C_{37:4}$ LCAs per mL, in cultures kept at 15°C. Thus, strengthening our hypothesis that the increase in $C_{37:2}$, $C_{37:3}$ and $C_{37:4}$ LCAs per cell is likely an artifact caused by the overestimation of cell counts in cultures kept at 3°C.

3.3 Impact of temperature acclimation on LCA formation and desaturation in strains CCMP1742 and CCMP2758

3.3.1 The effect of temperature on cell growth

Since the cold shock experiments using *G. huxleyi* strains CCMP1742 and CCMP2758 did not result in an increased proportion of $C_{37:3}$ and $C_{37:4}$ LCAs as expected, we took a different approach and proceeded to acclimate the cultures to 20°C and 7°C and analyze them at different growth stages. Prior to the experiment, the strains had been kept at 20°C and 7°C over a period of several weeks for them to adapt to their new cultivation temperatures. At the start of the experiment cultures were inoculated at a cell density of 1.0 x 10^5 cells ml^{-1}. Overall, both strains followed a similar growth pattern over time. It took 12 days for *G. huxleyi* CCMP1742 grown at 20°C to reach their maximum cell density (4.3 x 10^6 cells ml^{-1}), whilst cells grown at 7°C only reached maximum cell density (2.9 x 10^6 cells ml^{-1}) after 31 days (Figure S16). Cultures of *G. huxleyi* CCMP2758 grown at 20°C reached their maximum cell density (5.0 x 10^6 cells ml^{-1}) after 13 days, whilst cells grown at 7°C only reached maximum cell density (2.8 x 10^6 cells ml^{-1}) after 32 days (Figure S16).

Cell abundances were monitored by measuring chlorophyll absorbance using flow cytometry. Two clusters with variable chlorophyll absorbances appeared in all samples; however, toward the end of the growth curve of strains grown at 20°C there was an increase in the number of cells appearing in the cluster with lower chlorophyll values. This could potentially show the metabolic state of the cell and indicate a decay in cellular and photosynthetic activity.

3.3.2 LCA and LCE peaks identification using mass spectrometry

After quantification of all peaks present in the ketone fraction, we proceeded to determine their structure using mass spectrometry (Data S1). The exact position of the double bonds could not be determined from our results, and their position was assigned based the retention times and elution order extracted from previous studies using the same GC column (Restek RTX-200 column) [40]. Although in most LCA structures the third unsaturation is located between the second unsaturation and the ketone group (Figure 1 and Figure S1), in some cases the third unsaturation was located between the first unsaturation and the methyl end of the molecule (Table S9). To distinguish between both isomeric forms, we will use the notation described in [41]. Briefly, the term isomer "a" will be

used when referring to those compounds for which the third double bond is located proximal to the ketone moiety ($\Delta^{7,12,19}$ for $C_{35\text{-}36}$ LCAs and C_{34} LCEs and $\Delta^{7,14,21}$ for $C_{37\text{-}40}$ LCAs and C_{36} LCEs) and isomer "b" when the third unsaturation is located closer the terminal methyl group ($\Delta^{14,21,28}$ for $C_{37\text{-}40}$ LCAs and C_{36} LCEs) [12, 21, 40]

The ketone fractions of strain CCMP1742 grown at 7°C (Figure 2 and Table S16) contained up to 26 different compounds, comprising mostly LCEs and LCAs and one unidentified compound (eluting between $C_{38:3}$EtK and $C_{38:2}$MeK and named "Unidentified 2"). These include di-, tri- and tetra-unsaturated C_{37}MeK, C_{38}EtK, C_{38}MeK, C_{39}EtK, small amounts of tri- and di- unsaturated C_{39}MeK and C_{40}EtK LCAs, as well as di-, tri- and tetra- unsaturated C_{36}OMe and C_{36}OEt LCEs. Most of the compounds containing three double bonds also contained a smaller adjacent peak. We identified the peaks adjacent to $C_{36:3}$OMe and $C_{36:3}$OEt as potential $C_{36:3}$OMe and $C_{36:3}$OEt isomers based on their distinct $[M]^{+\cdot}$ ions (m/z 544 and m/z 558) and $[M-31 \text{ or } M-45]^+$ (m/z 513) obtained from cleavage of the methyl and ethyl ester group (Table S17). The spectrum of the peak immediately after $C_{38:3}$EtK features a distinct $[M]^{+\cdot}$ ion (m/z 542) and $[M-29]^+$ (m/z 513), indicating that it is likely an isomer of $C_{38:3}$EtK (Table S17). No mass spectra could be obtained from the peak adjacent to $C_{37:3}$MeK, nonetheless it is likely that this smaller peak corresponds to a $C_{37:3}$MeK isomer. Since the unsaturation positions could not be determined from the mass spectra, we compared the order of elution with previous literature reports using the same GC column [40] and determined that the first peak to elute likely corresponds to isomer "a" and the peak eluting immediately after to isomer "b". Whilst for $C_{36:3}$OMe, $C_{37:3}$MeK and $C_{38:3}$EtK isomer "a" is more abundant, isomer "b" is the most abundant one for $C_{36:3}$OEt. When the same strain was grown at 20°C, the overall concentrations of tri- and tetra- unsaturated compounds decreased as expected (Table S16, Figure S17). No tri- and tetra- unsaturated LCEs were detected in these samples, and tetra-unsaturated and "b" isomers of tri-unsaturated $C_{37\text{-}39}$-Me and -Et LCAs were either not present or present in trace amounts (< 0.1 fg $cell^{-1}$).

The ketone fractions of strain CCMP2758 grown at 7°C (Figure 2 and Table S16) contained up to 37 different compounds. These include all the LCAs detected in CCMP1742 grown at 7° C, some of the LCEs and an additional unidentified compound. As previously reported in [12], in addition to the common LCAs synthesized by CCMP1742, CCMP2758 produces a set of shorter di- and tri- unsaturated LCAs, namely: C_{35}MeK, C_{36}EtK and C_{36}MeK. When samples were run on a GC-FID (Figure 2) we could distinguish two consecutive peaks eluting immediately before the peak identified as $C_{36:2}$EtK. However, when the same sample was analyzed using gas chromatography coupled to mass spectrometry (GC-MS) only one peak was observed. This peak showed a distinct $[M]^{+\cdot}$ ion (m/z 514) and a $[M-29]^+$ fragment (m/z 485), characteristic from the cleavage of the ethyl group in LCAs $[M-CH_2\text{-}CH_3]^+$, hence it was identified as $C_{36:3}$EtK (Table S17).

Additionally, we observed two unidentified peaks between $C_{36:2}$MeK and $C_{37:4}$MeK. the first peak showed two distinct $[M]^{+\cdot}$ ions, m/z 558 and m/z 528, and two additional fragments of mass m/z 513 and m/z 499 (Figure S18a and Table S17). Based on these fragments, we tentatively identified the compounds found in this peak as a mixture of $C_{36:3}$OEt and $C_{37:3}$EtK. To disentangle the contents of the peak we carried out base hydrolysis, a treatment that removes all fatty acid esters from the sample whilst keeping LCAs intact. After base hydrolysis (Figure S18b), only the $[M]^{+\cdot}$ ion with m/z 528 and $[M-29]^{+}$ (m/z 499) could be observed, thus confirming its identification as $C_{37:3}$EtK. Therefore, since LCEs were removed during base hydrolysis and based on the $[M]^{+\cdot}$ ion (m/z 558) and $[M-45]^{+}$ (m/z 513) corresponding to the loss of an -OEt group, we identified the coeluting compound as $C_{36:3}$OEt.

Similarly, the peak eluting immediately after showed two distinct $[M]^{+\cdot}$ ions, m/z 560 and m/z 530, and two additional fragments with masses m/z 515 and m/z 501 (Figure S19a). Based on these fragments, we tentatively identified the compounds found in this peak as a mixture of $C_{36:2}$OEt and $C_{37:2}$EtK (Table S17). To confirm this, we compared the fragments found in this peak before and after base hydrolysis. After hydrolysis only the $[M]^{+\cdot}$ ion (m/z 560) and $[M-29]^{+}$ (m/z 515), corresponding to fragments characteristic for $C_{37:2}$EtK remained (Figure S19b). Hence, based on the $[M]^{+\cdot}$ ion (m/z 560) and $[M-45]^{+}$ (m/z 515), the coeluting compound was identified as $C_{36:2}$OEt.

Besides di- and tri-unsaturated $C_{36:}$Me LCEs—which were also present in strain CCMP1742—we identified four new short LCEs synthesized by CCMP2758: $C_{34:3}$OEt and $C_{34:2}$OEt featured a distinct $[M]^{+\cdot}$ ion (m/z 530 and 532, respectively) and $[M-45]^{+}$ fragment (m/z 485 and 487, respectively), caused by the cleavage of the ethyl ester group $[M-O\text{-}CH_2\text{-}CH_3]^{+}$ (Figure S20 and Table S17). Whilst $C_{34:3}$OMeK, $C_{34:2}$OMeK, $C_{34:3}$OEtK and $C_{34:3}$OEtK. $C_{34:3}$OMe and $C_{34:2}$OMe LCEs feature a distinct $[M]^{+\cdot}$ ion (m/z 516 and 518, respectively) and $[M-31]^{+}$ fragment (m/z 485 and 487, respectively), caused by the cleavage of the methyl ester group $[M-O\text{-}CH_3]^{+}$ (Figure S21 and Table S17). Additionally, we observed a peak eluting between $C_{34:2}$OEt and $C_{33:3}$MeK for which we could not obtain a clear mass spectrum and which we refer to as “Unidentified 1”.

When grown at 20°C the amount of di-unsaturated compounds increased in both strains (Figure S17), whilst the tri- and tetra-unsaturated compounds drastically decreased to trace ($C_{36:3}$EtK, $C_{36:3}$MeK, $C_{37:4}$MeK, $C_{37:3b}$MeK, $C_{38:4}$EtK, $C_{38:3b}$EtK) or undetectable amounts ($C_{38:4}$Me and $C_{39:4}$Et). At 20°C the overall amount of C_{39} LCAs was considerably reduced and no C_{40} LCAs could be detected. Whilst, di-unsaturated C_{34}OMe, C_{34}OEt, C_{36}OMe, tri-unsaturated C_{36}OMe and the tri-unsaturated compound mix of C_{36}OEt LCE and C_{37}EKt LCA could only be detected in trace amounts in some of the samples. At 20°C only the compound mixture containing di-unsaturated C_{36}OEt LCE and C_{37}EtK LCA was detected in all the samples (Table S16).

Overall, lipid analysis revealed the presence of LCAs previously described in the literature for these strains [12, 17], including; C_{37} MeK, C_{37} EtK, C_{38} MetK, C_{38} EtK, C_{39} MeK, C_{39} EtK and C_{40} EtK, and their structurally related LCEs; C_{36}OMe, C_{36}OEt in both strains (Figure 2, Table S16 and Figures S18 and S19), and of the shorter C_{35} MeK, C_{36} MeK, C_{36} EtK LCAs and C_{34}OEt LCE exclusively in strain CCMP2758 (Table S16 and Figures S20 and S21). While all these compounds were present in their di- and tri-unsaturated form when strains were grown at 7°C, we also detected C_{37}MeK, C_{38}MeK, C_{38}EtK and C_{39}EtK LCAs and, C_{36}OMe and C_{38}OEt LCEs in their tetra-unsaturated form (Figure 2, Table S16 and Figure S17). Additionally, here we report the identification of C_{34}OMe and C_{34}OEt LCEs in strain CCMP2758 which to the best of our knowledge had not been previously detected in this strain. (Tables S16 and S17, and Figures S20-22).

3.3.3 Changes in total LCA and LCE abundances over time

To evaluate differences in LCA and LCE biosynthesis at different growth stages, their abundances were monitored at six different time points according to their cell density and growth stage (Figure 7 and Figures S16 and 23). Given the slower growth observed when strains were grown at 7°C cultures were not always sampled after the same incubation time, therefore t1-t5 labels represent a different number of days depending on each strain and condition.

The total amount of LCAs and LCEs (LCAs+LCEs, hereafter referred to as "Long chain alkenones/esters" or abbreviated as LCAEs) in strain CCMP1742 followed the same trend at both temperatures; that is, the amount of LCAEs decreased from the early (t1) until the mid-exponential phase (t3) and then increased during the late exponential to stationary phase (t4-t5) (Figure 7c), reaching a maximum of 73.7 ± 2.7 fg $cell^{-1}$. When strain CCMP2758 was grown at 20°C, the amount of LCAEs decreased from t1 until t4, and increased slightly at t5 (Figure 7d), whereas when grown at 7°C the total amount of LCAs and LCEs per cell followed an increasing trend from t1 to t5, reaching a concentration 78.0 ± 19.1 fg $cell^{-1}$ at t4 and a maximum of 109.2 ± 4.8 fg $cell^{-1}$ at t5. Overall, both strains produced higher amounts of LCAEs per cell when grown at 7°C (Figure 7b,c and Figure S23). We then compared LCAE abundances over time across samples for which also transcriptomic data was available (7°C; t1, t2 and t3, and 20 °C; t1, t2 and t4, Figures S16, S23 and S30 and Table S15). Significant differences in the total LCAE concentration per cell over time at 20°C were only observed in CCMP1742 between t1 and t4 (Figure S23a, $p_{adj} \leq 0.01$) and in CCMP2758 between t1 and t4 (Figure S23c, $p_{adj} \leq 0.0001$), and between t1 and t2 ($p_{adj} \leq 0.0001$). No significant differences in LCAE abundances over time were observed when strains were grown at 7°C ($p_{adj} > 0.05$, Figure S23b,d).

3.3.4 Differences in LCA composition between CCMP1742 and CCMP2758

Strains CCMP1742 and CCMP2758 are closely related, yet they do not produce the same LCAs. Strain CCMP2758 originated spontaneously from a CCMP1742 laboratory culture that was routinely used in

experiments focused on LCA biosynthesis. After nine months—during which the culture was only maintained but not used for any experimental purpose—the authors of a previous study noticed the production of three new compounds identified as $C_{35:2}$ MeK, $C_{36:2}$ MeK and $C_{36:2}$ EtK LCAs [17]. The strain was deposited in the culture collection of the Provasoli-Guillard National Center for Culture of Marine Phytoplankton under its new strain identifier, CCMP2758. In subsequent experiments carried out by the same research group, the relative abundances of these new compounds varied greatly between experimental batches and could represent between 13% and 74% of the total LCAs depending on the culturing conditions [17]. There have been other reports of haptophytes with distinct changes in their LCA profile over time, such as the *T. lutea* strain commercialized under the name of *Phytobloom* by Necton, which in 2016 produced C_{37-39} LCAs, but in 2018 started to produce mostly C_{35-36} LCAs [42].

In addition to modifying their degree of unsaturation, lipids can also adapt to temperature changes by altering their acyl chain length [43, 44]. However, because of the differences in naming between LCAs and LCEs (i.e. LCAs are named according to the total number of carbons of the molecule (depicted as R in Figure 1) while LCEs are named according to the number of carbons in -R' (Figure 1), potential changes in chain length related to temperature or growth stage may not be readily apparent from the compound names alone. To evaluate potential changes in LCAE composition that could indicate underlying changes in the biosynthetic pathway induced by temperature or growth stage (i.e. an increase in the number of carbons in R' would indicate additional elongation rounds during biosynthesis), we analyzed the relative abundances of LCAEs within the same strain based on structural characteristics—such as total compound length (-R, Figure 1 and Table S18) and the number of carbons on either side of the keto group (-R' and -R'', Figure 1, Figure 8, Figures S17, S24, S26-S28, and Tables S18 and S19). We observed that—as expected— the number of double bonds was mostly influenced by growth temperature in both strains (Figures S17, S25, and Table S19). Overall, relative abundance of LCAs was higher than that of LCEs for both strains and growth conditions however relative abundances of LCEs increased when cultures were grown at 7 °C (Figure 8a, Figure S24, Table S19). The relative abundances of compounds with the same number of carbons in R, R' and R'' (Figure 1), remained stable across time and temperature within strains (Figure 8b-d, Figures S26–S28, and Tables S18 and S19). Compounds containing an even number of carbons in R' were produced in higher proportions than those containing an odd number of carbons (Figure 8c, Figure S27, Table S19). LCAEs containing methyl groups were overall more abundant (65.1% ± 3.9% in CCMP1742, and 71.0% ± 3.5% in CCMP2758, Figure 8e, Figure S28 and Table S19 panel i) than their ethyl counterparts (34.7 % ± 3.9% in CCMP1742, and 28.9% ± 3.4% in CCMP2758). This suggests, the neither the number of elongation rounds, nor the frequency of ethyl or methyl group addition is influenced neither by temperature nor by the growth stage of the culture, suggesting that the LCAE biosynthetic mechanism is stable.

Next, we compared these structural characteristics across strains. While compounds containing 37, 38 and 39 carbons in R constituted 51.0% ± 4.2%, 43.8% ± 3.3% and 4.9% ± 1.0% of all LCAEs in CCMP1742, respectively, these compounds were substantially lower in CCMP2758, where they represented 23.9% ± 4.3%, 20.9% ± 1.4% and 2.5% ± 0.3% of LCAEs respectively (Figure 8b, Figure S26, and Table S19c). We also observed that in CCMP2758 the relative abundances of compounds that contain 34 and 35 carbons in R' (42.1% ± 4.3%, and 8.4% ± 4.3% respectively) are similar to the abundances of compounds that contain 36 and 37 carbons in R' (38.0% ± 3.6%, and 9.0% ± 1.5% respectively (Figure 1, Figure S27, and Table S19d). Additionally, the sum of each pair (i.e. R' C_{34-35} and C_{36-37}) represent nearly 50% of LCAEs produced (Figure 8f, Figure S29).

3.3.5 The effect of temperature on LCA desaturation

Analysis of the relative abundances of all LCAs and LCEs grouped by their degree of unsaturation (i.e., the combined proportion of di-unsaturated LCAEs relative to total LCAE levels), showed that—as expected—the cellular concentrations of tri- and tetra-unsaturated compounds were higher in both strains when grown at 7°C compared to 20°C (Figure 7a and Table S19). No LCAs nor LCEs with four unsaturations were detected when strains were grown at 20°C. Interestingly, while the relative abundance of tri-unsaturated LCAEs was higher than di-unsaturated LCAEs in strain CCMP1742 grown at 7°C, this was not the case for CCMP2758, where di-unsaturated LCAEs were more abundant than tri-unsaturated ones (Figure 8e).

Evaluation of the degree of unsaturation of the LCAs used in the $U_{37}^{k\prime}$ SST proxy (C_{37} LCAs) revealed significant differences in the $C_{37:3}$ / $C_{37:2}$ and $C_{37:4}$ / $C_{37:3}$ ratios between cultures grown at 7°C and 20°C, consistent across both strains and all sampling points (Figure 7e-f). Overall, $C_{37:2}$, $C_{37:3}$ and $C_{37:4}$ cellular concentrations were lower in strain CCMP2758 than in CCMP1742. When strains were grown at 20°C, concentrations of $C_{37:2}$ per cell increased from t0 to t1, and then decreased. When grown at 7°C, they remained stable or showed a slight decrease across sampling points (Figure S25). When grown at 7°C, both strains produced higher amounts of $C_{37:3}$ (Figure S25), with peak concentrations occurring at t1. In most cases, levels then progressively decreased over time. $C_{37:4}$ was primarily detected in cultures grown at 7°C, where its cellular concentration increased over time, and only trace amounts were detected at 20°C (Figure S25). Both strains showed higher concentrations of $C_{37:3}$ than $C_{37:2}$ at all time points when grown at 7°C (Figure 7c,d and Figure S25). While this is in line with the overall higher relative abundance of tri-unsaturated LCAEs observed in CCMP1742 cultures grown at 7°C it contrasts with the higher relative abundance of di-unsaturated LCAEs observed in CCMP2758 cultures grown at 7°C (Figure 8e). This discrepancy may arise because CCMP2758 synthesizes shorter (C_{35-36}) LCAEs. For instance, the desaturase responsible for the addition of the third unsaturation may have low affinity

for short LCAEs and may act preferentially on longer (C_{37-39}) LCAEs. Thus, such selectivity could explain the higher relative abundance of di-unsaturated LCAEs in CCMP2758.

Thus, to elucidate the desaturases responsible for the formation of $C_{37:3}$ and $C_{37:4}$, we focused on the differences in gene expression between cultures of both strains grown at 20°C and 7°C and harvested at t1, when concentrations of $C_{37:3}$ per cell were the highest (Figure S25).

3.3.6 Differential gene expression across samples

To identify differences in gene expression between the control and cold-adapted cultures we carried out the same approach for strains CCMP1742 and CCMP2758 as for strain CCMP1516, namely we performed principal component analysis (PCA) on variance stabilizing transformed (vst) normalized gene counts and calculated Cook's distance to evaluate the presence of outliers (Figures S31 and S32). One of the CCMP1742 transcriptome samples (62-CCMP1742-20C-t3-R1) showed low sequencing depth—25,931 reads versus an average of 7.17 x 10^7 reads in the other samples (Data S7)—and was excluded from our analysis. Both strains showed a clear separation on the first principal component (PC1, describing 81-86% of the variance) between the control and the cold-adapted cultures (Figure S31). Differential gene expression analysis revealed 684 significantly up-regulated and 1,451 significantly down-regulated genes in CCMP1742, and 1,099 significantly up-regulated and 1,509 significantly down-regulated genes in CCMP2758 as adaptive response to cold (≥1 and ≤-1 LFC, p_{adj}-value <0.01).

3.3.7 Evaluating the expression of FAS and PKSs across time and temperatures

Differential gene expression analysis of FAS- and PKS-related genes between samples grown at different temperatures revealed that 25 genes in CCMP1742 and 43 genes in CCMP2758 were upregulated during t2 when strains were grown at 7°C ($p_{adj} \leq 0.05$, Table S21). During t3 (7°C) and t4 (20°C) 62 genes in CCMP1742 and 68 genes in CCMP2758 were upregulated ($p_{adj} \leq 0.05$, Table S21). In total, 66 FAS- and PKS-related genes in CCMP1742 and 76 in CCMP758 were upregulated during t2 or t3/t4 when strains were grown at 7°C (compared to 20°C). Of these, only 18 genes in CCMP1742 and 35 in CCMP2758 were upregulated at both time points (t2 and t3/t4, $p_{adj} \leq 0.05$, Table S21).

Differential gene expression analysis of FAS- and PKS-related genes across time revealed 35 differentially expressed genes in CCMP1742 and 76 in CCMP2758 ($p_{adj} < 0.05$, Tables S21 and S22) that thus might be involved in LCA formation. All differentially expressed genes were downregulated over time in both strains

Overall, 12 FAS- and PKS-related genes in CCMP1742 and 53 in CCMP2758 were differentially expressed across both time and temperatures, that is: upregulated during t2 or t3/t4 at 7°C, and down-regulated across time (Table S22). Given the observed correlation with total LCAE abundances, the

expression pattern of these genes and their annotated function, these genes constitute a good set of candidates to further study LCA and LCE formation.

Out of the 93 CCMP1742 hits identified as homologs of PKS7, 31 genes were not expressed, and only 2 were differentially expressed across time and temperatures (Table S10). Out of the 95 hits identified as homologs of PKS7 in CCMP2758, 20 were not expressed and 30 were differentially expressed across time and temperatures (Table S10).

To better understand the structure of these PKS7 homologs and assess their module composition and arrangement, we performed sequence homology analyses using PKS7's subunits as query—that is dividing PKS7' long sequence into smaller protein subunits according to their functional annotation as ACP, KS, KR and DH—against the FAS-, PKS- and PKS7-related CCMP1742 and CCMP2758 differentially expressed genes. BGC-CCMP1742_FUN_002252-T1 was the only CCMP1742 protein larger than 2,000 aa that was differentially expressed under our tested conditions and shows some degree of homology to nearly all ACP, KS and DH domains present in PKS7, and only one of its KR domains (Table S10). Due to the repetitive nature of the domains' sequences, it is hard to exactly determine how many subunits are present in CCMP1742_FUN_002252-T1. However, due to its reduced size we estimate that it contains at least one copy of each ACP, KS, KR and DH domains (Table S10). CCMP2758 contains five proteins that are at least half as long as PKS7 (~10,000 aa), namely BGC-CCMP2758_FUN_000054-T1 which was only differentially expressed across temperatures, and BGC-CCMP2758_FUN_000059-T1 to BGC-CCMP2758_FUN_000062-T1, which are two sets of identical proteins that are upregulated at 7°C and downregulated over time at 20°C (Table S22). Our analysis revealed that these proteins show homology to all PKS7's ACP, KS, KR and ER domains, and to 11—out of 12—DH domains (Table S10), suggesting that it might contain more than one copy of each domain.

Additionally, to shed further light on the genes responsible for LCA formation in the reference strain CCMP1516, we also aligned the CCMP1742 and CCMP2758 transcriptomic reads to the CCMP1516 reference genome (Tables S23 and S24). When using CCMP1742 transcriptomic reads against the CCMP1516 reference annotation, we observed that four genes potentially related to LCAE formation were upregulated at 7°C, 12 genes were differentially expressed across time (Table S23), and that two of these genes were differentially expressed in both tested conditions: the gene encoding the polyketide synthases PKS9 and EMIHUDRAFT_364000 (Table S24). When using the transcriptomic reads from CCMP2758 against the CCMP1516 reference annotation, we identified 11 genes that were upregulated at 7°C and 16 genes that were differentially expressed across time (Table S23). Nine of these genes were differentially expressed under both sets of conditions, including the gene EMIHUDRAFT_238529 encoding a fatty acid synthase, and the genes encoding polyketide synthases

EMIHUDRAFT_56457, EMIHUDRAFT_364000, PKS2, PKS6, PKS7, PKS8, PKS9—a large (20,899 aa) PKS closely related to PKS7 based on bi-directional best BLAST hit—and PKS10 (Table S24).

Although the structure of PKS7 suggests that it could synthesize a product of a structure similar to LCAs and LCEs, and the differential gene expression data of CCMP2758 reveals that the expression of PKS7 follows a pattern similar to LCAE abundances—i.e upregulated when strains are grown at 7°C and downregulated over time—PKS7 is not differentially expressed under any of these conditions in strain CCMP1742 based on our transcriptomic read mapping experiments to the CCMP1516 reference annotation (Table S25). Thus, our hypothesis regarding the involvement of PKS7 in LCA biosynthesis cannot be reliable confirmed based only on these data. Nonetheless, we should keep in mind that mapping transcriptomic reads from one strain to the genome of another strain can introduce biases caused by misalignments, read loss and incorrect read mapping, and thus affect differential gene expression analysis due to an increase in the number of false positives and/or false negatives. Therefore, this analysis of transcriptomic read mapping to a different reference genome strain should only be considered as exploratory.

3.3.8 Evaluating the expression of desaturases across temperatures

To identify the genes encoding the proteins responsible for the introduction of the third and fourth double bond of LCAs in *G. huxleyi* CCMP1742 and CCMP2758, we next analyzed a phylogenetic tree containing differentially expressed CCMP1742 and CCMP2758 desaturases, their homologs, EMIHUDRAFT_454486 and its orthologues, AKD1 from *T. lutea*, and *A. thaliana* and *P. tricornutum* annotated desaturases (Figure 9, Figure S33, Table S26 and Data S8). We observed two phylogenetic clusters containing proteins that were closely related to AKD1 and were overexpressed at cold temperatures, and which we call from hereon Gh-AKD1a and Gh-AKD1b (Figure S33 and Table S27).

Gh-AKD1a contains *T. lutea* AKD1 [33], its homologous sequence when annotating the *T. lutea* CCAP 926/14 genome with Funannotate (Tisochrysis_lutea_FUN_014439-T1) [45], one CCMP1742 and two CCMP2758 proteins. Note that the sequences of AKD1 and Tisochrysis_lutea_FUN_014439-T1 are not identical, likely because both sequences originate from different *T. lutea* strains (Figure S33). Tisochrysis_lutea_FUN_014439-T1 originates from of the *T. lutea* CCAP 926/14 genome whereas the AKD1 sequence was obtained from the mRNA sequence of a *T. lutea* strain obtained from the Matsunaga laboratory at the Tokyo University of Agriculture and Technology, and kept and routinely used for alkenone studies at the Shiraiwa laboratory in the University of Tsukuba since the year 2000 [33, 46].

Gh-AKD1b contains EMIHUDRAFT_454486, and its orthologs in CCMP1742 (BGC-CCMP1742_FUN_034149-T1) and CCMP2758 (BGC-CCMP2758_FUN_035665-T1), all of which were

overexpressed at 7°C (Figures S33-S35 and Table S27). EMIHUDRAFT_454486 is not the best hit in terms of bits-score to ADK1 in the CCMP1516 reference annotation (Table S12), but it is the most closely related sequence to ADK1 in our phylogenetic tree of desaturases (Figure S33). Thus, given the phylogenetic positioning of EMIHUDRAFT_454486 (Figure S33), and the fact it was recently confirmed as the desaturase responsible for the addition of the third unsaturation in *G. huxleyi* CCMP1516 by ref [47], it is likely that BGC-CCMP1742_FUN_034149-T1 and BGC-CCMP2758_FUN_035665-T1 are the genes responsible for this reaction in strains CCMP1742 and CCMP2758, respectively.

To identify the desaturase responsible for the fourth unsaturation, we analyzed cold-induced desaturases present in both CCMP1742 and CCMP2758 but either absent or not differentially expressed in CCMP1516, because this strain does not produce tetra-unsaturated LCAs. We identified two clusters that followed these criteria and could contain Gh-AKD2, which we called Gh-AKD2a and Gh-AKD2b (Figure 9 and Figure S33). Although some expression of the CCMP1516 genes encoding the proteins found in clusters Gh-ADK2a (EMIHUDRAFT_217979) and Gh-AKD2b (EMIHUDRAFT_349408 and EMIHUDRAFT_315318) was detected, it was very low in comparison to that of CCMP1742 and CCMP2758, and differences in expression between temperatures were not statistically significant ($p_{adj} > 0.01$, Figures S34, S35, and Table S27).

While several *A. thaliana* and *P. tricornutum* genes encoding methyl-end fatty acid desaturases (Figure 9 and Figure S33) are located close to cluster Gh-AKD2b, support for the placement of their branches is low (bootstrap ≤70 and SH-aLRT ≤85 values), indicating that their close phylogenetic placement to Gh-AKD2b might be an artifact during the construction of the tree and that no definite conclusions regarding the functionality of these CCMP1742 and CCMP2758 desaturases can be drawn from these data alone. Nonetheless, the expression pattern of the proteins found in these two clusters is in agreement with our hypothesis and thus suggests that these genes could be involved in the synthesis of $C_{37:4}$ LCA, making them good candidates for future characterization studies.

Supplementary Materials and Methods

1. Strains and culture conditions

We used three strains of the haptophyte *Gephyrocapsa huxleyi* obtained from the National Center for Marine Algae and Microbiota (NCMA), namely CCMP1516, CCMP1742 and CCMP2758. Strain CCMP1516 was isolated in 1992 from the South Pacific and deposited in the culture collection of the Bigelow National Center for Marine Algae and Microbiota (NCMA), formerly known as Provasoli-Guillard National Center for Culture of Marine Phytoplankton (CCMP). Strain CCMP1742 (also known as NEPCC55a) was originally isolated from the North Pacific and deposited in the CCMP strain collection in 1996. Strain CCMP2758 originated from a laboratory culture of CCMP1742 which started to produce a different LCA profile and was deposited in the NCMA strain collection in 2006 [17].

Strains CCMP1742 and CCMP2758 obtained from NCMA were not axenic, thus at the start of this study these cultures were treated to reduce the bacterial load. First, an actively growing culture was incubated with glucose (1 g L^{-1} final concentration) in the dark to activate bacterial metabolism and increase their susceptibility to antibiotics. Then the cells were collected by centrifugation for 5 minutes at 1500 rpm at room temperature, supernatant was discarded, and cells were resuspended in sterile L1-Si media. The procedure was repeated three more times before the addition of an antibiotic cocktail containing penicillin G (0.8 mg mL^{-1}), streptomycin (0.15 mg mL^{-1}) and gentamycin (0.2 mg mL^{-1}). Once the culture looked green again (approximately after a month), the presence of bacterial contamination was checked by means of DAPI staining (200 µL culture, 1 ml glutaraldehyde 25%, and 50 µL DAPI 100 µg mL^{-1}) and observation under the microscope. Presence of bacteria was observed, and the decontamination procedure was repeated. Cultures were washed by centrifugation four times as described above, then glucose was added and incubated in the dark overnight before the addition of different antibiotic cocktail containing carbenicillin (0.5 mg mL^{-1}), cefotaxime (1 mg mL^{-1}), gentamycin (0.4 mg mL^{-1}), streptomycin (0.3 mg mL^{-1}), penicillin (0.3 mg mL^{-1}), penicillin G (0.36 mg mL^{-1}), chloramphenicol (0.0025 mg mL^{-1}), polymyxin B (15 U mL^{-1}), neomycin (0.003 mg mL^{-1}). Cultures were again checked via DAPI staining and microscopic observation showing that bacteria were still present in the cultures.

Cultures were routinely maintained in 30mL of L1-Si medium [48] in 60 mL borosilicate Erlenmeyer flasks under a 16:8 light:dark regime at approximately 110 µmol photons m^{-2} s^{-1} (measured using a Universal Light Meter-500 (Walz, Germany) equipped with a US-SQS/L sensor) and transferred every week (20°C) or every two weeks (7°C) using 10% of inoculum.

2. Growth experiments

2.1 Cold shock experiments

G. huxleyi strain CCMP1516 cells were initially grown in batch culture in 5 L glass Erlenmeyer flasks at 20°C, under a light:dark cycle of 16:8 h at 100-170 µmol photons $m^{-2} s^{-1}$. Cells were grown up to cell densities of ca. 6.5 x 10^5 cells ml^{-1}, thoroughly mixed, and subsequently transferred into twenty-one 250 ml Erlenmeyer flaks with a wide opening. All flasks were kept at 20°C for 1 day, then nine out of twenty-one Erlenmeyer flasks were randomly selected and immediately transferred to a 10°C room with the same light-dark cycle and level of irradiance (T-0; 2.5 h after the dark cycle finished). The change in *in-situ* growth temperatures of the cultures was monitored using a thermometer inside an additional flask containing *G. huxleyi*. Immediately after transfer to 10°C (T-0), cells of three Erlenmeyer flasks kept at 20°C were filtered over separate pre-combusted glass fiber (GF/F) filters (pore-size of 0.7 µm, 47 mm in diameter, Whatman, Maidstone, UK) using a vacuum pumping system. At later sampling points (T-1, -2, and -3, where the number indicates the hour(s) after T-0) the cells of three flasks, maintained at 20°C and three flasks transferred to 10°C, were collected via filtration. Filters were flash-frozen in liquid nitrogen immediately after filtration and stored at -80°C until further processing.

Another set of cold shock experiments, using *G. huxleyi* strains CCMP1746 and CCMP2758 was also carried out. Eight-liter cultures of each strain were grown in a 10 liter polycarbonate bottle at 15°C under a 16:8 light:dark regime at 100-170 µmol photons $m^{-2} s^{-1}$. Cultures were continuously shaken by means of a magnetic stirrer at 100 rpm. Continuous air bubbling was provided via a glass canula ending at the bottom of the Nalgene bottle and connected to an aquarium air pump through a 0.2 µm pore size filter (Sartorius, Göttingen, Germany). The culture used as inoculum was grown in one-liter L1-Si media in a 2.5 liter Erlenmeyer flask continuously stirred at 100 rpm under the same light and temperature conditions. Upon reaching ca 1.3 x 10^6 cells mL^{-1}, the culture was inoculated into seven liters of L1-Si media resulting in a starting cell density of 1.3-3 x 10^5 cells mL^{-1}. Cell growth was monitored daily and upon reaching 1.7-2.5 x 10^5 cells mL^{-1} cultures were aliquoted in 250 mL Erlenmeyer flasks with a wide opening. Strain CCMP2758 was aliquoted in 33 flasks, each one containing 200 mL, while CCMP1742 was aliquoted in 47 flasks, each one containing 160 mL of culture. All Erlenmeyer flasks were kept at 15°C overnight and three hours after the dark cycle finished, half of the flasks were transferred to the 3°C room with the same light intensity (80-120 µmol photons $m^{-2} s^{-1}$) and light:dark cycle. The temperature change inside the cultures was monitored using a digital thermometer in an additional Erlenmeyer flask filled with media. Immediately after transferring the flasks to 3°C (time zero, t0), the contents of three Erlenmeyer flasks were harvested by filtration using a vacuum pump and two pre-combusted glass fiber (GF/F) filters (47 mm diameter, pore-size 0.7 µm,

Whatman, Maidstone, UK) per flask, each filter containing the biomass present in70-90 mL of culture. Filters were then wrapped in aluminum foil and immediately stored at -80°C. Flasks were harvested in triplicate after 1, 2, 3, 6 and 12 hours. Additionally, for strain CCMP1742, two flasks at each temperature were harvested after 24 and 48h, and three flasks at each temperature after 96h. Cell abundances were monitored during all sampling times using flow cytometry.

2.2 Temperature adaptation experiments

For our second type of experiments, cultures were acclimated from the main stock kept at 15°C to 20°C and 7°C for at least 6 months prior to the experiments. Algal cultures were grown in three-liter batch cultures in 5 liter Nalgene bottles under continuous stirring at 100 rpm using a magnetic stirrer under a 16:8 light:dark regime at 100-170 µmol photons m^{-2} s^{-1} . Cultures were inoculated at a starting cell density of 1 x 10^5 cells mL^{-1} using a 300 mL starter culture grown up to a cell density of 1.85-2.52 x 10^6 cells mL^{-1} in a 500mL Erlenmeyer flask grown under the same conditions as the three-liter culture. Culture growth was monitored daily using flow cytometry. Upon reaching a cell density of 1.8-2.8 x 10^5 cells mL^{-1}, the three-liter culture was distributed in 160 mL aliquots amongst fifteen 250 mL Erlenmeyer flasks with a wide opening. Light intensity across different positions in the incubation chamber was measured and flasks were distributed so that cultures harvested at equivalent time points were exposed to comparable light intensities. Cells were harvested at six different sampling (T) points based on cell densities rather than time, in an attempt to capture the different metabolic state of the cells at different growth stages; T0 (1.8-2.8 x 10^5 cells mL^{-1}), T1 (4.7-5.6 x 10^5 cells mL^{-1}), T2 (1.5-1.9 x 10^6 cells mL^{-1}), T3 (1.8-2.9 x 10^6 cells mL^{-1}), T4 (2-3.7 x 10^6 cells mL^{-1}), T5 (2.3-3.4 x 10^6 cells mL^{-1}). Cell growth was monitored daily using at least six flasks; the same three flasks were measured daily throughout the experiment and were harvested at the end of the experiment (T5), whilst the other three measured flasks corresponded to the next sampling point and thus varied throughout the experiment. Cell abundances of each sampled flask were usually measured only once using flow cytometry. However, after noticing inconsistent fluctuations in cell concentrations within the same measured flask in cultures grown at 7°C at around day 17 (approximately from T2 onwards), we introduced dilution replicates, where each sample was diluted three times for flow cytometry measurements. Cells were harvested by filtering 50 mL of culture over pre-combusted glass fiber (GF/F) filters (47mm diameter, pore-size 0.7µm) using a vacuum pump. Upon collection, filters were immediately stored at -80°C until further processing.

2.3 Algal biomass for DNA extraction for Nanopore and Illumina sequencing

Algal biomass used for extraction of DNA for long and short read sequencing was grown in eight-liter batches using the same cultivation set up as described for the cold shock experiments. Cultures were inoculated at a starting cell density of approximately 8 x 10^5 cells mL^{-1} using the appropriate volume of

a one-liter culture grown up to a cell concentration of 1-2 x 10^5 cells mL^{-1} in a 2.5-liter Erlenmeyer flask under constant stirring (100 rpm). Biomass from the culture flask was harvested by pumping the culture into a continuous centrifuge (Heraeus Contrifuge Stratos, Thermo Electron LED, Osterode, Germany) set to 5000 using a peristaltic pump (Masterflex L/S easy load II, Masterflex, Vernon Hills, IL, USA) at a flow rate of 200 mL min^{-1}. The rotor was cleaned using one liter of ultrapure water, 100 mL hypochlorite 1% solution followed by one liter of ultrapure water and one liter of autoclaved seawater prior to culture centrifugation. After c.a. 6 liters of CCMP1742 and 5 liters of CCMP2758 had been centrifuged the sedimented cultures were washed by pumping an additional one liter of sterilized filtered seawater in the continuous centrifuge. Upon disassembling the rotor, the remaining supernatant was removed, and the pellet was collected using a pipette with a cut-off tip. Cells were further concentrated at room temperature on a bench top centrifuge (Eppendorf Centrifuge 5810R) set at 4000 rpm for 20 minutes. Pellets were then immediately frozen using liquid nitrogen and stored at -80°C until further processing.

3.Lipid extraction and analysis

LCAs and LCEs were extracted from the biomass collected on GF/F filters, using ½ of each GF/F filter for extraction. Prior to extraction, a known amount of nonadecan-10-one was added as internal standard to the freeze-dried filters containing *G. huxleyi* CCMP1516 biomass. Freeze-dried filters were cut in small pieces and extracted four to six times using 2:1 (v/v) dichloromethane:methanol (DCM:MeOH) in a sonication bath for 5 to 10 min. The extracts obtained after each sonication step were pooled and dried under a nitrogen stream. The total lipid extract (TLE) was then resuspended in 1:1 (v/v) DCM:MeOH and a known amount of nonadecan-10-one was added to the TLEs of *G. huxleyi* strains CCMP1742 and CCMP2758 as internal standard. Subsequent sample processing differed by experiment. For samples from the cold-shock experiment using strain CCMP1516 and from the cold adaptation experiments of strains CCMP1742 and CCMP2758, the TLE's were separated into three fractions using an aluminum oxide column: an apolar fraction (eluted with four column volumes of 9:1 (v/v) hexane:DCM), a ketone fraction (eluted with three column volumes of 1:1 (v/v) hexane : DCM) and a polar fraction eluted with (1:1 (v/v) DCM:MeOH). Following separation, ketone fractions were dried under a nitrogen stream, dissolved in ethyl acetate (EtAc), and analyzed using gas chromatography (GC). In contrast, TLE's obtained from the cold-shock experiments using strains CCMP1742 and CCMP2758 were passed through an aluminum oxide column and eluted with only 1:1 (v/v) DCM:MeOH, yielding a single polar fraction that contained all ketones, polar and apolar compounds. Polar fractions were then placed in a vial with an insert, dried and silylated using 10µL pyridine and 10µL N,O-Bis(trimethylsilyl)trifluoroacetamide (BSTFA, Regis Technologies Inc., IL, USA), and allowed to react at 60°C for 30 min before being dissolved in EtAc for GC-MS analysis. Ketone

fractions from strain CCMP1516 were analyzed on an Agilent 6890 instrument equipped with an Agilent CP-Sil 5 CB column (50 m x 0.32 mm i.d.; 0.12 µm film thickness). The temperature program was: 70–200°C at 20°C min^{-1}, then at 3°C min^{-1} to 320°C (held for 25 min). Ketone and silylated polar fractions of strain CCMP1742 and CCMP2758 were analyzed using an Agilent 7890B gas chromatograph with a flame ionization detector equipped with an RTX-200 column (Restek, 60 m x 0.32 mm x 0.5 µm). Concentrations of LCAs and LCEs present in the analyzed fractions were calculated in relation to the amount of nonadecane-10-one standard added. Compounds present in the ketone fraction were identified using a gas chromatograph (Agilent Technologies 7890B GC system) also equipped with a 60 m RTX-200 column and coupled to a mass spectrometer (Agilent Technologies 7000C GC/MS Triple Quad). The temperature program was: 70°C to 250°C at 15°C min^{-1}, 250-320°C at 1.5°C min^{-1}, then kept at 320°C for 25min at a flow rate of 1.5 mL min^{-1}.

The concentration of each compound was calculated in relation to the amount of internal standard added to the ketone fraction and then normalized to the number of cells used for lipid extraction. The total concentration of compounds in the ketone fraction was calculated by addition of the concentration of each individual compound after cell normalization. To analyze the relative abundances of the different compound characteristics (Table S18) the concentrations of each compound containing the factor to be analyzed were added and then divided by the total concentration of compounds in the ketone fraction.

To ensure proper identification of peaks corresponding to the fatty acid esters and to distinguish them from peaks corresponding to alkenones, we selected a subset of ketone fractions to be further processed using base hydrolysis. Half of the ketone fraction was saponified using 2 mL 1N KOH / MeOH and refluxed for 1h at 145°C under constant stirring. After cooling, 2 mL of bi-distilled water was added, and the pH of the sample was adjusted to pH = 4 using 2N HCl / MeOH. Lipids were extracted from the solution by addition of 2 mL DCM, vortexing and recovery of the bottom phase. This was repeated two additional times using 1 mL of DCM. DCM extracts were combined, dried under a nitrogen stream and dissolved in 1:1 (v/v) hexane:DCM prior to purification over an aluminum oxide column from which two fractions were obtained: a ketone fraction (eluted with 3 column volumes of 1:1 (v/v) hexane:DCM) and a polar fraction eluted with (1:1 (v/v) DCM:MeOH).

4. Nucleic acids extraction and sequencing

RNA was isolated from *G. huxleyi* CCMP1516 samples collected during the second 3 h cold shock experiment using two quarters of each glass fibre filter obtained at T-0 until T-3 of the experiment (with the number indicating the hour(s) after the start of the experiment, containing triplicates per

time point per condition). RNA was isolated for each quarter of the filter as described below, and the end solutions were pooled.

RNA was also extracted from samples of *G. huxleyi* strains CCMP1742 and CCMP2758 collected during the temperature adaptation experiment. A subset of cultures harvested at comparable growth stages across strains and temperatures was selected, namely: t0, t1 and t2 for both strains grown at 20°C and 7°C, t3 for both strains grown at 7°C and t4 for both strains grown at 20°C (see Figure S16 for details). Each sampling point contained three biological replicates per strain and condition, except for t0 which was harvested from the main three-liter culture before aliquoting and only contained one replicate. RNA was extracted from one quarter of each of glass fiber GF/F filter using the Qiagen RNeasy mini kit (Qiagen, Valencia, CA, USA) according to the manufacturer's instructions with the following modifications: after addition of 1 mL lysis buffer supplemented with 10 µl β-mercaptoethanol to the cut filters, 50µl of Ambion™ Plant RNA isolation Aid (Ambion™, Lithuania) were added to optimize RNA extraction. Filters were bead beated using a mixture of 0.2 mL 0.5 mm diameter and 0.2 mL 0.1 mm diameter glass beads (BioSpec Products, Bartlesville, OK, USA) twice for 30 seconds at a speed of 4 m s^{-1} with a 30 second dwell using a bead mill homogenizer (Bead Ruptor Elite, Omni International, Kennesaw, GA, USA). After centrifugation of the extracts, 2 µl of SUPERase In RNase Inhibitor 20 U μl^{-1} (Invitrogen, Carlsbad, CA, USA) was added to the cleared lysate. On-column DNA digestion during RNA extraction was performed using On-Column DNAse I Digestion Set (Sigma-Aldrich, St Louis, MO, USA) according to the manufacturer's instructions. Additionally, RNA eluates were treated twice with TURBO DNA-free kit to remove possible DNA contamination. PCR was performed on RNA extracts to confirm the absence of DNA.

RNA concentrations were measured using Nanodrop ND-1000 Spectrophotometer (NanoDrop Technologies Inc. Wilmington, DE, USA) or Qubit RNA HS Assay Kit on a Qubit 3.0 Fluorometer (Qubit, Thermo Fischer Scientific, Waltham, MA, USA). The quality and integrity of the RNA extracts was assessed using an Agilent RNA 6000 Nano Kit on a Bioanalyzer 2100 (Agilent Technologies, Santa Clara, CA, USA).

TruSeq Stranded mRNA polyA library preparations were carried out by Utrecht Sequencing Facility (USEQ, Utrecht, The Netherlands). RNA fragments of CCMP1742 and CCMP2758 were barcoded using Unique Molecular Identifiers (UMIs) (9 bp length) during library preparation. Samples were sequenced using either two runs of 1x 75bp on an Illumina Nextseq 500 platform (CCMP1516) or a S2, 2 x150 bp run on an Illumina NovaSeq 6000 platform (CCMP1742 and CCMP2758).

For extraction of unsheared and high molecular weight DNA to carry out Nanopore sequencing from strains CCMP1742 and CCMP2758, an amount of concentrated pellet equivalent to approximately 500 mL of culture (as previously described in the "2.3 Algal biomass for DNA extraction for Nanopore and

Illumina sequencing" section) was used. Each pellet was resuspended in a solution containing 800 µl of CTAB – Lysis buffer *Biochemica* (PanReac AppliChem, Darmstadt, Germany), 0.8 mg mL^{-1} lysozyme (Thermo Scientific™, Waltham, MA), 5 µg mL^{-1} RNAse A (Qiagen, Mat. No. 1007885) and incubated at 37°C for 60 min, whilst gently shaking the tube every 10 min. To inactivate potential DNAses present in the reagent, RNase A solution (Qiagen, Valencia, CA, USA) was incubated at 50°C for 10 min and then added to the lysate-CTAB mix. Afterwards a solution containing RNA grade-Proteinase K (Invitrogen, Carlsbad, CA, USA) and SDS (Ambion, Invitrogen, Carlsbad, CA, USA) were added to the mixture at a final concentration of 0.2 mg mL^{-1} and 0.5% respectively, and incubated at 60°C for 60 min, whilst gently shaking the tube every 10 min. After incubation, one volume of Phenol:Chloroform:Isoamyl alcohol (25:24:1) was added and mixed by gently inverting the tube. Samples were then centrifuged at room temperature for 15 min at 1500 g, the top aqueous phase was recovered in a new Eppendorf tube and mixed with one volume of Chloroform:Isoamyl alcohol (24:1) by gently inverting the tube. After centrifugation at 1500 g for 5 min, the aqueous phase was recovered, mixed with 1 volume of Chloroform and centrifuged. The DNA present in the recovered aqueous phase was precipitated via overnight incubation at -20°C in a solution containing: 1:100 vol of Glycogen solution (5 mg mL^{-1}) (Invitrogen, Vilnius, Lithuania), 1:10 (v/v) 3 M Sodium acetate pH 5.5 and 2.5x (v/v) cold ethanol 100%. After overnight precipitation DNA was pelleted by centrifugation for 20 min at 13 000 rpm and 4°C. Supernatant was discarded and the pellet was washed five times as follows: pellet was resuspended with cold ethanol 70%, centrifuged for 5 min at 13 000 rpm (4°C) and incubated at 37°C until dry. DNA was eluted using 50 µl Tris-EDTA (10mM tris, 1 mM EDTA at pH 8) (Invitrogen, Vilnius, Lithuania) and incubated at 37°C for 30 min. Quality of the high molecular weight DNA extracts was assessed by running a dilution of the extracts on a 1 % agarose gel.

DNA library preparations and sequencing of the genomic extracts were carried out by USEQ, each strain was sequenced using individual Nanopore MinION runs (Oxford Nanopore Technologies, Oxford, UK) and one NextSeq500 Mid output flow cell, 2 x 150 bp. The MinION sequencing run was conducted for 72 hours using a FLO-MIN106 flow cell and the SQK-LSK109 kit. The run was managed with MiniKNOW core v3.6.5 and Bream v4.3.16, and high-accuracy base calling was performed using Guppy 3.2.10

5. Functional annotation of genomes using Funannotate

Genome assemblies were annotated using Funannotate. The sort, mask, train, predict and update utilities were run using Funannotate v 1.8.7, whilst the annotate and compare commands were run using Funannotate v 1.8.9. Funannotate sort and mask were run using default parameters. Funannotate train was run supplying forward and reverse RNA Illumina reads, and a transcriptome assembly generated using Trinity (v 2.11.0) [49]. Additional selected options were: --stranded RF, --

max_intronlen 500 , --species "Emiliania huxleyi". After a first attempt at running the train command, contigs not containing all 4 letters (A, T, G and C) were flagged by Funannotate train; these contigs were removed using a custom script before proceeding again with Funannotate train. GeneMark-ES (v 4.68) [50] and tRNAscan-SE (v 2.0.7) [51] were run independently and the resulting files were supplied to Funannotate predict which was run using the following parameters: --species "Emiliania huxleyi", --organism other, --ploidy 2, --repeats2evm, --busco_db protists, --max_intronlen 500, --optimize_augustus. Funannotate update was run on the output of Funannotate predict using default parameters. Funannotate iprscan was run locally with default parameters. Funannotate remote was used to run antiSMASH (-a plants, v 1.0) [52] and Phobius (-short, v 1.01)[53], however antiSMASH did not return any results. EggNOG-mapper (v 2.1.6, database v 5.0.2) [54, 55] and SignalP (v 5.0) [56] were run independently and their results, together with the output obtained from InterProScan (v 5.52-86.0) [57] and Phobius were supplied to Funannotate annotate after selecting the protists BUSCO database (--busco_db).

6. Genome comparison

Analysis of orthologous gene clusters amongst selected haptophytes was carried out using the OrthoFinder [36] algorithm within OrthoVenn3 [37] with default parameters (E-value: 1e-2, Inflation value: 1.5). The genomes compared included the genomes *G. huxleyi* strains CCMP1742 and CCMP2758 (sequenced in this study), *G. huxleyi* CCMP1516 [2] and *T. lutea* [45] annotated with Funannotate and the Reference genome annotation of CCMP1516 (GCF_000372725.1).

Comparison of the genome annotations was also carried out using Funannotate compare. To include the *G. huxleyi* CCMP1516 reference genome [2] in the comparison the original file was converted to gbk format using Funannotate annotate, and clusters of orthologous groups (COGs) and protein functions were predicted separately using EggNOG-mapper and InterProScan.

Genome similarity visualizations (Figure S6) were generated using Python 3 with the following libraries: Pandas (v0.25.0) [58], Seaborn (v0.9.0) [59], NumPy (v1.16.6) [60], and Matplotlib (v3.1.1) [61]

7. Identification of candidate genes in CCMP1742 and CCMP2758 involved in LCA biosynthesis

To identify genes potentially involved in LCA desaturation we searched genome annotations to include genes described using the following keywords (Table S28): “desaturase”, “DES”, “IPR015876”, “GO:0016717”, “EC_number=1.14.19”, “GO:0016491”, and excluding genes containing the words “cytochrome” and “carotenoid” in their functional annotation. In addition, to identify genes potentially involved in LCA biosynthesis, we searched genome annotations using specific terms related to fatty acid and polyketide biosynthetic pathways, including: "acyl carrier protein", "acyl-carrier-protein",

"ACP", "fatty acid", "fatty acid synthase", "FAS", "polyketide", "polyketide synthase" and "PKS". At the same time, to narrow down the results and ensure we were targeting only the most relevant gene categories for our study we excluded certain terms from our search, such as "desaturase", "fasciclin", “YacP”, “pacifastin”, “ACPT” and "cyclase".

8. Building the desaturases phylogenetic tree

To generate the phylogenetic tree of desaturases in Figure 9 and Figure S33 we used the protein sequences of i) upregulated CCMP1742 and CCMP2758 desaturase genes (log fold change estimate ≥ 1 and a false discovery rate-adjusted p-value ≤0.01), ii) their orthologs in CCMP1516, CMP 1742 and CCMP2758 to include proteins that might have not been annotated as desaturases in all three genomes (Table 2), iii) the sequence of AKD1, and of iv) proteins found in clusters related to EMIHUDRAFT_454486 as identified by OrthoVenn but not included in i) or ii), namely BGC-CCMP1742_FUN_014439-T1, BGC-CCMP2758_FUN_015459-T1 and Tisochrysis-lutea_FUN_014022-T1 (Table S13), and v) the sequences of already annotated or characterized desaturases from the pennate diatom *Phaeodactylum tricornutum* and higher plant *Arabidopsis thaliana* (dataset curated and previously used in ref. [33]) with the following sequence IDs: *A thaliana*; NP_194433, NP_172098, NP_565721, NP_188208, NP_172124, NP_566529, NP_187819, NP_180559, NP_194824, NP_187727, NP_196177, NP_181899; *P. tricornutum*: XP_002181794.1, XP_002185732.1, XP_002182858.1, XP_002182901.1, XP_002185374.1, XP_002183026.1, XP_002186139.1, XP_002182832.1, XP_002185498.1. Note that this list does not include all sequences from the original dataset, as some are no longer present in the queried databases. To generate the phylogenetic tree, protein sequences were aligned using MAFFT (v7.407) with the L-INS-i iterative refinement method [62] and poorly aligned regions were removed using trimAl [63]. The phylogenetic tree was built using IQ-tree (v1.6.7) and its in-built nucleotide substitution Model Finder [64], using 1,000 replicates to perform SH-like approximate likelihood ratio test (SH-aLRT) [65] and 1,000 bootstrap replicates, and visualized using iTOL (v6.7.4) [66].

Supplementary Figures

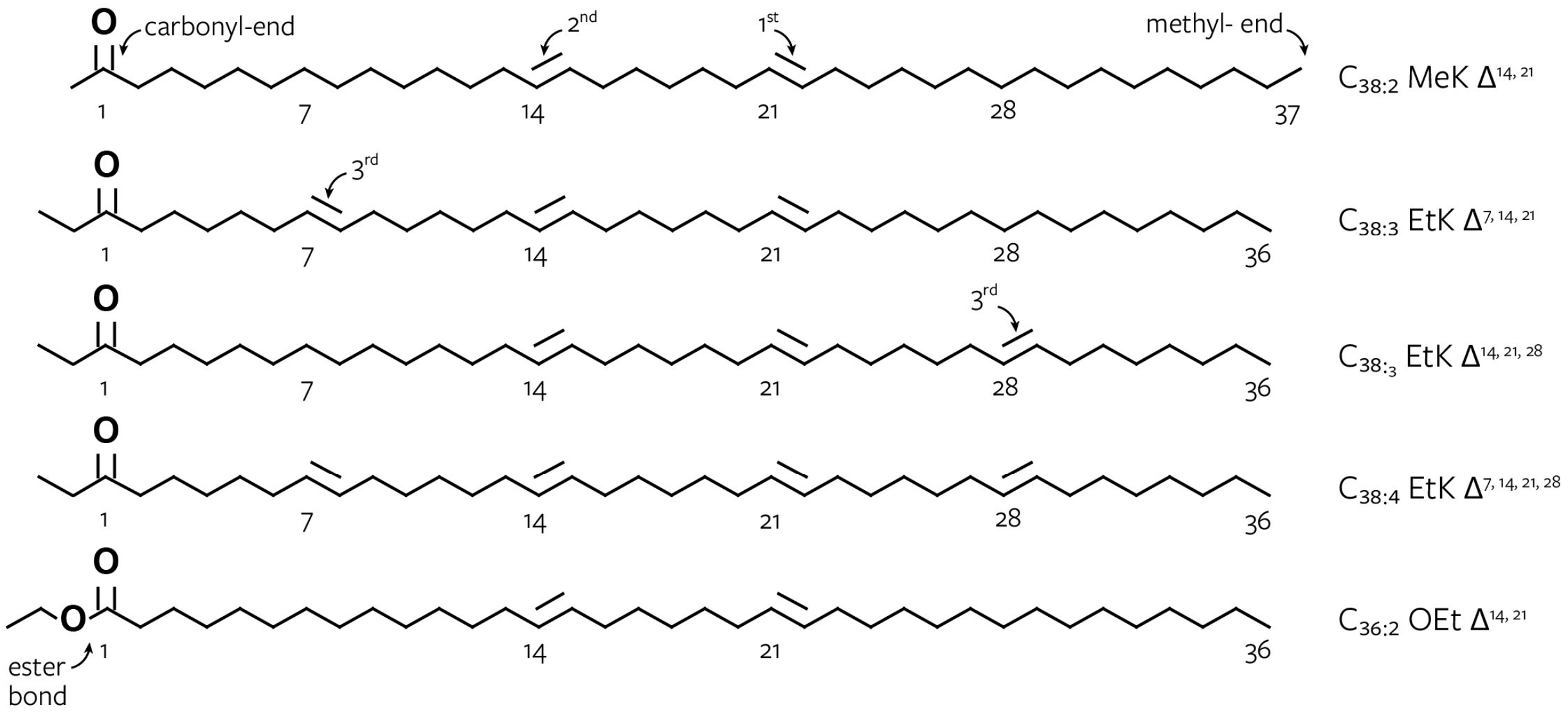

Figure S1. Schematic molecular representation analogous to Figure 1 showing the molecular structures of LCAs and LCEs containing a total of 38 carbons. From top to bottom: methyl $C_{38:2}$ LCA ($C_{38:2}$ MeK) [19], ethyl $C_{38:3}$ LCAs ($C_{38:3}$ EtK) containing the third unsaturation at positions Δ^7 [67] or Δ^{28} [31], ethyl LCA $C_{38:4}$ ($C_{38:4}$ EtK) [68]and the long chain ester (LCEs) or Fatty Acid Ethyl Ester (FAEE) $C_{36:3}$ OEt. Double bond positions in $C_{36:3}$ OEt have not been confirmed and are inferred based on the unsaturation positions of LCAs.

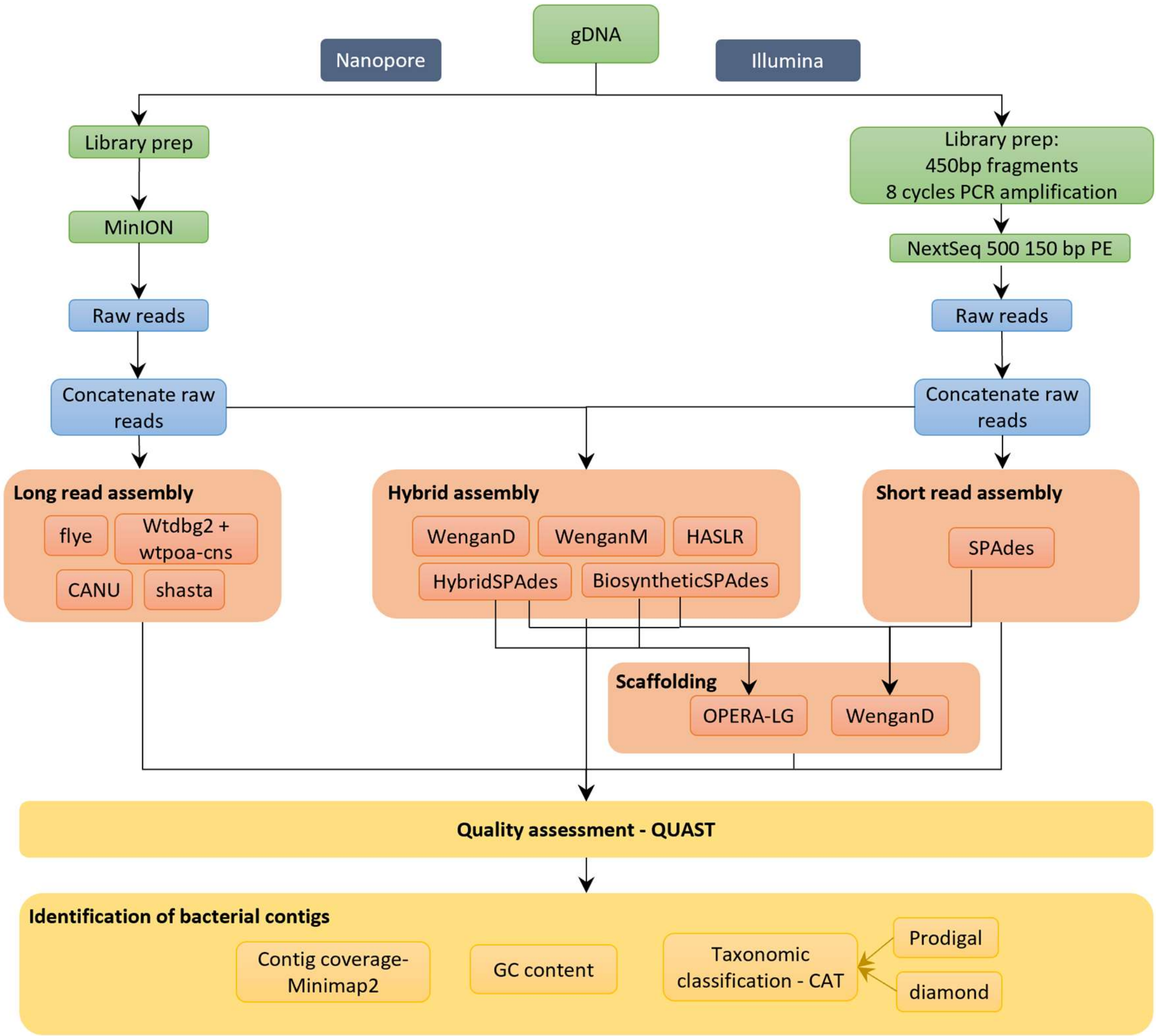

Figure S2. Workflow outlining the process used to evaluate and compare various long-read, short-read, and hybrid genome assemblers.

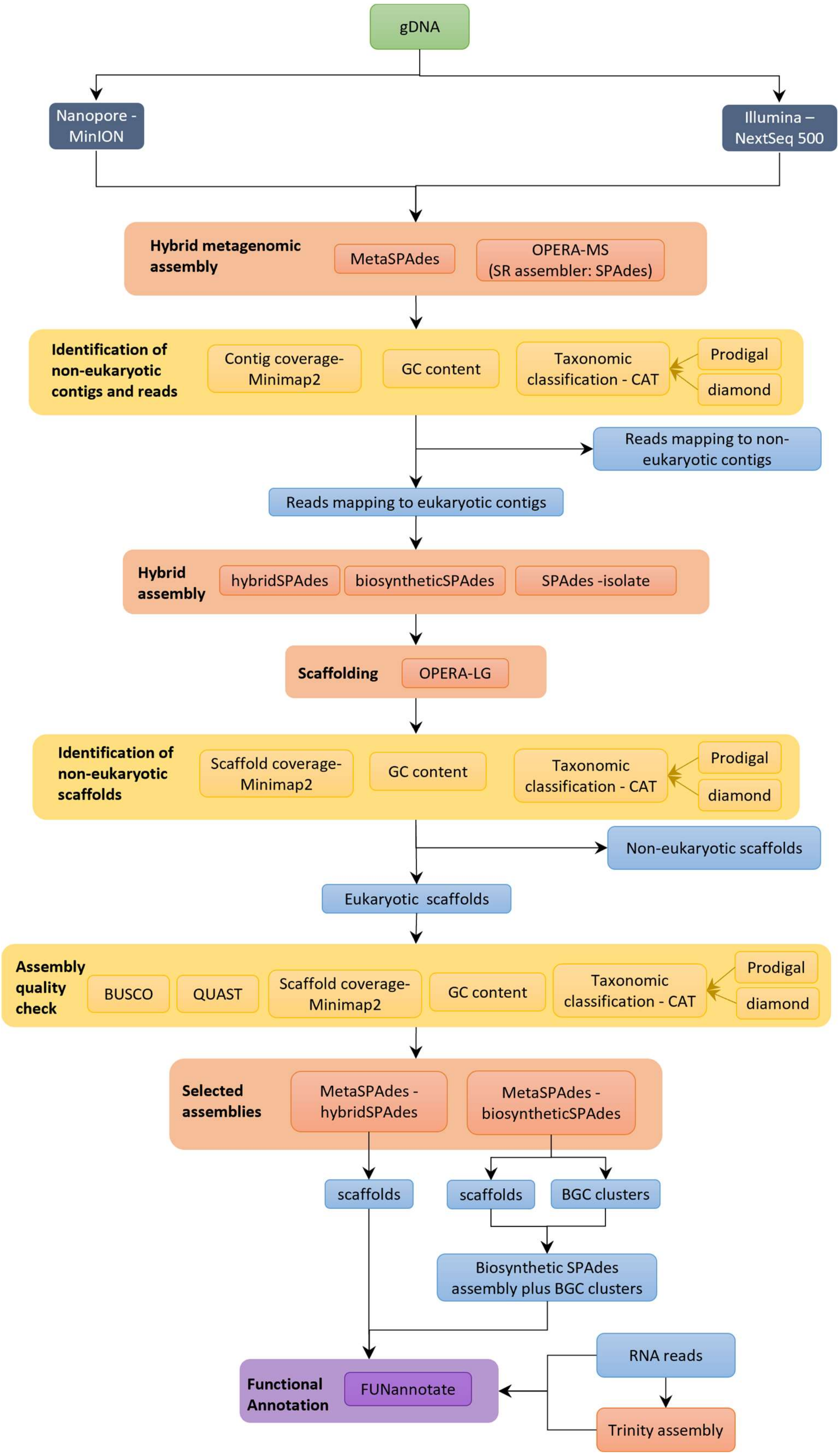

Figure S3. Detailed diagram illustrating the workflow for assembling the CCMP1742 and CCMP2758 draft genomes, including the steps for identifying and removing contaminant DNA. This figure provides a more comprehensive version of Figure 3.

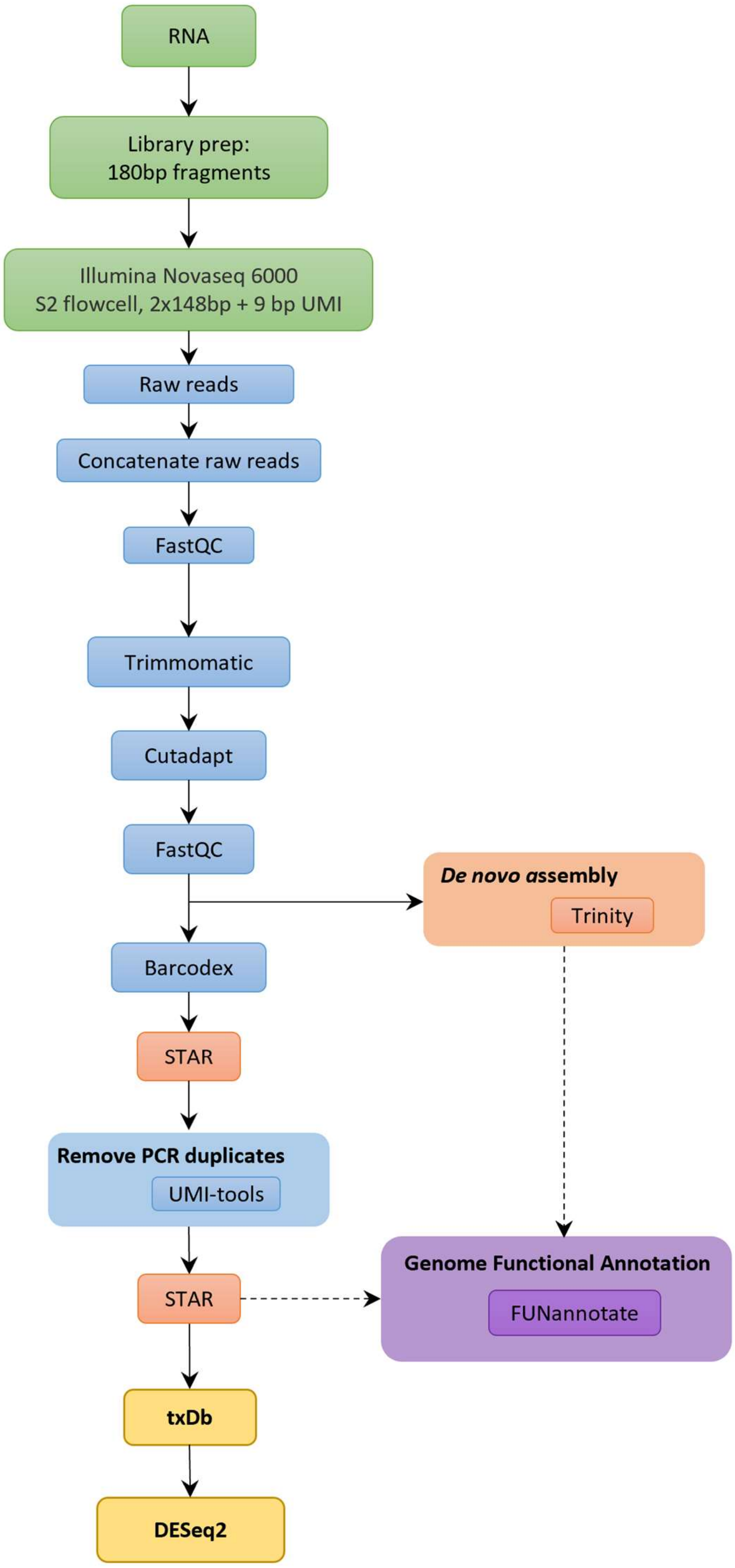

Figure S4. Workflow outlining the process used to generate the transcriptome assembly and perform RNA-seq differential expression analysis.

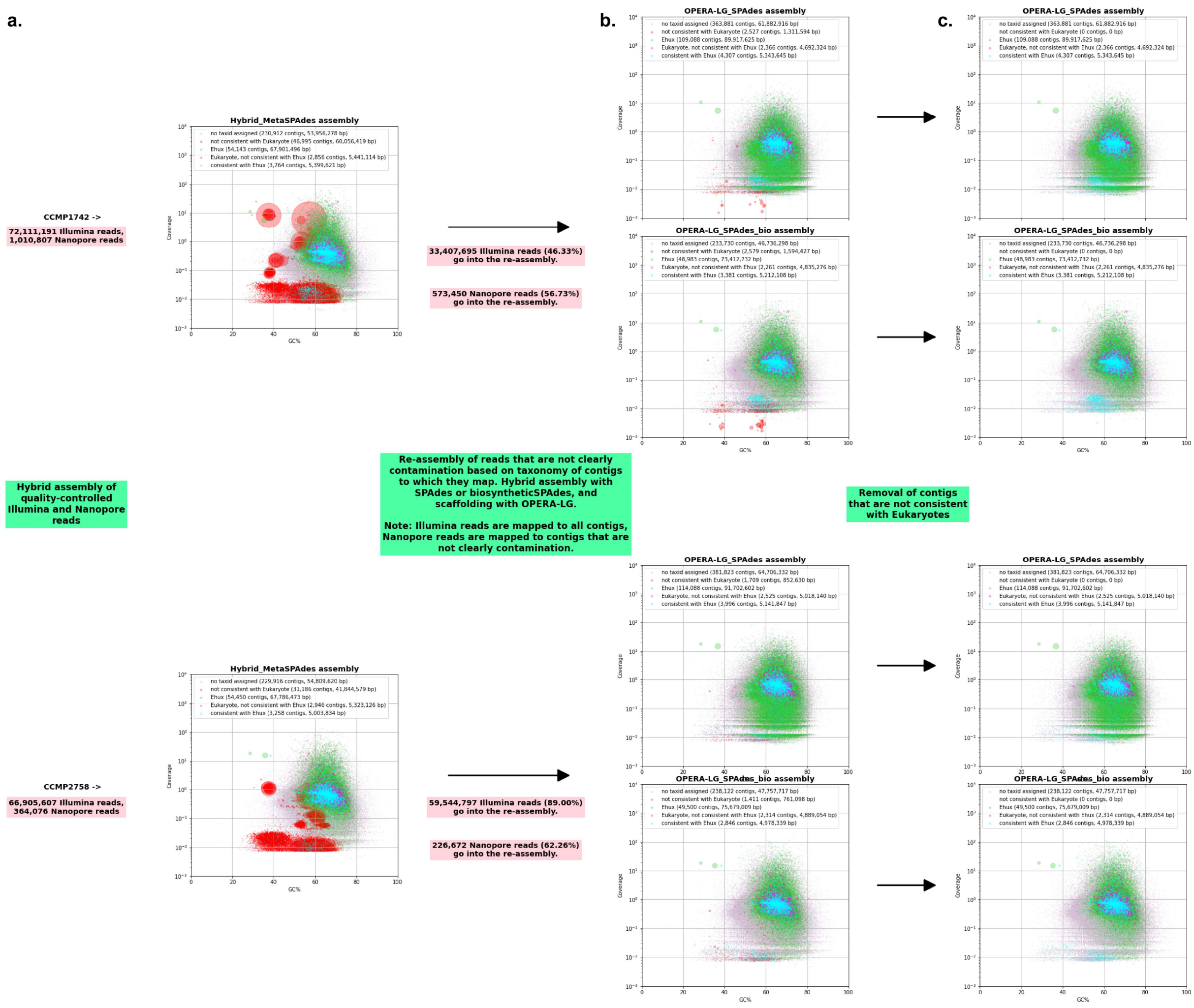

Figure S5. Read coverage versus GC% of contigs and scaffolds and their taxonomic annotation using CAT, obtained during different steps of the assembly and decontamination workflow. **(a)** Results obtained for metagenomic hybrid assemblies (step 2 in Figure 3), **(b)** scaffolds obtained after re-assembly of reads that were not clearly contamination (step 5 in Figure 3), and **(c)** results obtained after removal of scaffolds that were not consistent with eukaryotes (step 6 in Figure 3). Colours indicate different CAT taxonomic annotation; no taxonomy assigned (grey), annotation not consistent with eukaryotes (red), *G. huxleyi* (green), annotation consistent with eukaryotes but not with *G. huxleyi* (pink), and annotation at a higher rank than species but consistent with *G. huxleyi* (blue).

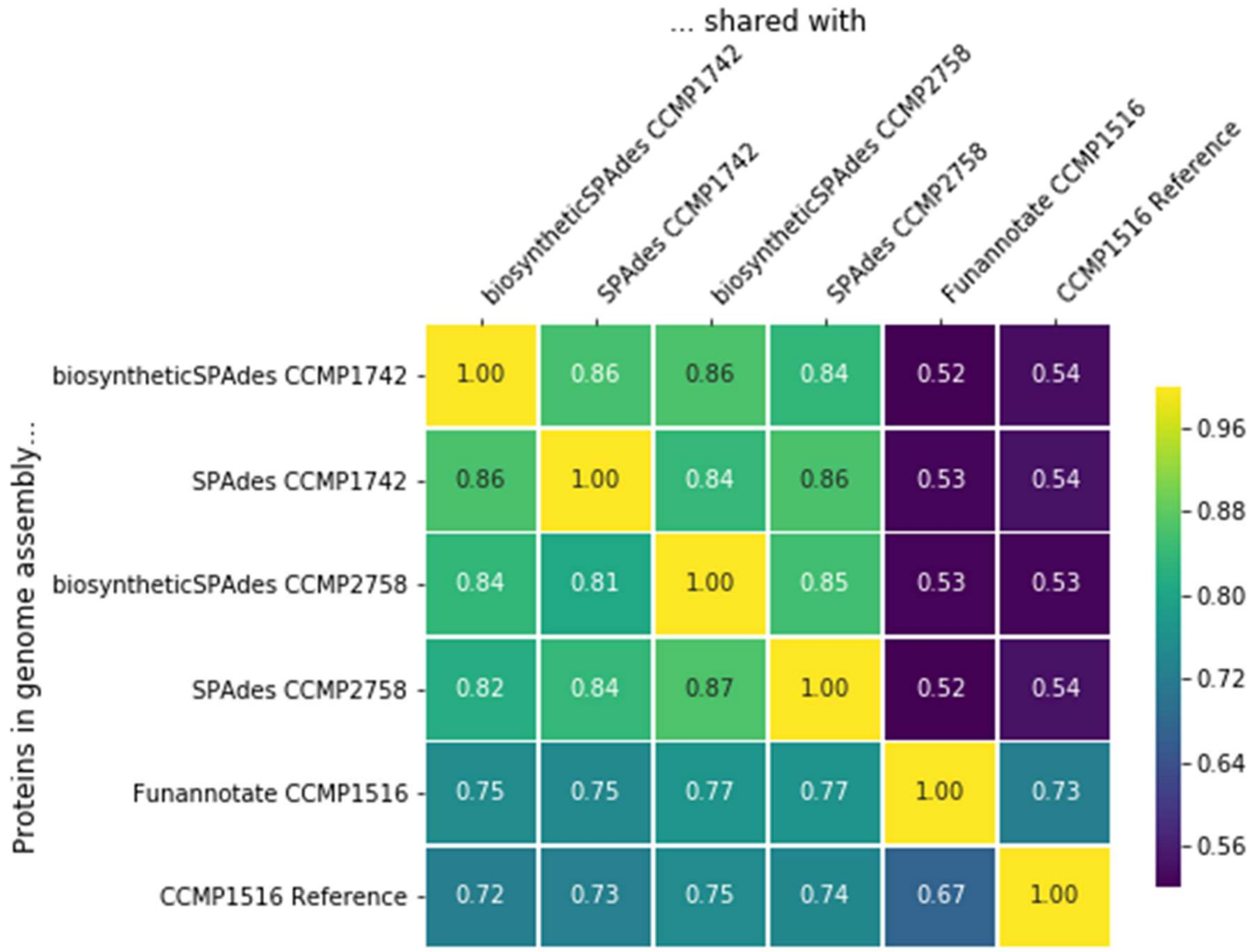

Figure S6. Heatmap showing the fraction of proteins shared between different genome assemblies and annotations, based on OrthoFinder All evaluated assemblies were annotated using Funannotate in this study, except for the CCMP1516 reference annotation, which was annotated with the JGI pipeline in ref [2].The assemblies on the y axes were used as reference to calculate the fraction of shared proteins

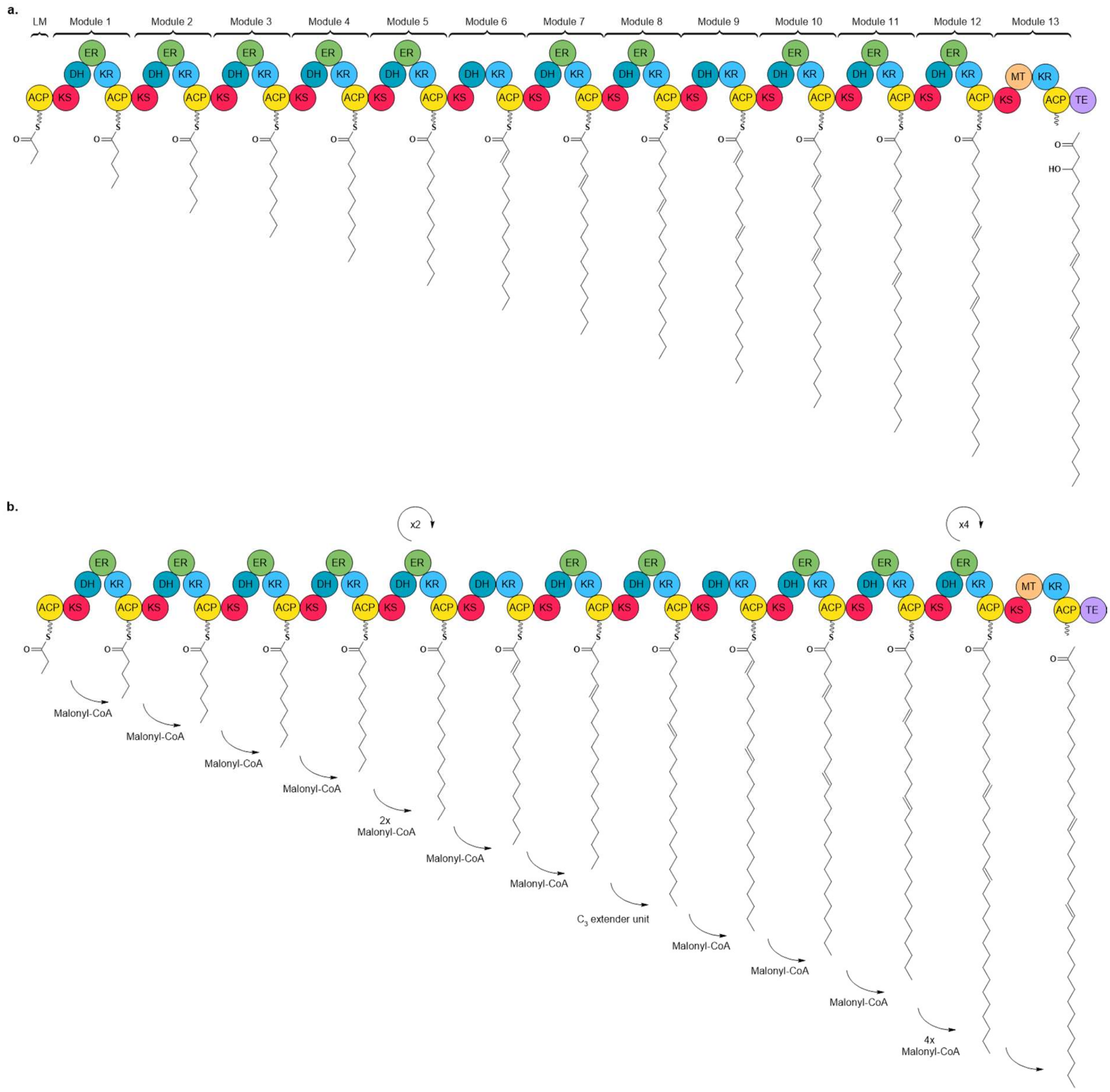

Figure S7. Schematic representation of PKS7. **(a)** Hypothetical products derived from Propionyl-CoA as the starter unit, with each module catalyzing a single elongation step using Malonyl-CoA. **(b)** Tentative pathway for the synthesis of $C_{37:2}$ LCA, using Propionyl-CoA as the starter unit, and Malonyl-CoA and a C_3 molecule as extender units. This pathway accommodates modules capable of catalyzing multiple elongation rounds. Circular arrows denote such modules, with 2x and 4x indicating two and four elongation rounds, respectively. We note that this representation is tentative; for instance, instead of module 5 carrying out an elongation round twice, it could be any of modules 1–5. The assignment of multiple rounds to module 12 is also speculative; it could be carried out by any of modules 10–12. Alternatively, the total number of elongation rounds could be distributed across several distinct modules (for instance, modules 10–12 could each mediate two rounds instead of a single module mediating four).LM, loading module; AT, acyl transferase; ACP, acyl carrier protein; KS, β-ketoacyl synthase; DH, dehydratase; ER, enoyl reductase; KR, ketoreductase; MT, methyltransferase; TE, Thioesterase.

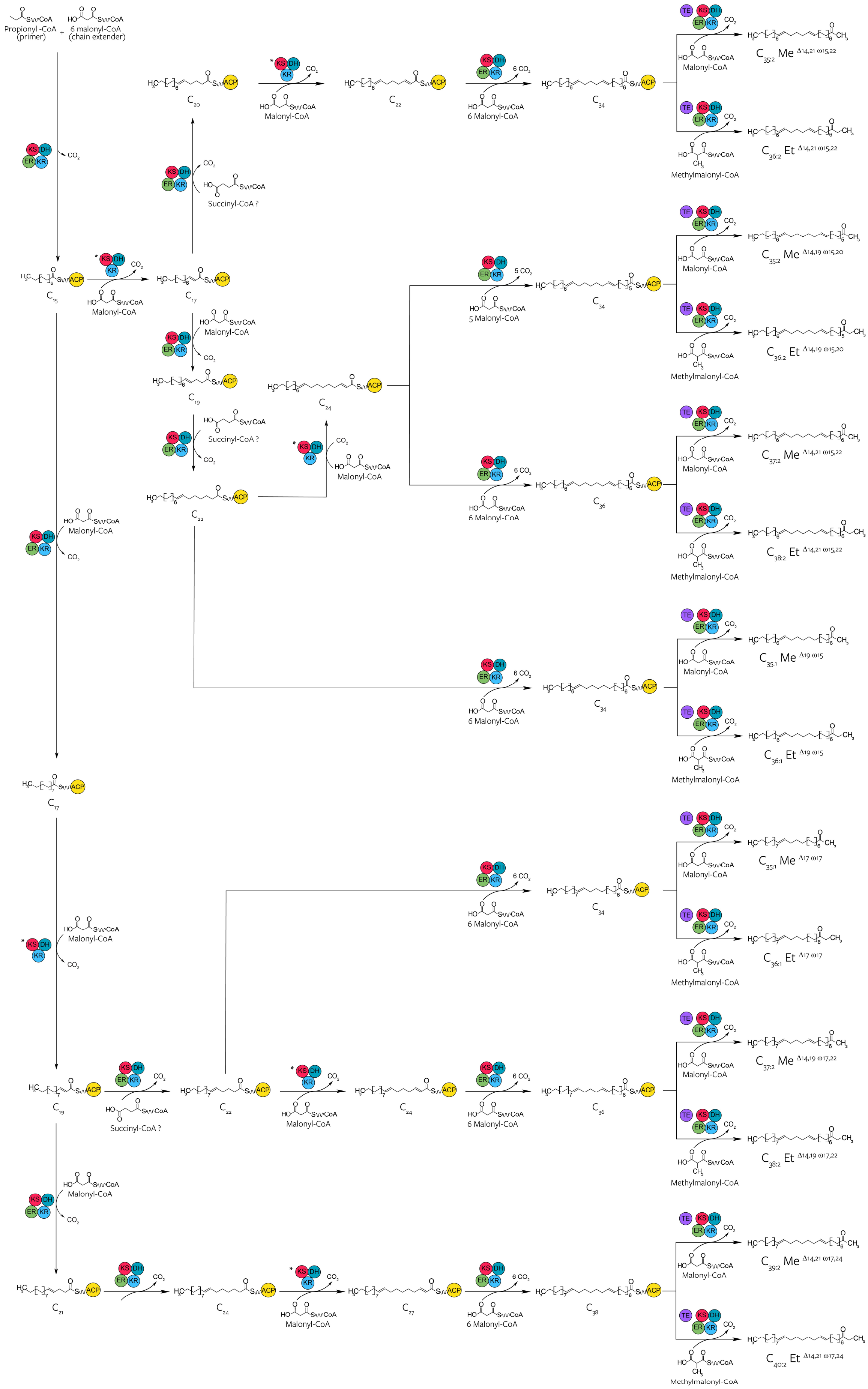
Propionyl -CoA (primer)
6 malonyl-CoA (chain extender)
Malonyl-CoA
6 Malonyl-CoA
5 Malonyl-CoA
Succinyl-CoA ?
Methylmalonyl-CoA
TE
KS
DH
ER
KR
ACP

Figure S8. Proposed pathway for the synthesis of $C_{35:2}$ MeK $\Delta^{14,21}$, $C_{36:2}$ EtK $\Delta^{14,21}$, $C_{35:2}$ MeK $\Delta^{14,19}$, $C_{36:2}$ MeK $\Delta^{14,19}$, $C_{37:2}$ MeK $\Delta^{14,21}$, $C_{38:2}$ EtK $\Delta^{14,21}$, $C_{35:1}$ MeK Δ^{19} , $C_{36:1}$ MeK Δ^{19}, $C_{35:1}$ MeK Δ^{17} , $C_{36:1}$ MeK Δ^{17}, $C_{37:2}$ MeK $\Delta^{14,19}$, $C_{38:2}$ EtK $\Delta^{14,19}$, $C_{39:2}$ MeK $\Delta^{14,21}$ and $C_{40:2}$ Etk $\Delta^{14,21}$ LCAs using Propionyl-CoA as starter unit. Modules lacking an ER domain are indicated with a star (*)

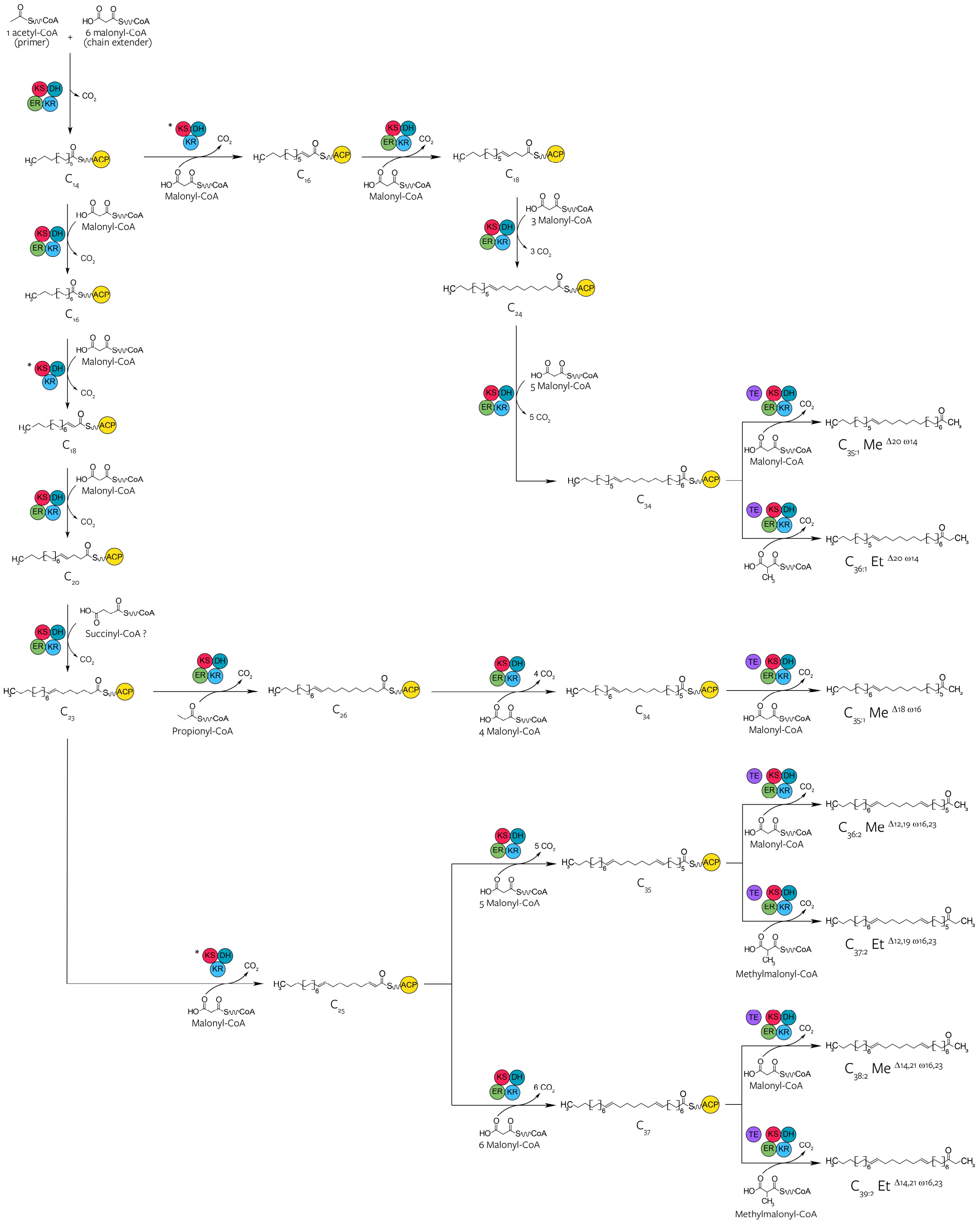

Figure S9. Proposed pathway for the synthesis of C35:1 Me Δ^{20}, C36:1 Et Δ^{20}, C35:1 Me Δ^{18}, C36:1 Et Δ^{20}, C36:2Me Δ^{11,19} , C37:2 Me Δ^{12,19}, C38:2 Me Δ^{14,21}, and C39:2 Et Δ^{14,21} LCAs using Acetyl-CoA as starter unit. Modules lacking an ER domain are indicated with a star (*)

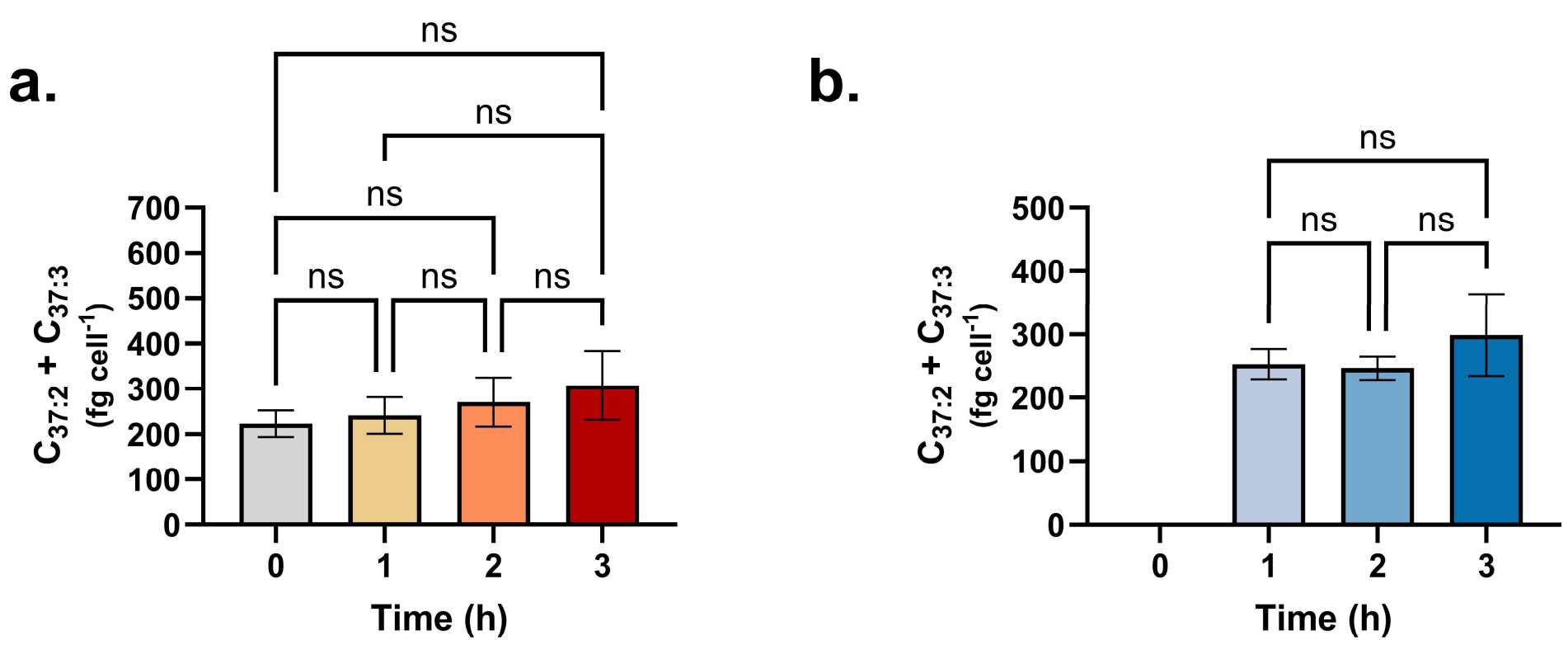

Figure S10. $C_{37:2}$ +$C_{37:3}$ LCA abundances per cell in femtograms (fg) in *G. huxleyi* strain CCMP1516 grown at 20°C **(a)** and then transferred to 10°C **(b)** X axis indicates the number of hours after cold shock. Bars indicate standard deviations across three replicates. Significant differences across time points for which transcriptomic data was also available were tested using a one-way ANOVA. ns, adjusted p-value (p_{adj}) > 0.05; * , $p_{adj} \leq 0.05$; **, $p_{adj} \leq 0.01$; *** , $p_{adj} \leq 0.001$; ****, $p_{adj} \leq 0.0001$.

a.

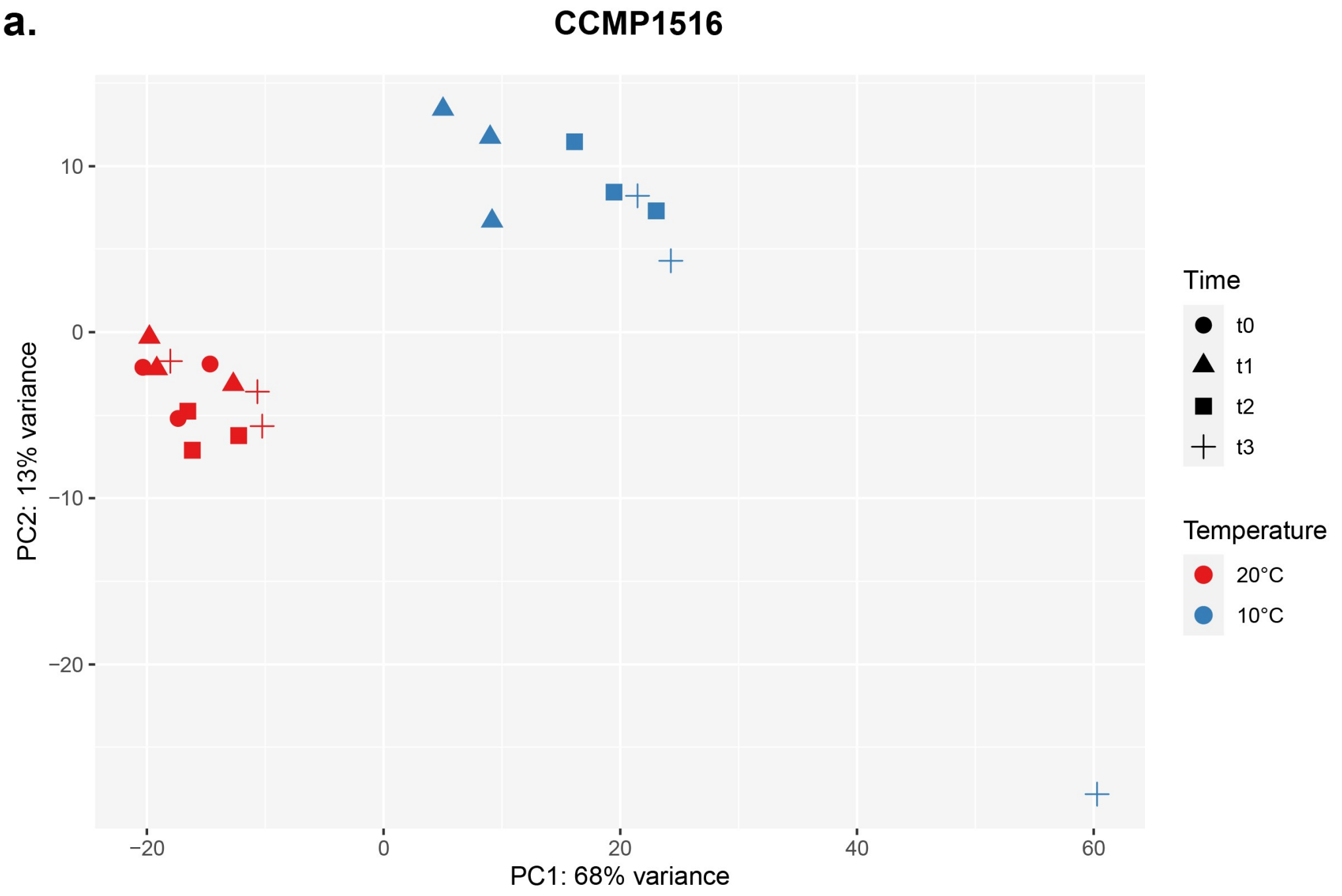

b.

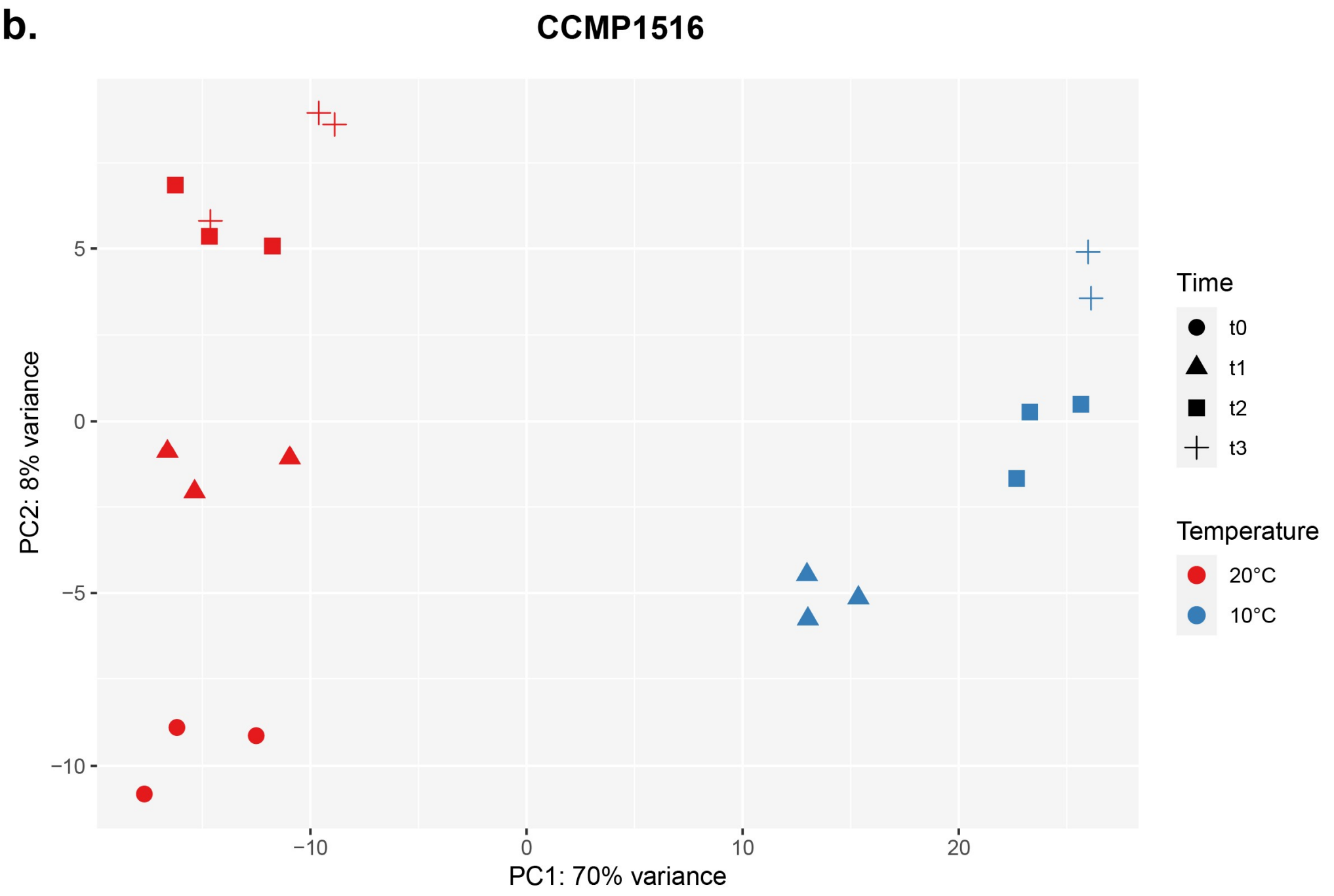

Figure S11. Principal component analysis (PCA) of transcriptome profiles. **(a)** CCMP1516 (all samples), **(b)** CCMP1516 (outlier removed). Analyses were performed using variance-stabilized transformed (vst) counts from DESeq2 and visualized using ggplot2 [69].

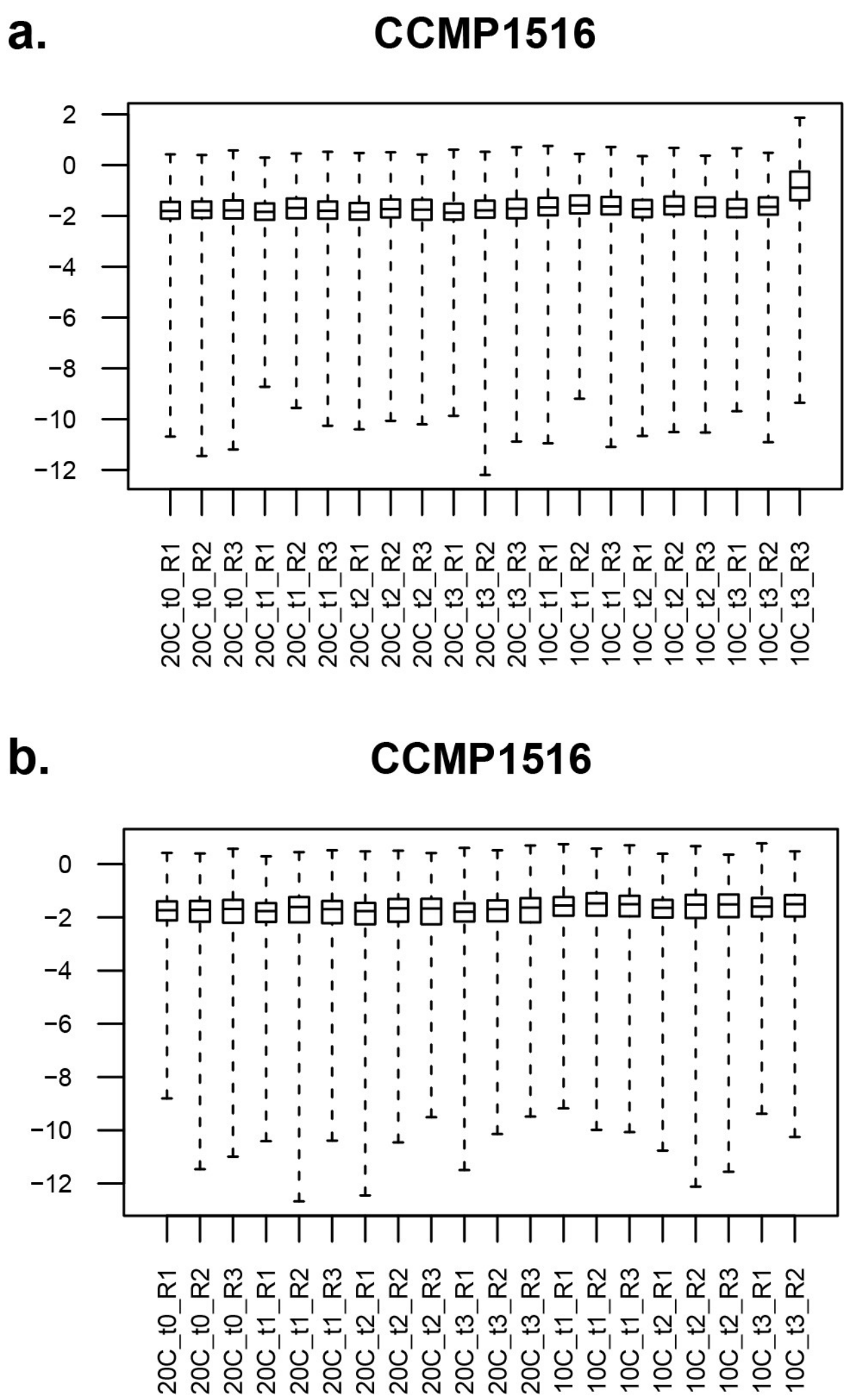

Figure S12. Cook's distance of transcriptome profiles used to identify outliers. Rows within a sample that have an extreme count outlier will have the p-value and adjusted p-value set to NA, and can be detected using Cook's distance. **(a)** CCMP1516 (all samples), **(b)** CCMP1516 (outlier removed).

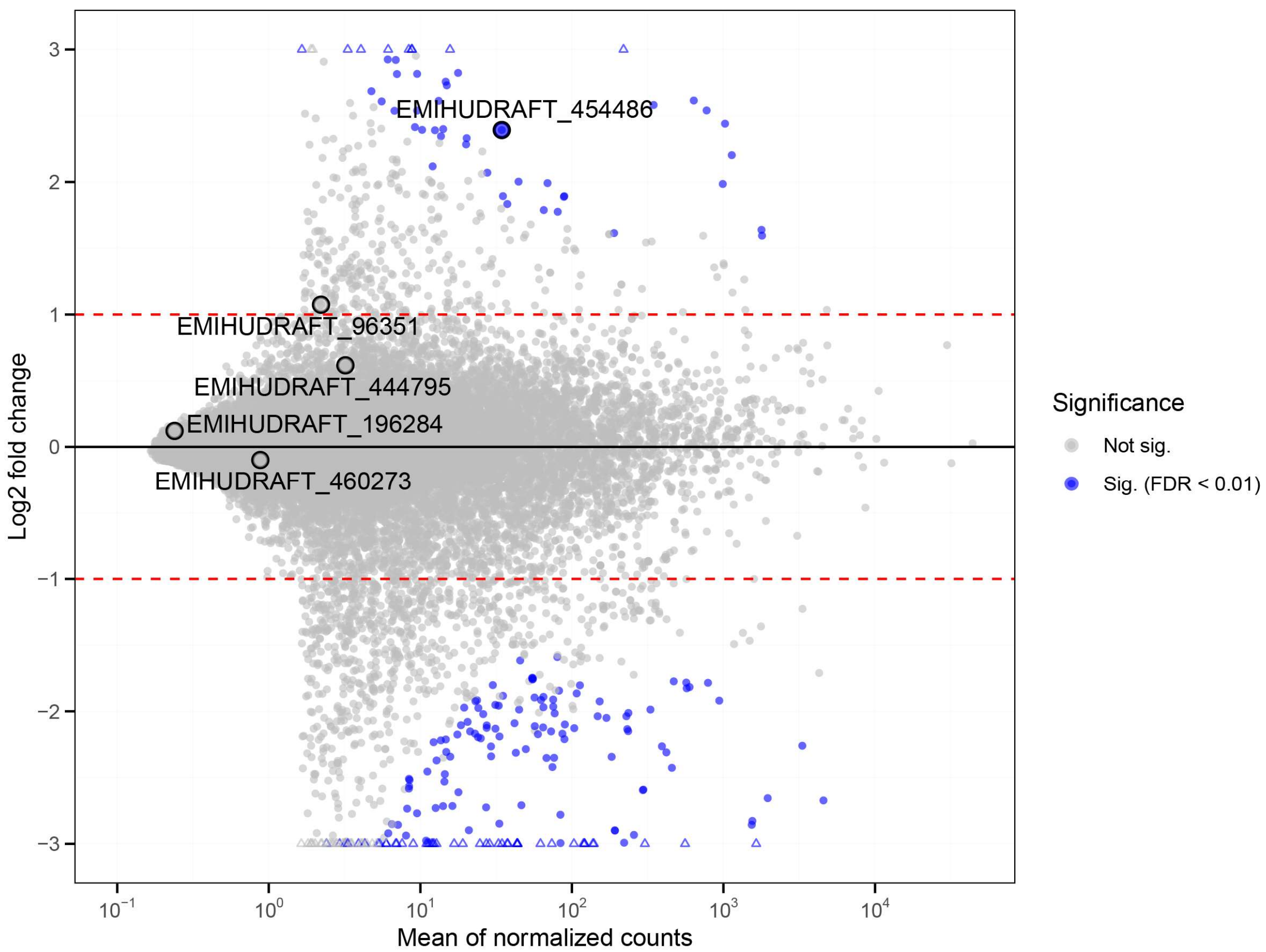

Figure S13. CCMP1516 MA-plot of differential gene expression across all time points comparing cold-shocked (10 °C) and control (20°C) cultures. Y axis indicates the log2 fold changes (LFC) over the mean of normalized counts (x axis). Genes with an adjusted p-value ≤0.01 and a shrunken LFC of < -1 or >1 generated with apeglm [70] are indicated in blue. which fall out of the window are plotted as open triangles. Gene labels correspond to genes identified as AKD1 homologs (Table S12). Plot generated using ggplot2 [69].

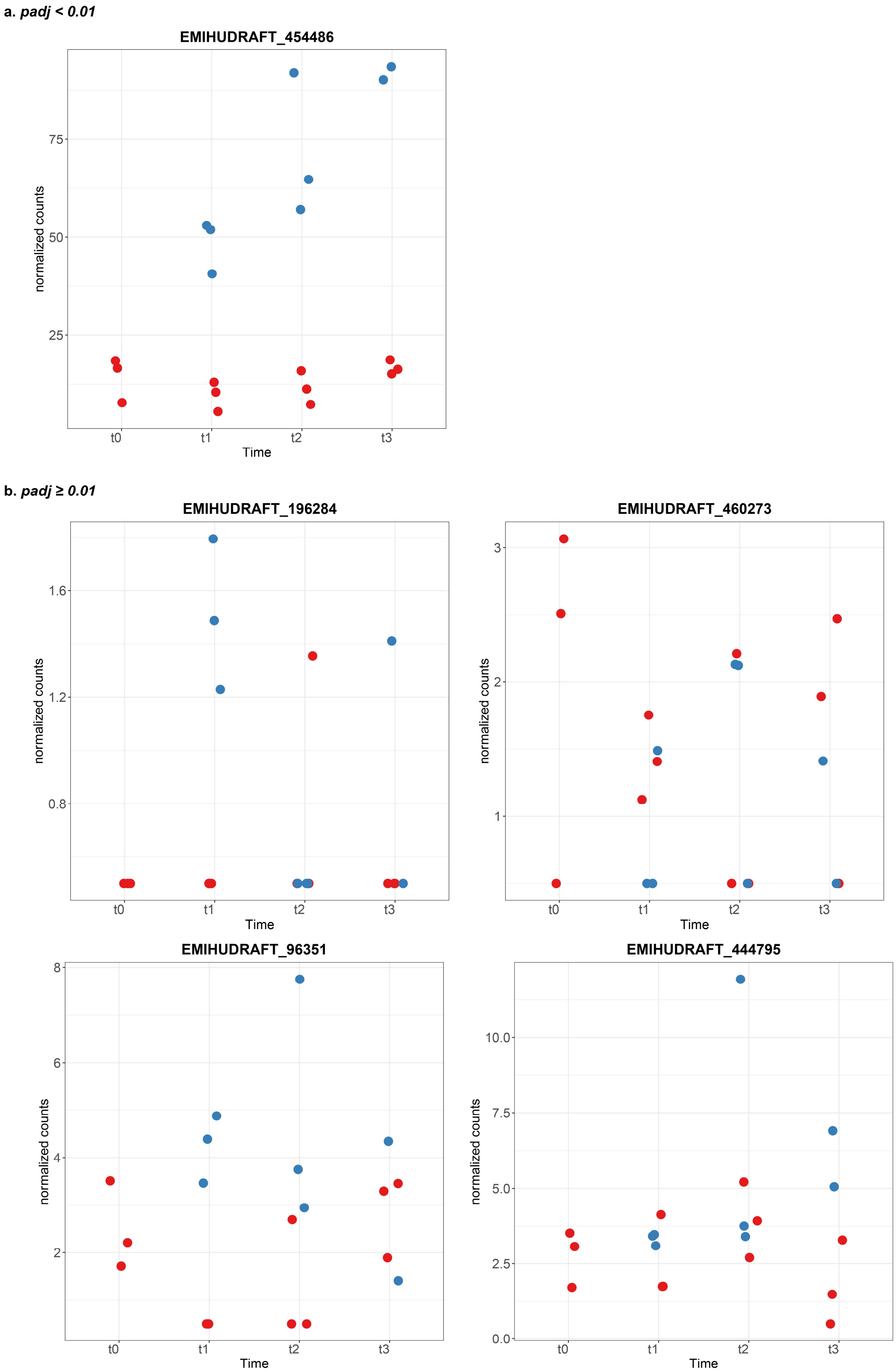

Figure S14. Normalised read counts of CCMP1516 genes orthologous to AKD1.

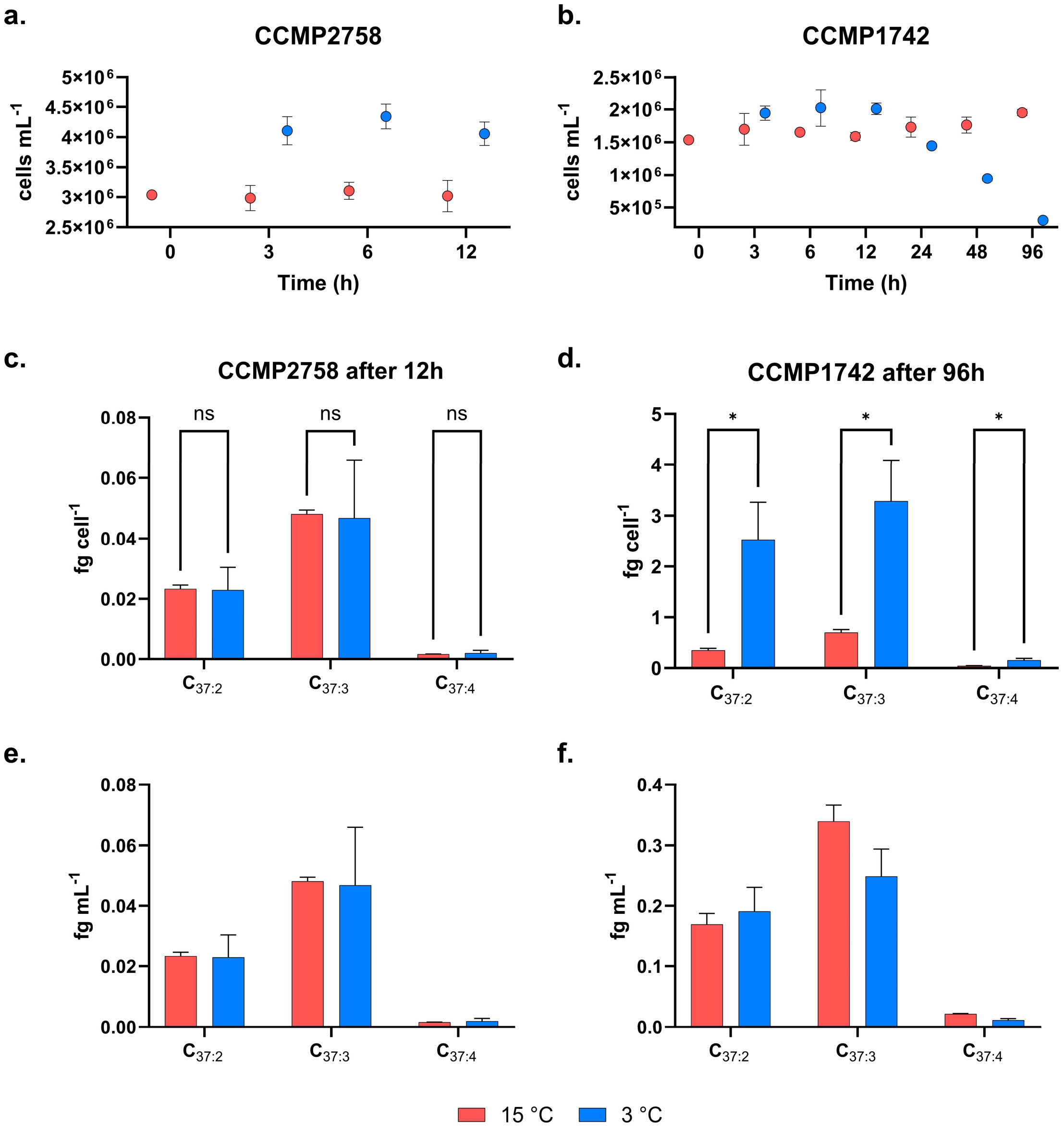

Figure S15. Cell abundances **(a and b)** and concentrations of di-, tri- and tetra-unsaturated C_{37} LCAs per cell **(c and d)** and per mL (**e and f**) during cold-shock experiments carried out in *G. huxleyi* strains grown at 15°C (red) and transferred to 3 °C (blue) for a) 12h (CCMP 2758) and b) 96h (CCMP1742) grown at 15°C and transferred to 3°C for 12h, and b) CCMP 1742 for 96h. In panels a and b X axis indicates time in hours after the cold shock. Significant differences in the $C_{37:3}/C_{37:2}$ alkenones ratio for strain CCMP 1516 were tested using multiple unpaired t-tests and the Šídák method. ns, adjusted p-value (p_{adj}) > 0.05; * , $p_{adj} \leq 0.05$; **, $p_{adj} \leq 0.01$; ***, $p_{adj} \leq 0.001$; ****, $p_{adj} \leq 0.0001$. Capped bars indicate standard deviations of triplicate cultures.

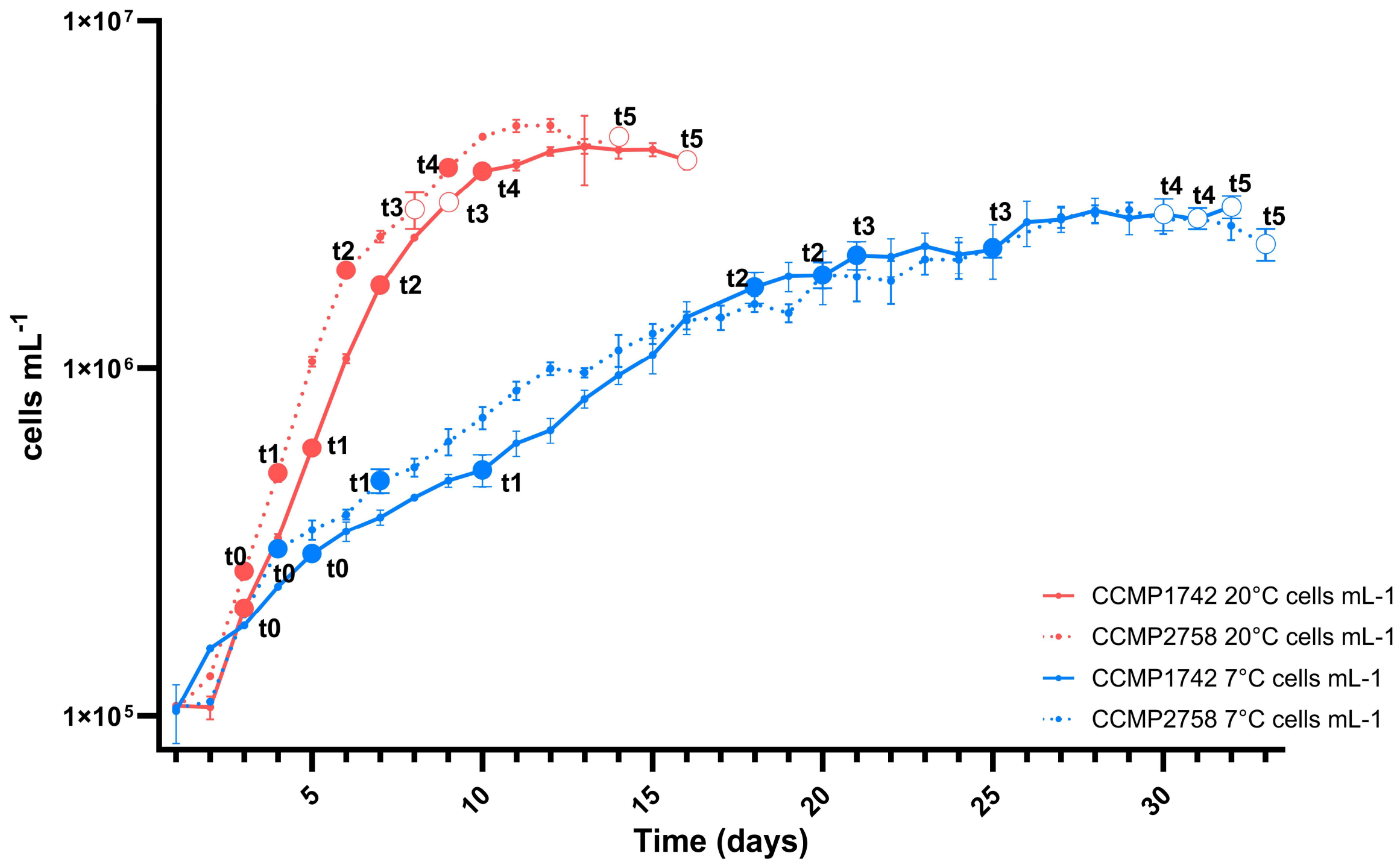

Figure S16. Cell abundances of G. huxleyi strains CCMP1742 (red) and CCMP2758 (blue) grown at 20°C (top) and 7°C (bottom). Large solid circles indicate sampling points (t0-t5) used for lipid and RNA extraction. Large empty circles indicate sampling points used only for lipid analysis. Note the difference in the sampling times based on the cell density. Capped bars indicate standard deviations of triplicate cultures. Where applicable, technical replicates (repeated dilutions of the same sample) were included in the variability calculation.

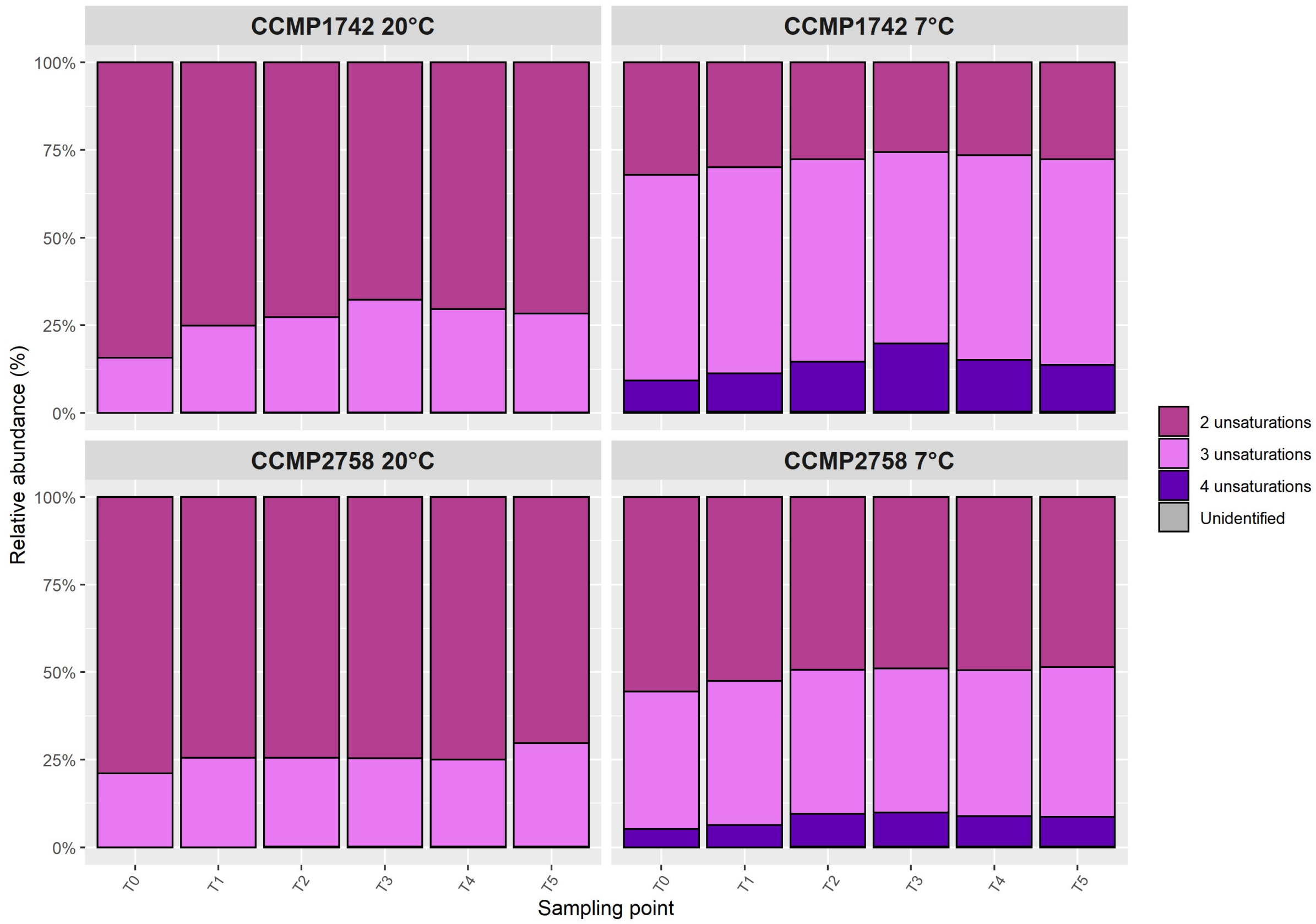

Figure S17. Relative abundance of di-, tri- and tetra -unsaturated LCAEs produced by CCMP1742 and CCMP2758 grown at 20°C and 7°C.

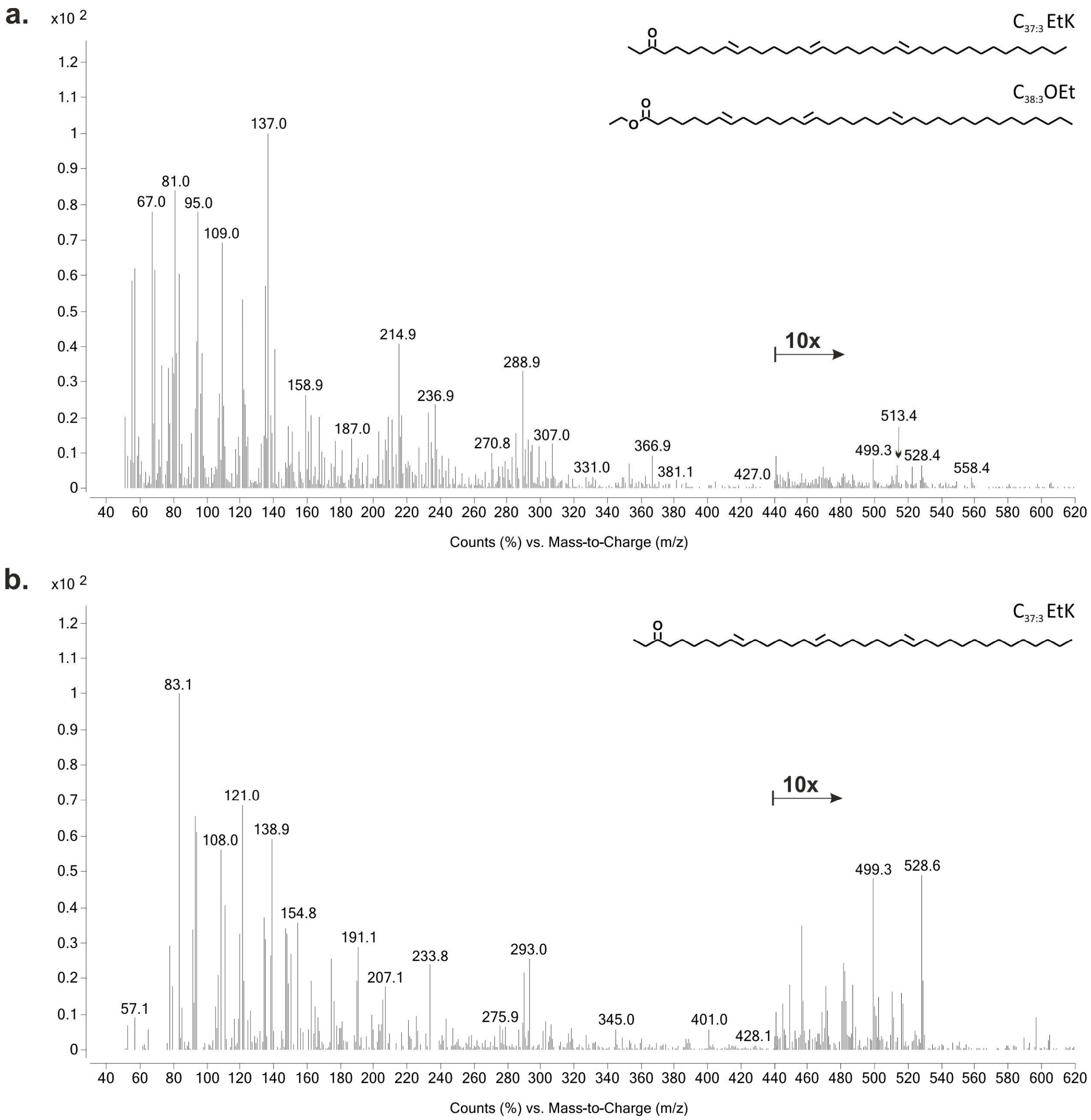

Figure S18. Mass spectra of the peak identified as a mixture of $C_{37:3}$EtK LCA and $C_{38:3}$OEt (top) and spectra of the peak located at the same retention time after base hydrolysis and identified as $C_{37:3}$EtK LCA (bottom).

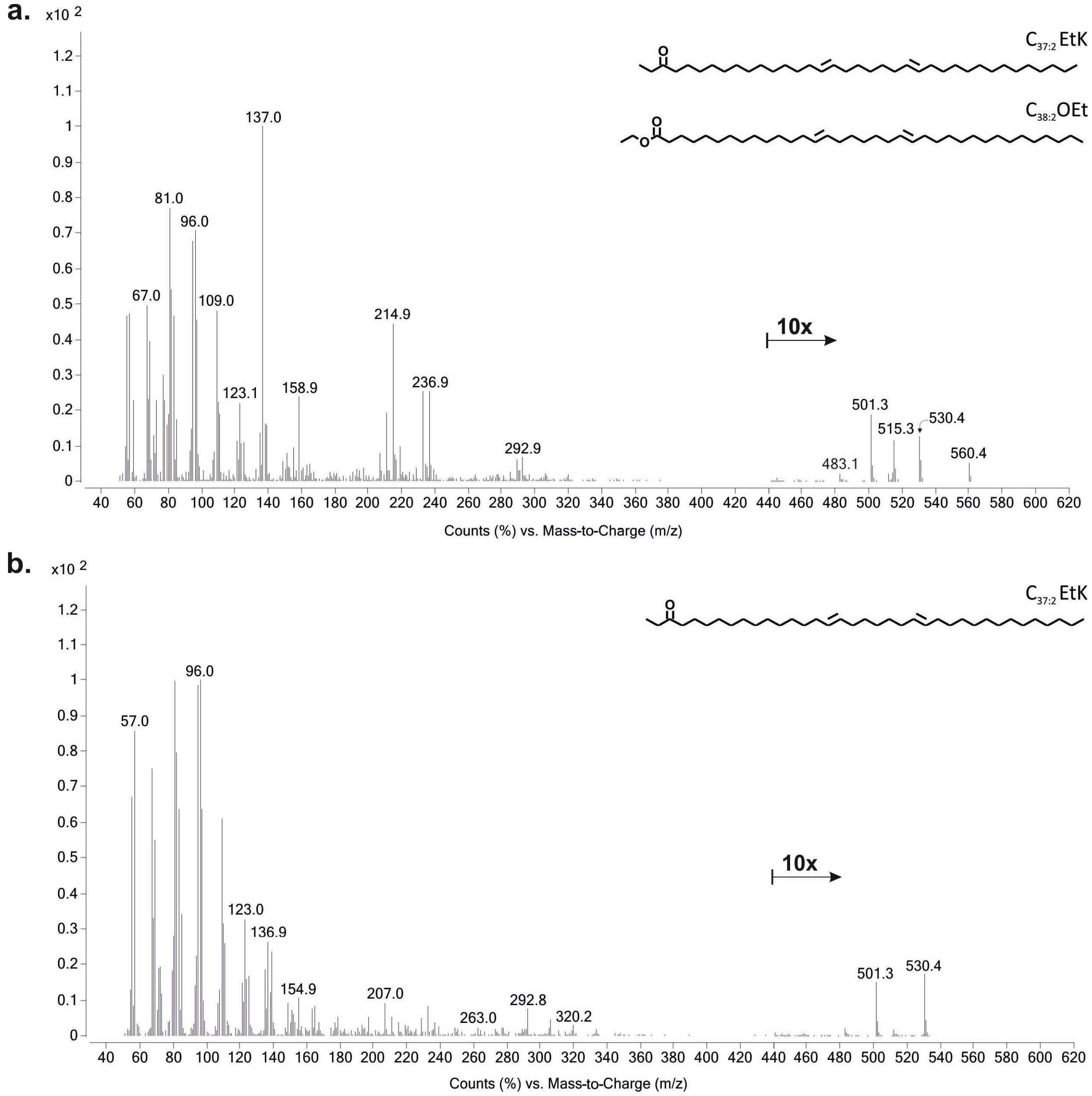

Figure S19. Mass spectra of the peak identified as a mixture of $C_{37:2}$EtK LCA and $C_{36:2}$OEt LCE (top) and spectra of the peak located at the same retention time after base hydrolysis and identified as $C_{37:2}$EtK LCA.

a.

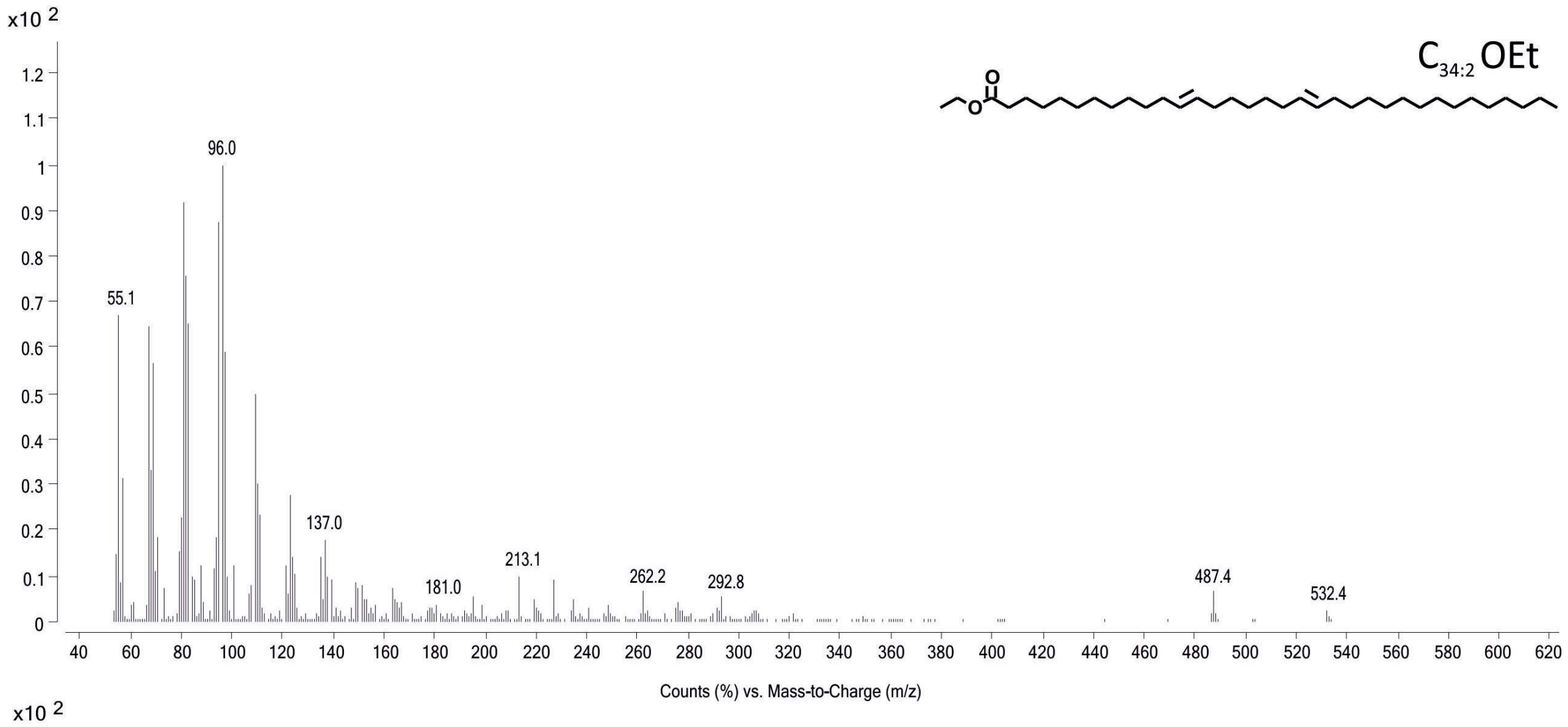

b.

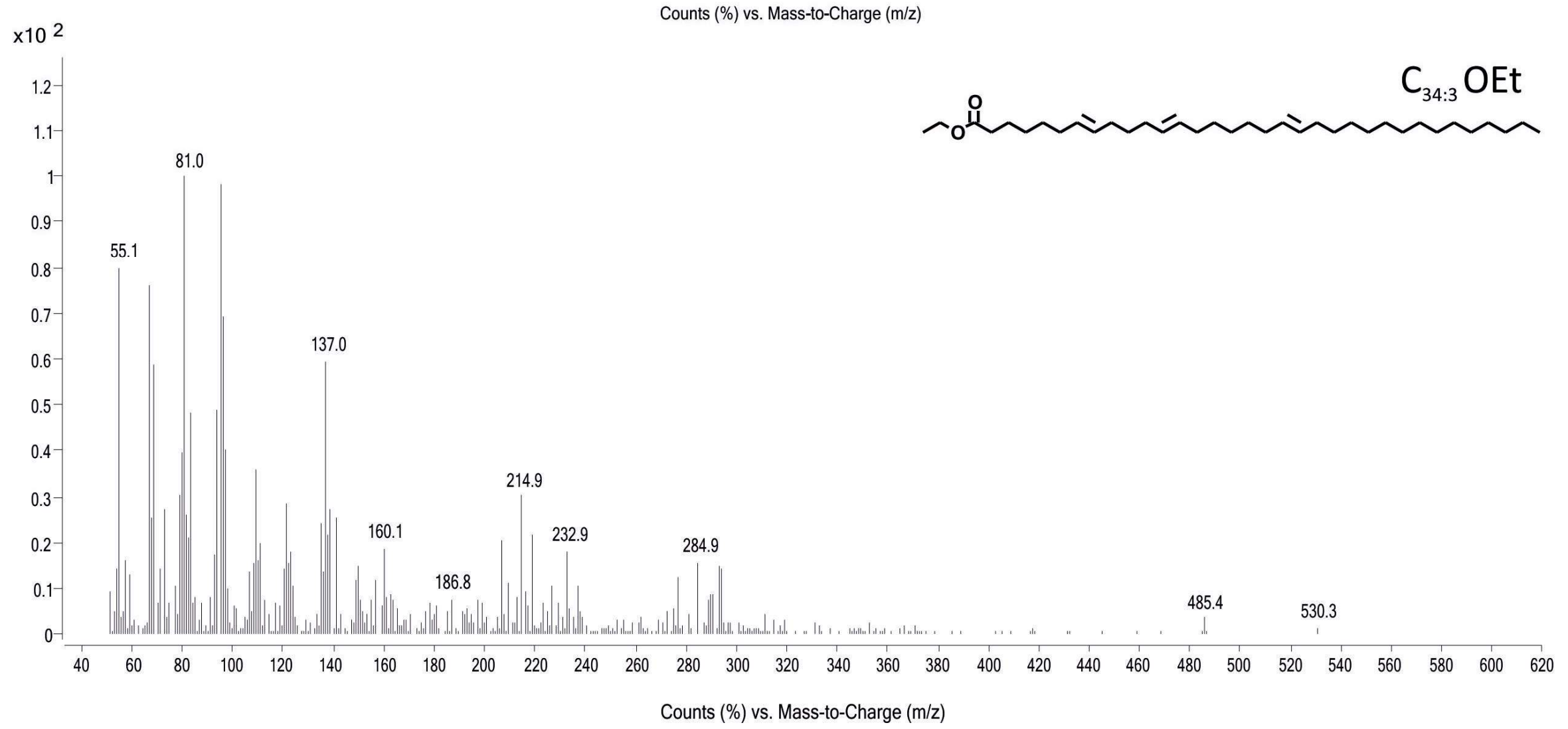

Figure S20. Mass spectra of the peaks identified as $C_{34:2}$OEt (top) and $C_{34:3}$OEt LCEs (bottom).

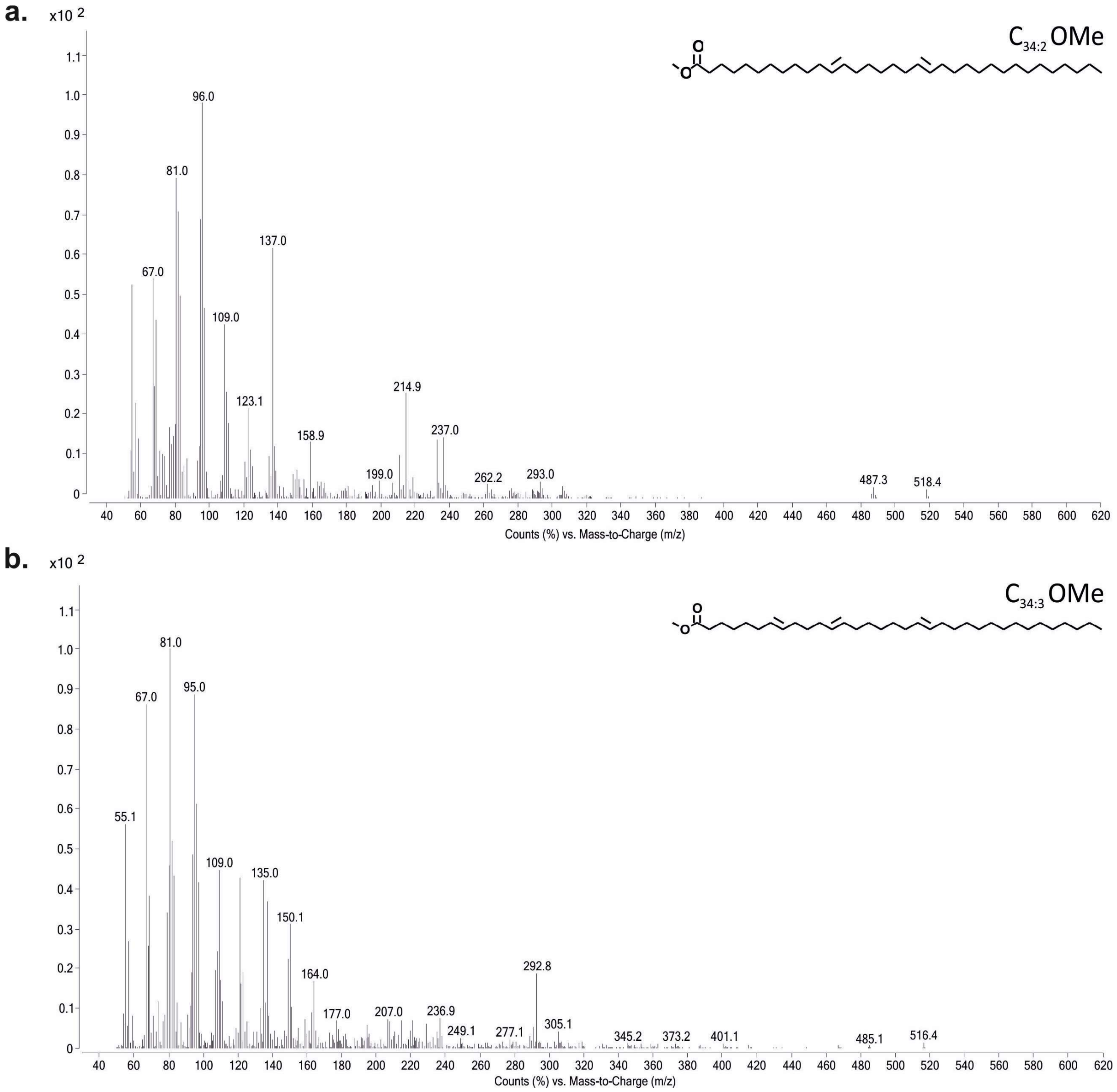

Figure S21. Mass spectra of the peaks identified as $C_{34:2}$OMe (top) and $C_{34:3}$OMe LCEs (bottom).

Total C	Main chain C	Long chain esters	Total C	Main chain C	Long chain alkenones
35	34	$C_{34:2}$OMe ($\Delta^{12,19}$)<br>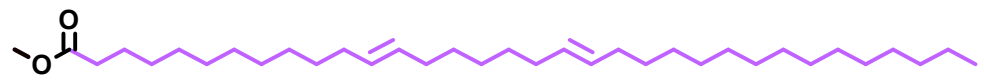<br>$C_{34:2}$OMe ($\Delta^{7,12,19}$)<br>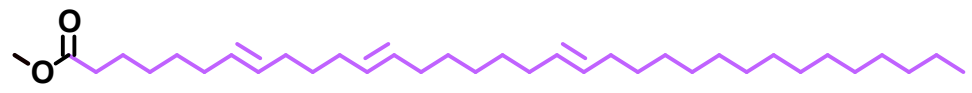	35	34	$C_{35:2}$Me ($\Delta^{12,19}$)<br>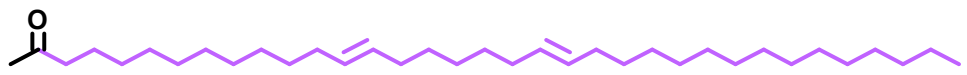<br>$C_{35:2}$Me ($\Delta^{7,12,19}$)<br>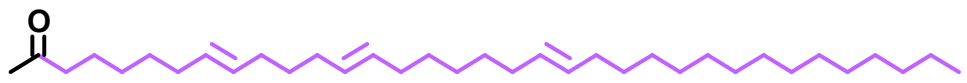
36	34	$C_{34:2}$OEt ($\Delta^{12,19}$)<br>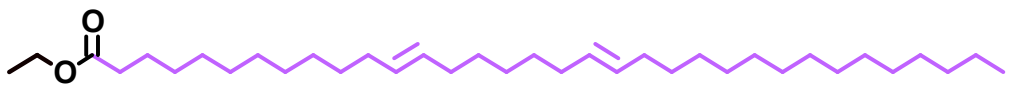<br>$C_{34:3}$OEt ($\Delta^{7,12,19}$)<br>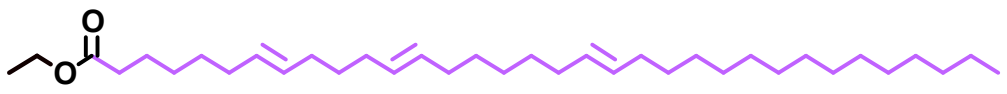	36	34	$C_{36:2}$Et ($\Delta^{12,19}$)<br>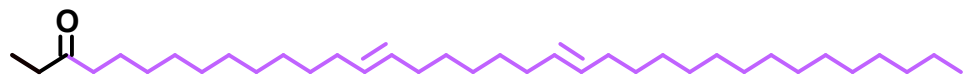<br>$C_{36:3}$Et ($\Delta^{7,12,19}$)<br>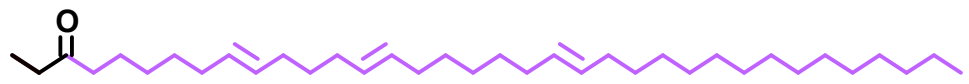
			36	35	$C_{36:2}$Me ($\Delta^{14,21}$)<br>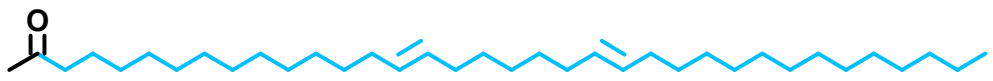<br>$C_{36:3}$Me ($\Delta^{7,14,21}$)<br>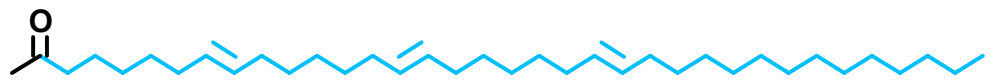
			37	35	$C_{37:2}$Et ($\Delta^{14,21}$)<br>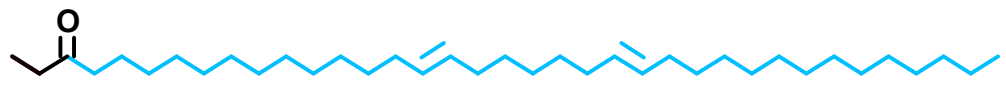<br>$C_{37:3}$Et ($\Delta^{7,14,21}$)<br>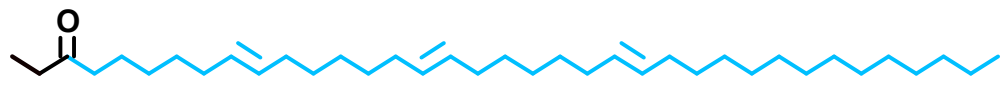
37	36	$C_{36:2}$OMe ($\Delta^{14,21}$)<br>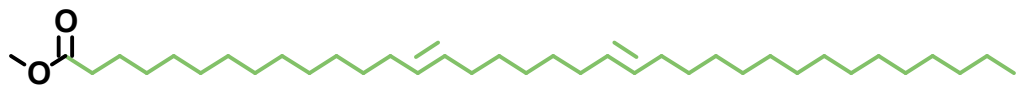<br>$C_{36:3a}$OMe ($\Delta^{7,14,21}$)<br>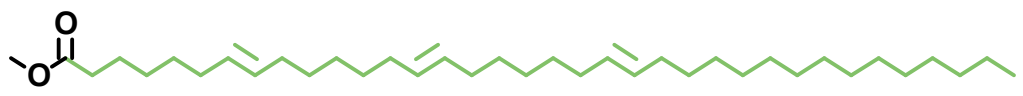<br>$C_{36:3b}$OMe ($\Delta^{14,21,28}$)<br>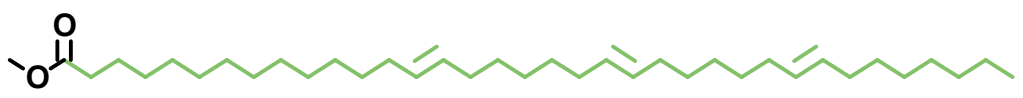<br>$C_{36:4}$OMe ($\Delta^{7,14,21,28}$)<br>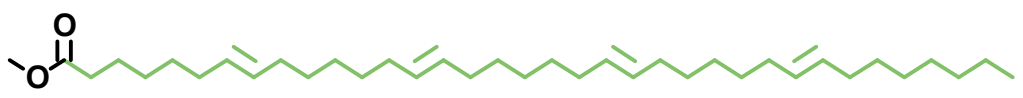	37	36	$C_{37:2}$Et ($\Delta^{14,21}$)<br>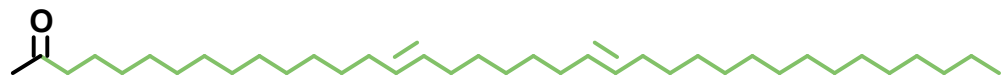<br>$C_{37:3a}$Et ($\Delta^{7,14,21}$)<br>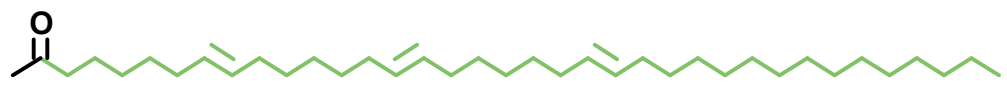<br>$C_{37:3b}$Et ($\Delta^{14,21,28}$)<br>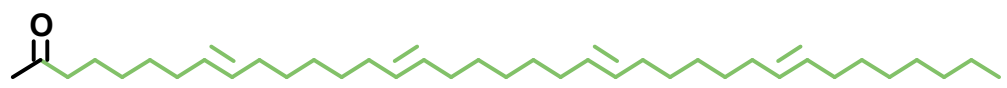<br>$C_{37:4}$Et ($\Delta^{7,14,21,28}$)<br>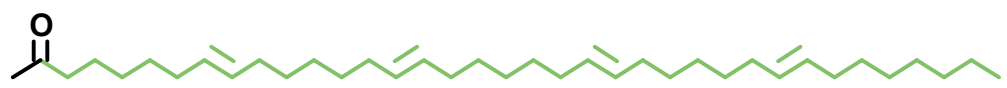
38	36	$C_{36:2}$OEt ($\Delta^{14,21}$)<br>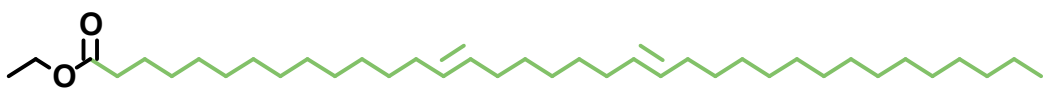<br>$C_{36:3a}$OEt ($\Delta^{7,14,21}$)<br>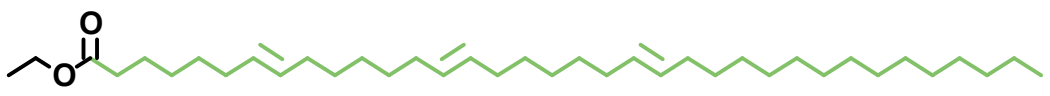<br>$C_{36:3b}$OEt ($\Delta^{14,21,28}$)<br>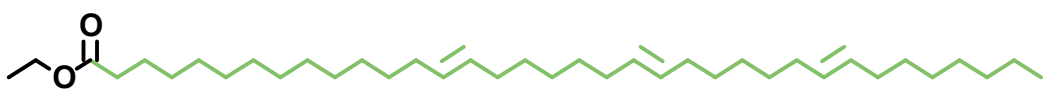	38	36	$C_{38:2}$Et ($\Delta^{14,21}$)<br>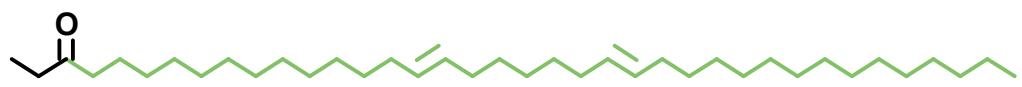<br>$C_{38:3a}$Et ($\Delta^{7,14,21}$)<br>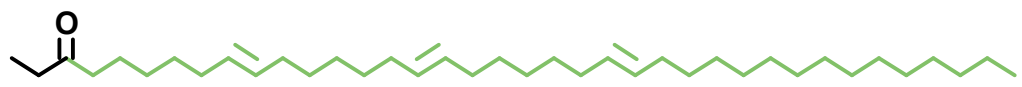<br>$C_{38:3b}$Et ($\Delta^{14,21,28}$)<br>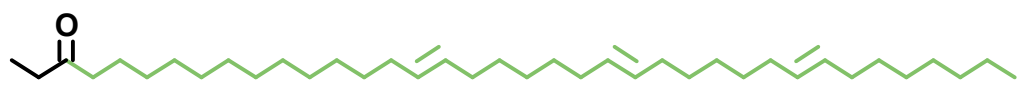<br>$C_{38:4}$Et ($\Delta^{7,14,21,28}$)<br>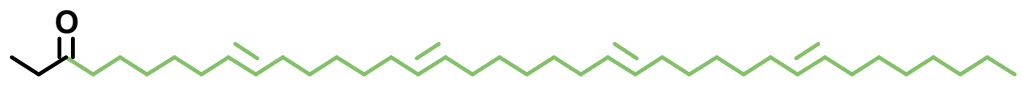

Figure S22. Molecular structures of all LCEs (left) and LCAs (right) identified in this study. The "total C" column indicates the total amount of carbons in the molecule (R in Figure 1), whilst "Main chain C" indicates only the number of carbons on the right side of the keto group (R' in Figure 1).

Total C	Main chain C	Long chain esters	Total C	Main chain C	Long chain alkenones
			38	37	$C_{38:2}$Me ($\Delta^{14,21}$)<br>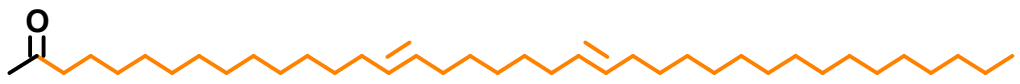<br>$C_{38:3a}$Me ($\Delta^{7,14,21}$)<br>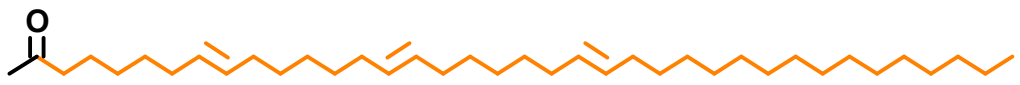<br>$C_{38:3b}$Me ($\Delta^{14,21,28}$)<br>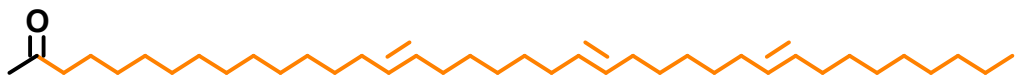<br>$C_{38:4}$Me ($\Delta^{7,14,21,28}$)<br>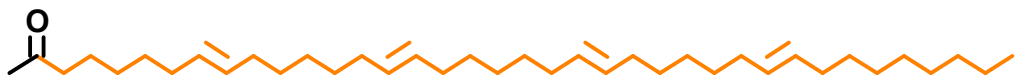
			38	37	$C_{38:2}$Me ($\Delta^{14,21}$)<br>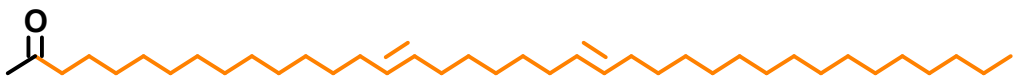<br>$C_{38:3a}$Me ($\Delta^{7,14,21}$)<br>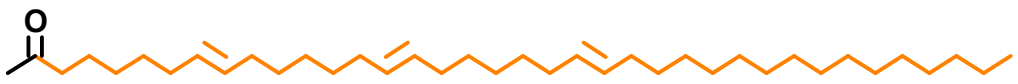<br>$C_{38:3b}$Me ($\Delta^{14,21,28}$)<br>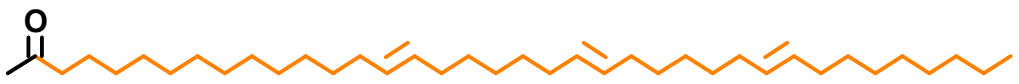<br>$C_{38:4}$Me ($\Delta^{7,14,21,28}$)<br>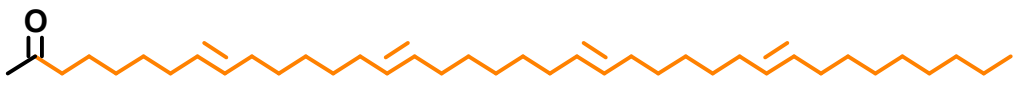
			39	37	$C_{39:2}$Et ($\Delta^{14,21}$)<br>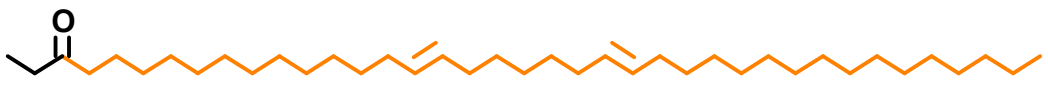<br>$C_{39:3a}$Et ($\Delta^{7,14,21}$)<br>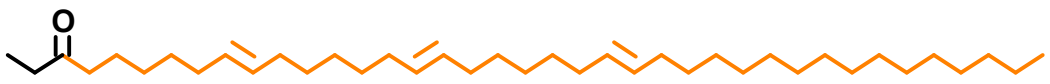<br>$C_{39:4}$Et ($\Delta^{7,14,21,28}$)<br>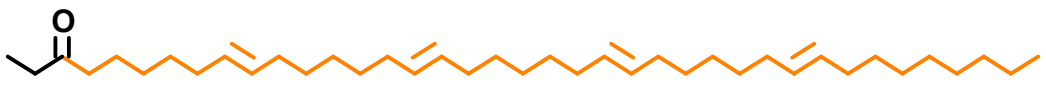
			39	38	$C_{39:2}$Me ($\Delta^{14,21}$)<br>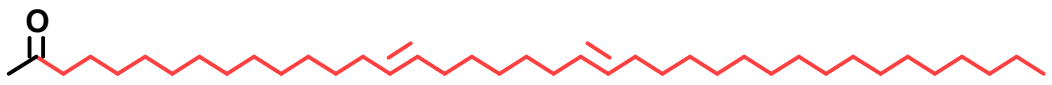<br>$C_{39:3a}$Me ($\Delta^{7,14,21}$)<br>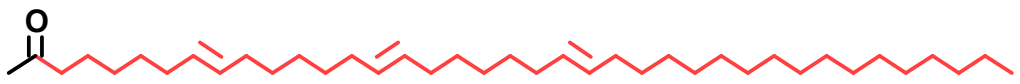
			40	38	$C_{40:2}$Et ($\Delta^{14,21}$)<br>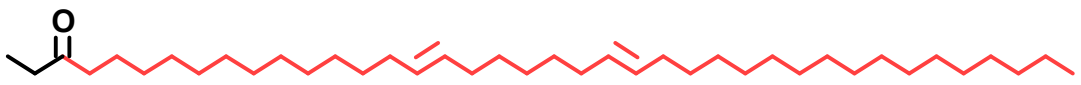<br>$C_{40:3a}$Et ($\Delta^{7,14,21}$)<br>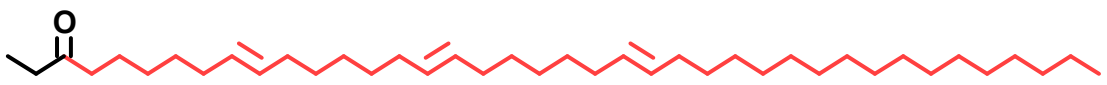

Figure S22. *(continued)*

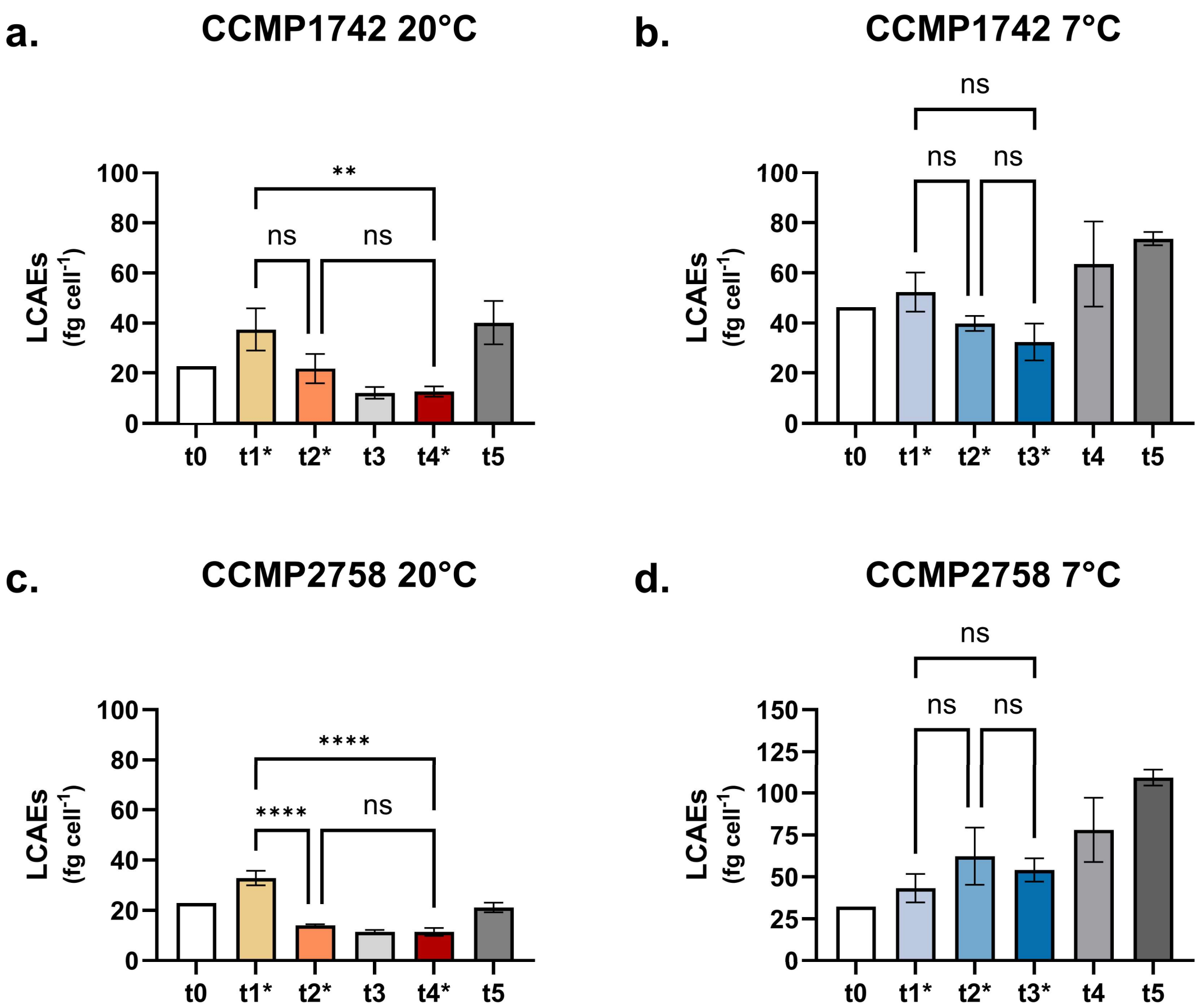

Figure S23. Long chain alkenone and ester (LCAE) abundances per cell in femtograms (fg) in G. huxleyi strains CCMP1742 **(a, b)** and CCMP2758 **(c ,d)** . grown at 20°C **(a, c)** and 7°C **(b, d)**. t0-t5 indicate the sampling points which correspond to different culturing time but similar growth stage, equivalence of the sampling points in terms of culturing time can be found in Figure S16. Asterisks (*) on sampling point labels indicate the time points for which transcriptomic data is available. Bars indicate standard deviations across three replicates, except for time zero (t0), where only one sample was taken from the main 3 liter culture before it was distributed across 15 flasks (see Material and Methods for details). Significant differences across time points for which transcriptomic data was also available were tested using an one-way ANOVA. ns, adjusted p-value (p_{adj}) > 0.05; * , $p_{adj} \leq 0.05$; **, $p_{adj} \leq 0.01$; *** , $p_{adj} \leq 0.001$; ****, $p_{adj} \leq 0.0001$.

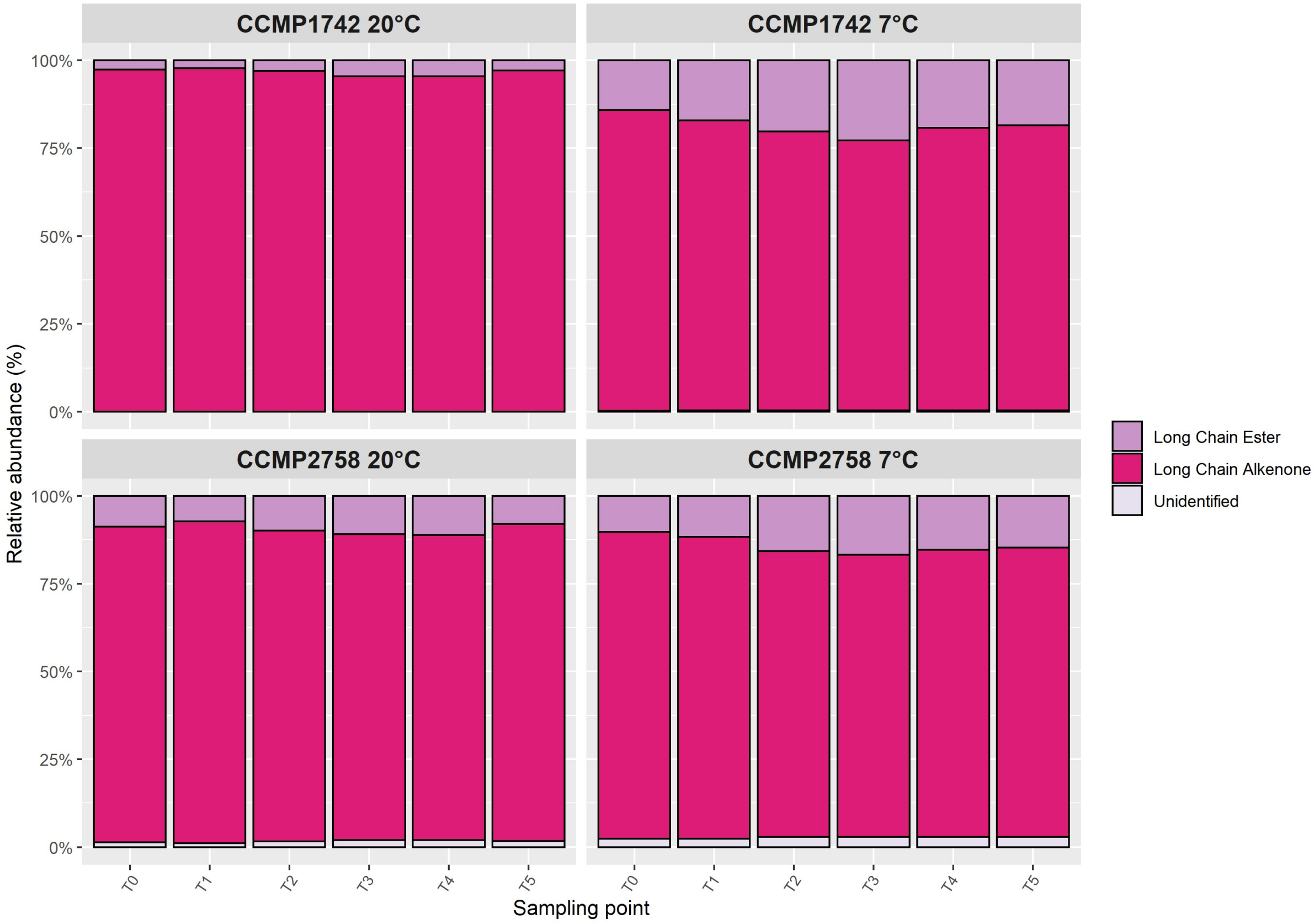

Figure S24. Relative abundance of Long Chain Esters (LCEs) and Long Chain alkenones (LCAs) produced by CCMP1742 and CCMP2758 grown at 20°C and 7°C

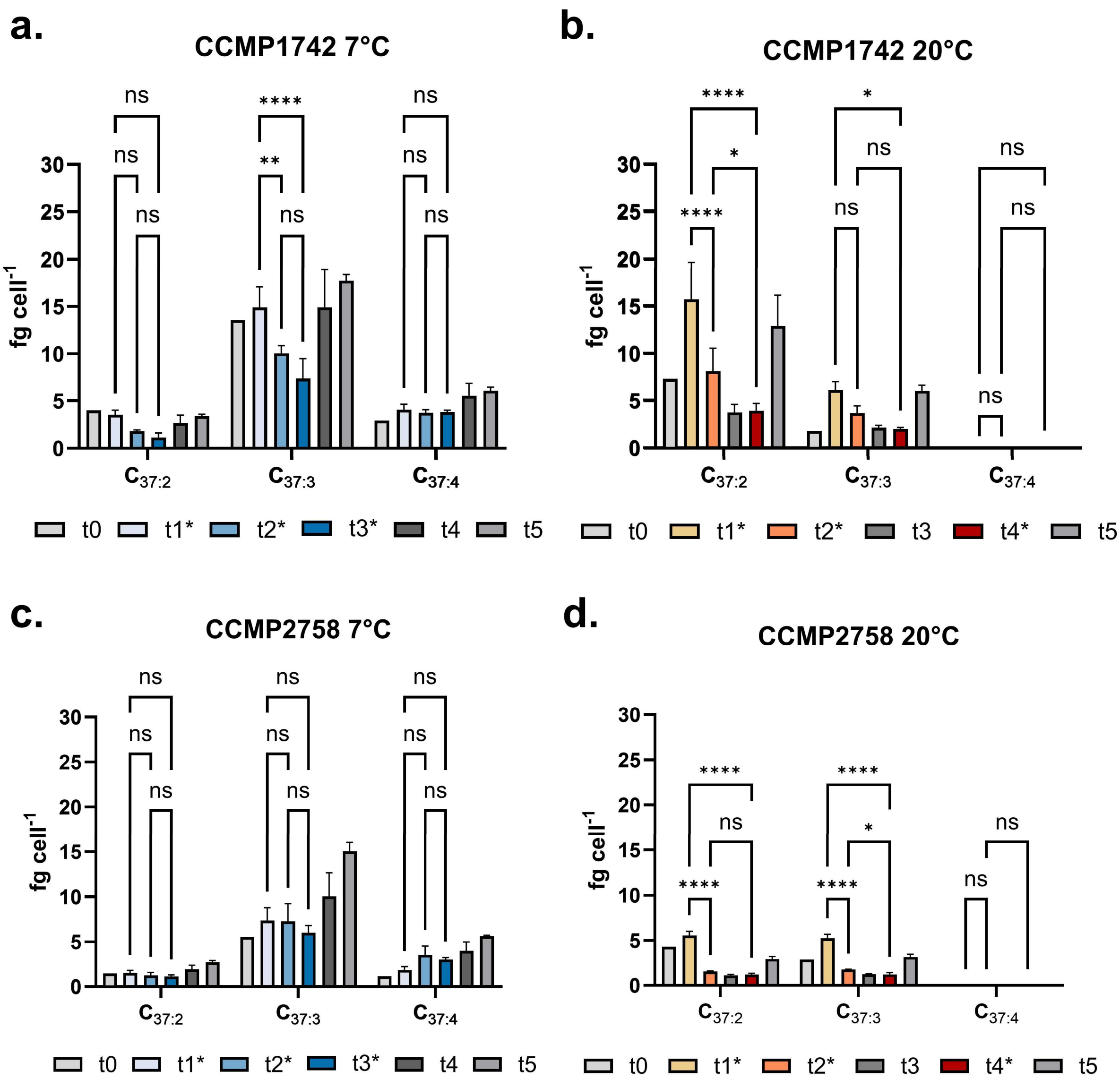

Figure S25. Cellular abundances of selected $C_{37:2}$, $C_{37:3}$, $C_{37:4}$ LCAs in *G. huxleyi* strains CCMP1742 **(a, b)** and CCMP2758 **(c, d)**, grown at 20°C **(c, e)** and 7 °C **(d, f)**. Bars indicate standard deviations across three replicate flasks, except for t0 where only one sample was taken from the main three-liter culture before it was distributed across 15 flasks. Statistical comparisons (Tukey's test) were performed only for time points where transcriptomic data was also available (indicated with a star). Significance levels: ns, adjusted p-value (p_{adj}) > 0.05; *, $p_{adj} \leq 0.05$; **, $p_{adj} \leq 0.01$; ***, $p_{adj} \leq 0.001$; ****, $p_{adj} \leq 0.0001$.

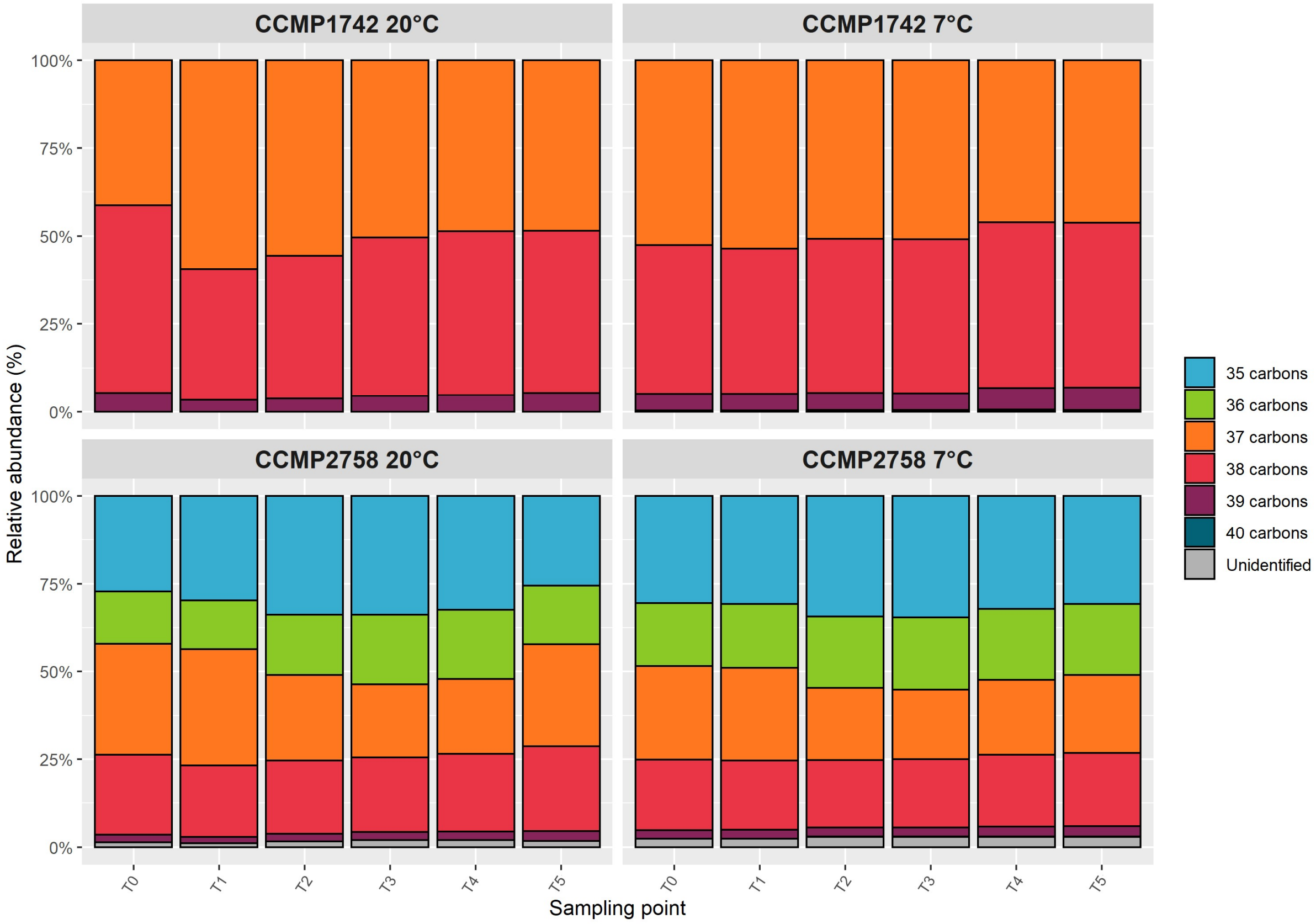

Figure S26. Relative abundance of LCAEs classified according to the total number of carbon atoms (R, i.e total compound length including the methyl and ethyl headgroups, Figure 1) produced by CCMP1742 and CCMP2758 grown at 20°C and 7°C.

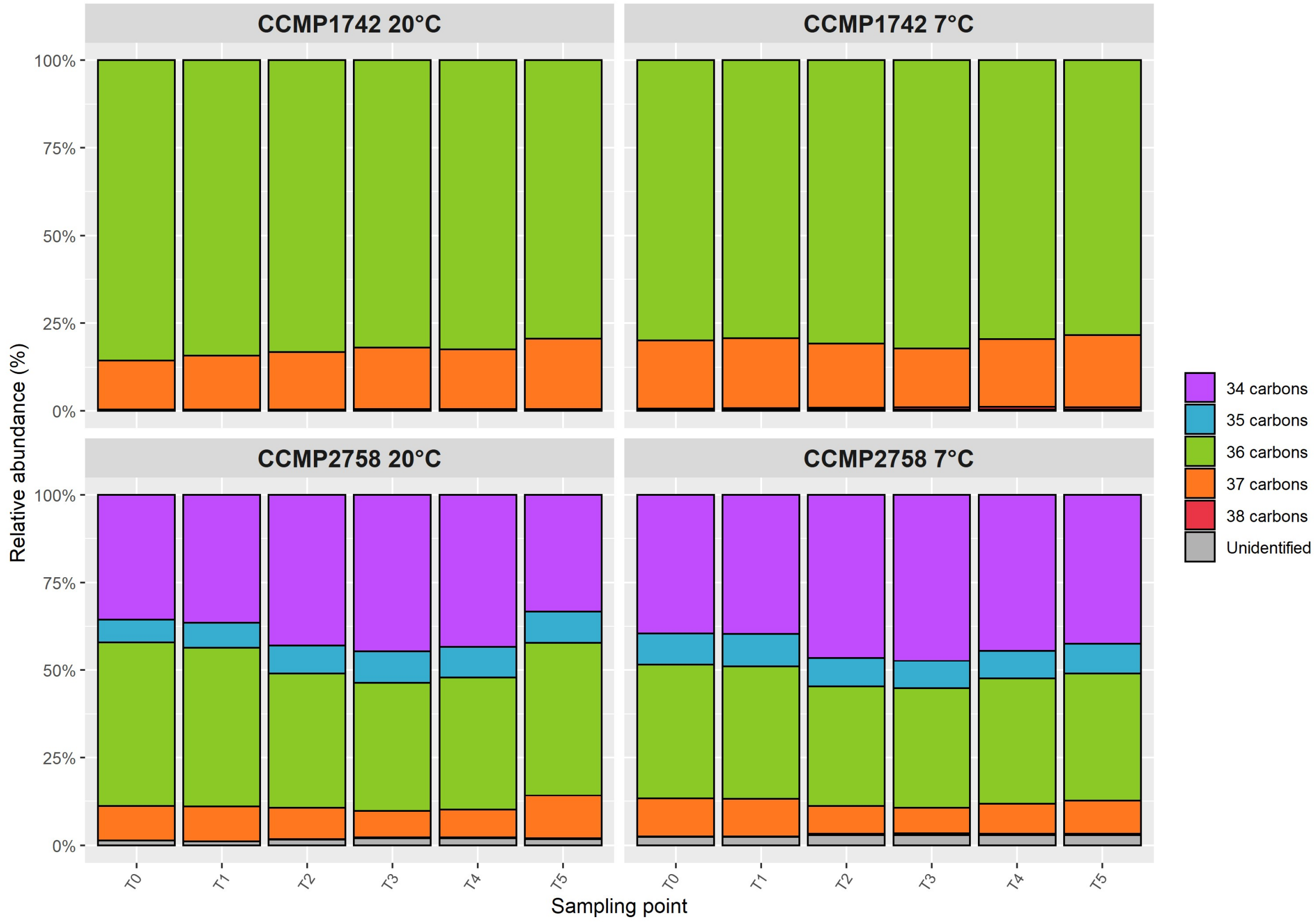

Figure S27. Relative abundance of LCAEs classified according to the number of carbon atoms contained in R' (i.e. total compound length minus the methyl and ethyl headgroups, Figure 1) produced by CCMP1742 and CCMP2758 grown at 20°C and 7°C.

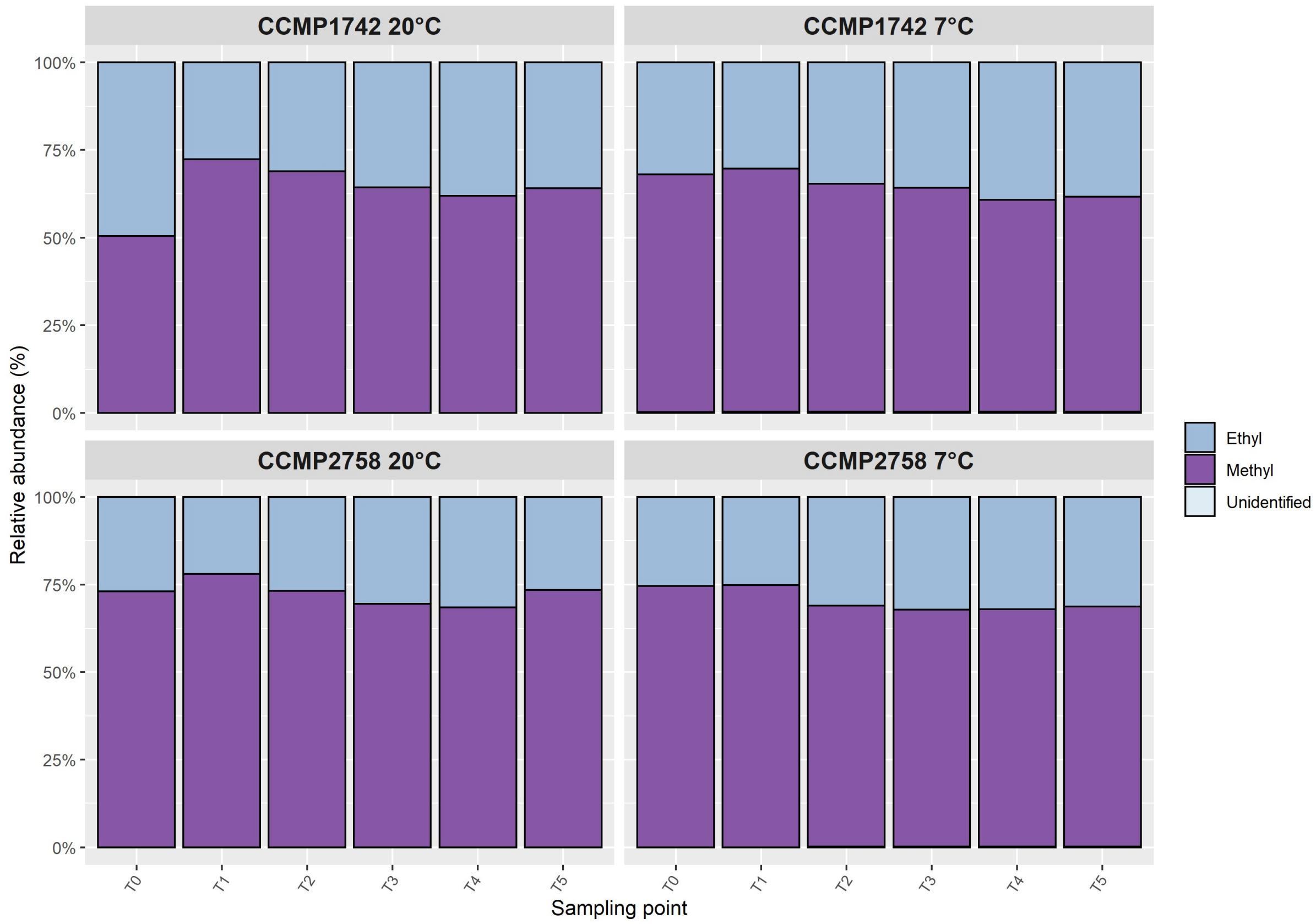

Figure S28. Relative abundances of LCAEs containing a methyl or ethyl headgroup (R'' in Figure 1) produced by CCMP1742 and CCMP2758 grown at 20°C and 7°C.

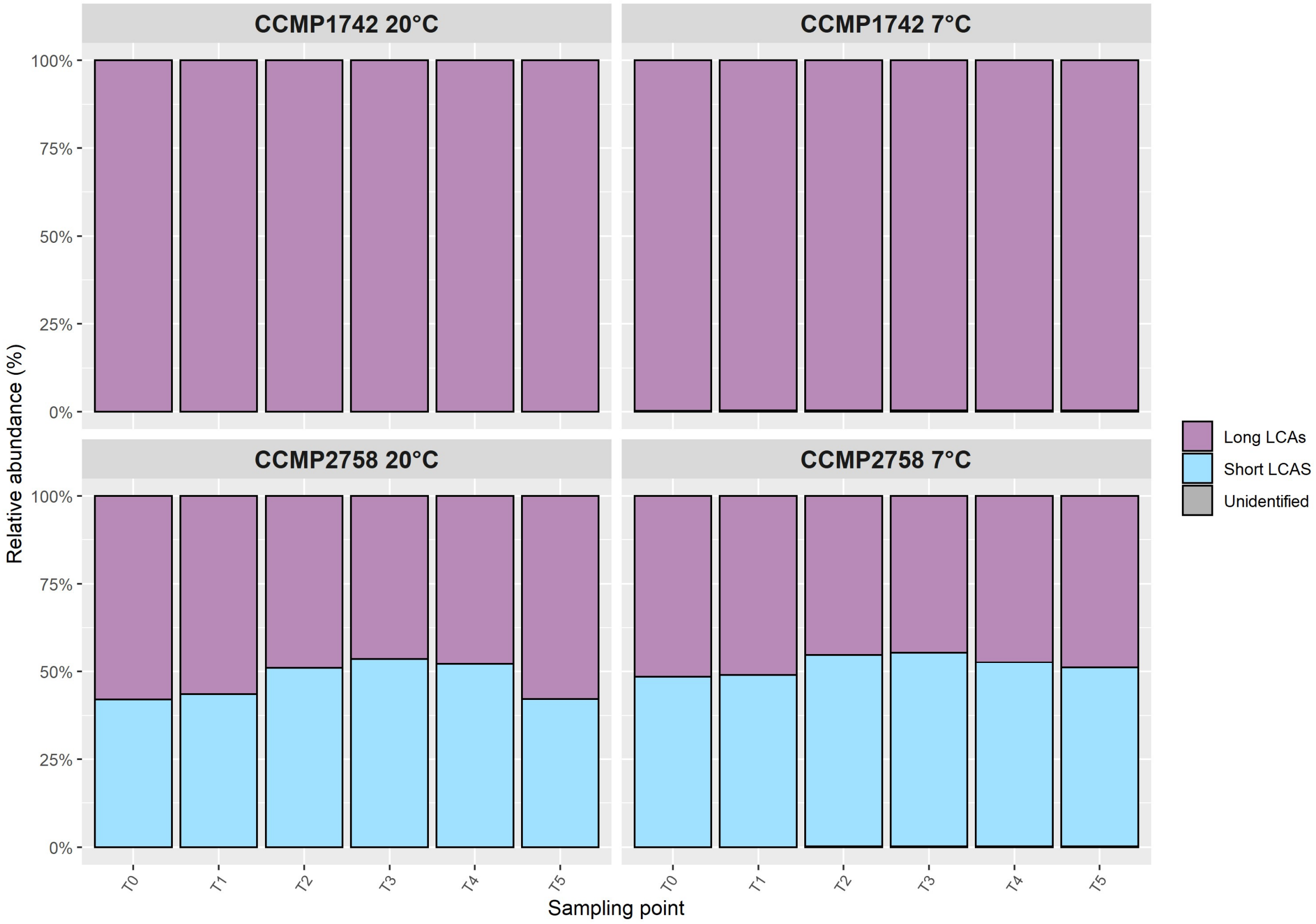

Figure S29. Relative abundance of "Long" (C_{37-40}) or "Short" LCAEs (C_{35-36}) produced by CCMP1742 and CCMP2758 grown at 20°C and 7°C.

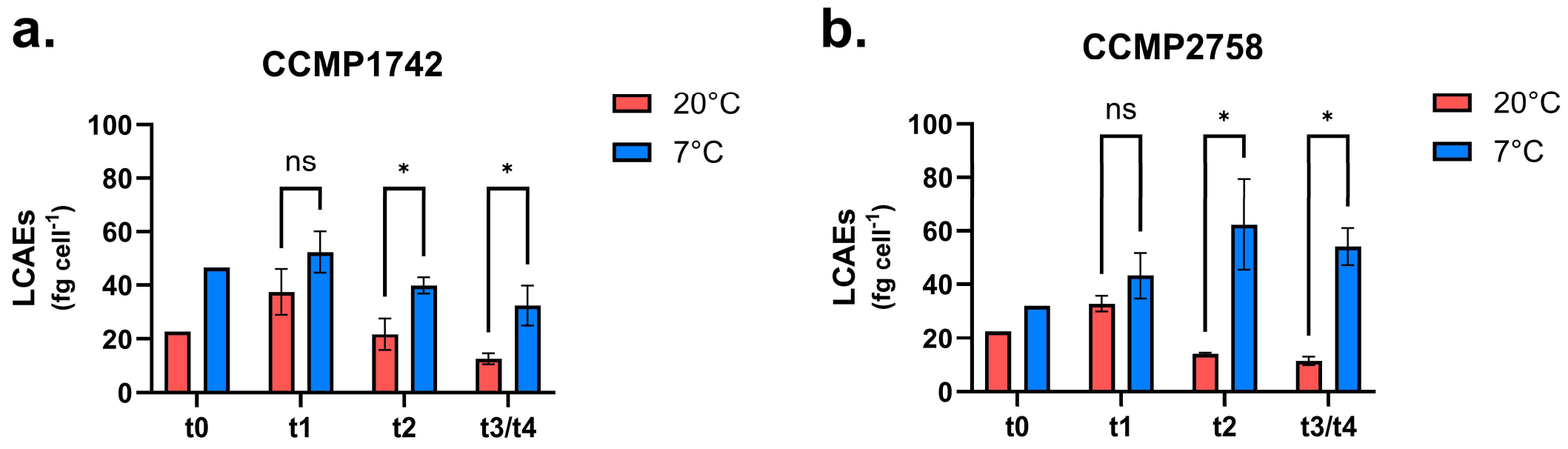

Figure S30. Total LCAE abundances in femtograms (fg) per cell in G. huxleyi strains CCMP1742 **(a)** and CCMP2758 **(b)** grown at 20°C (red bars) and 7°C (blue bars) during temperature acclimation experiments. Only time points for which transcriptomic data is also available are shown; t3 for samples collected when strains were grown at 7°C and t4 samples for samples collected when strains were grown at 20°C. Equivalence of the sampling points in terms of culturing time can be found in Figure S16. Bars indicate standard deviations across three replicates, except for time zero (t0) , where only one sample was taken from the main three liter culture before it was distributed across 15 flasks (see Material and Methods for details). Significant differences between tremperatures for all time points (except t0 for which only one measuremet was available) were tested using multiple unpaired t-tests with Holm-Šídák method. ns, p-value > 0.05; * , p-value ≤ 0.05.

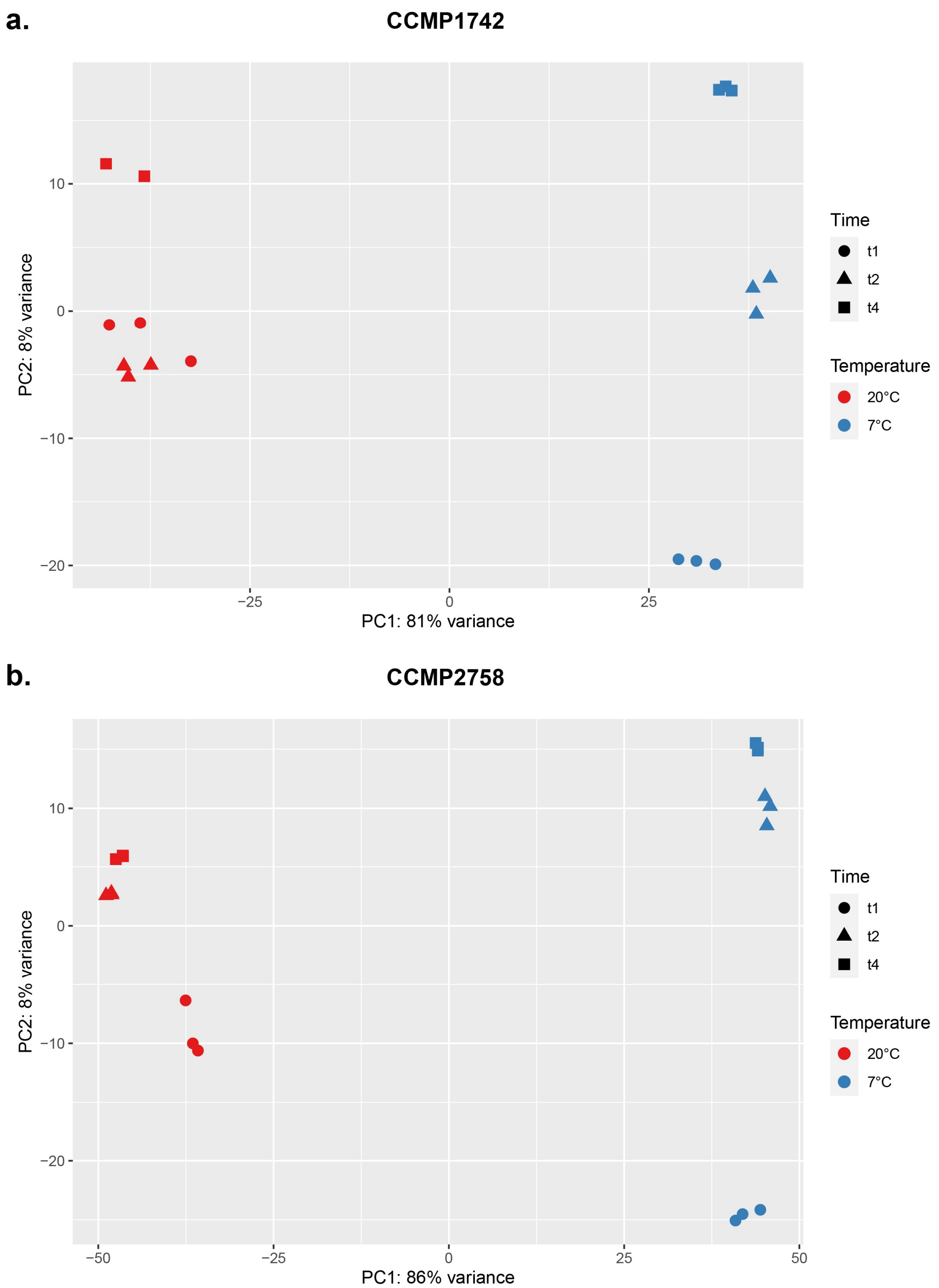

Figure S31. Principal component analysis (PCA) of transcriptome profiles. **(a)** CCMP1742 (excluding low-coverage sample), and **(b)** CCMP2758 (all samples). Analyses were performed using variance-stabilized transformed (vst) counts from DESeq2 and visualized using ggplot2 [69].

a.

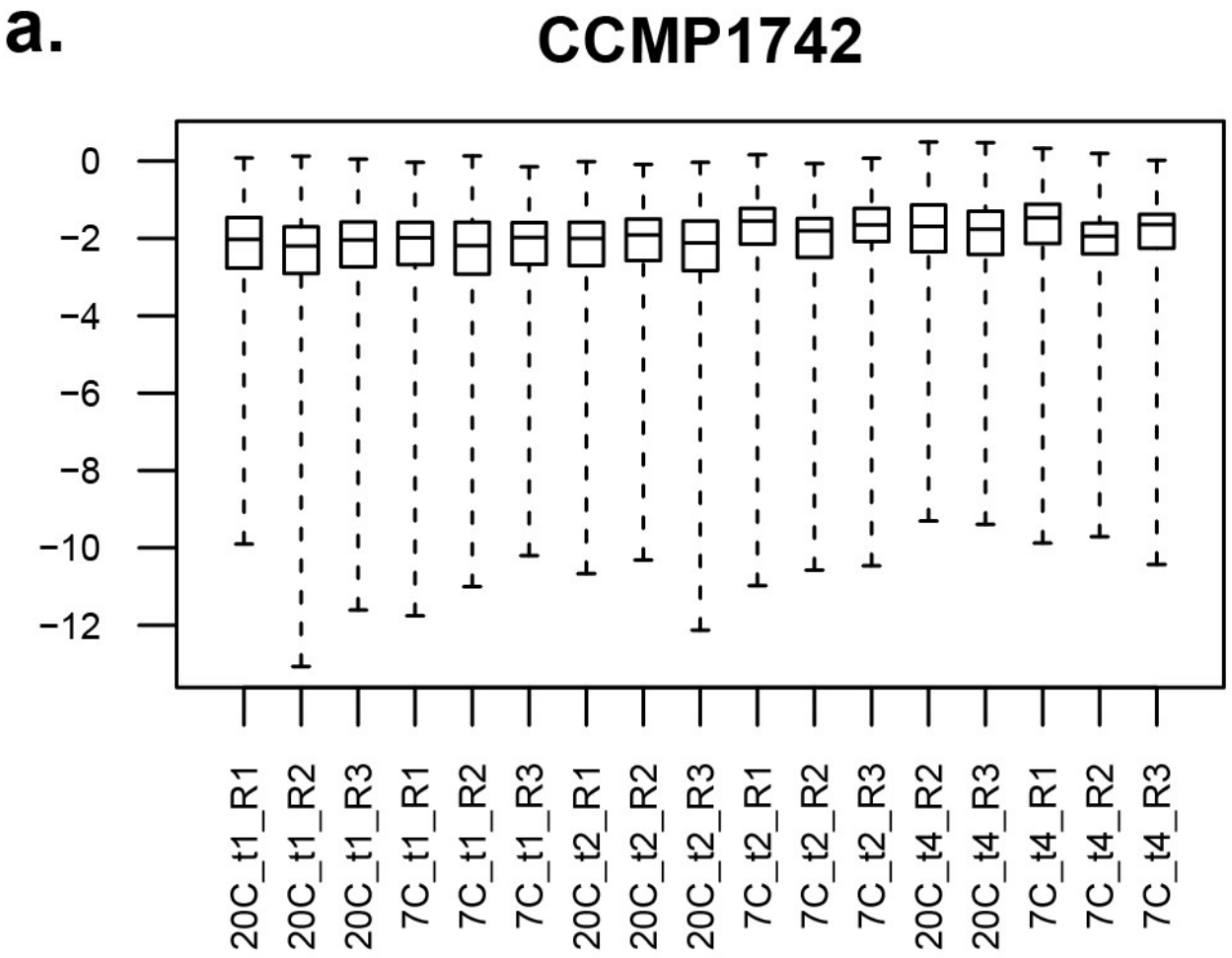

b.

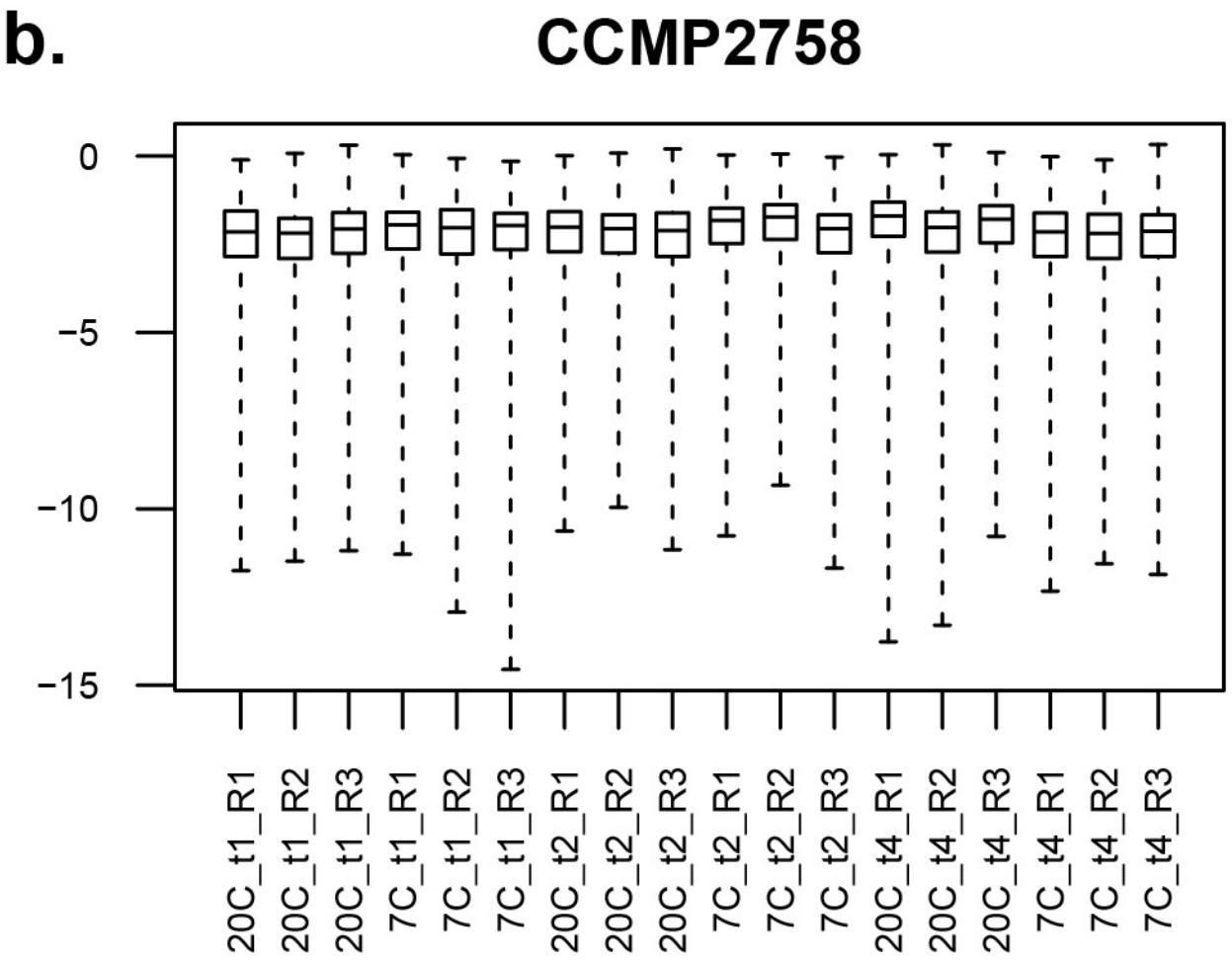

Figure S32. Cook's distance of transcriptome profiles used to identify outliers. Rows within a sample that have an extreme count outlier will have the p-value and adjusted p-value set to NA, and can be detected using Cook's distance. **(a)** CCMP1742 (excluding low-coverage sample), and **(b)** CCMP2758 (all samples).

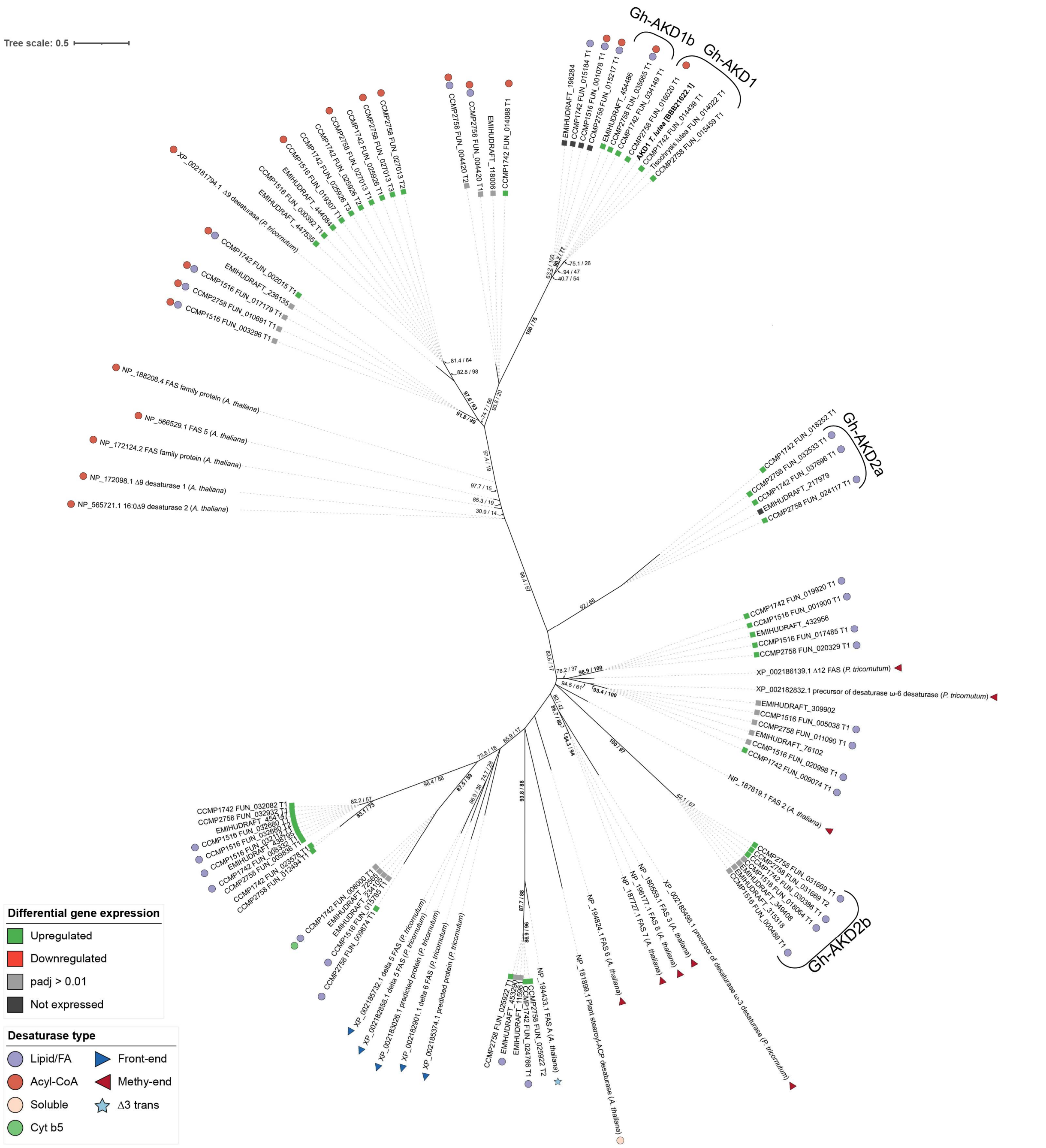

Figure S33. Unrooted phylogenetic protein three (same tree as in Figure 9) showing Tisochrysis lutea AKD1 (bold), a subset of the already characterized or annotated desaturases from *Phaeodactylum tricornutum* and *Arabidopsis thaliana* used in Supplementary Figure 1 of ref [33], the *G. huxleyi* CCMP1742 and CCMP2758 upregulated genes annotated as desaturases, and their orthologues in CCMP1516, CCMP1742 and CCMP2758 (same as Figure 9). Coloured squares indicate the differential expression of each gene. Note that differential gene expression annotation is shown on a per gene basis, therefore different protein isoforms originating from the same gene but noted differently by their "T" identifier will be represented as having the same differential gene expression and thus will be shown as a square of the same colour. Coloured circles, rectangles and stars indicate the annotated function of each desaturase. The tree was generated using the substitution model PMB+R3 as selected by Model Finder based on the Bayesian Information Criterion in IQ-tree. Values on branches indicate the standard non-parametric bootstrap supports (left side of the slash) and the SH-aLRT (right side of the slash), only values > 10 are shown. Scale bar represents the mean number of substitutions per site.

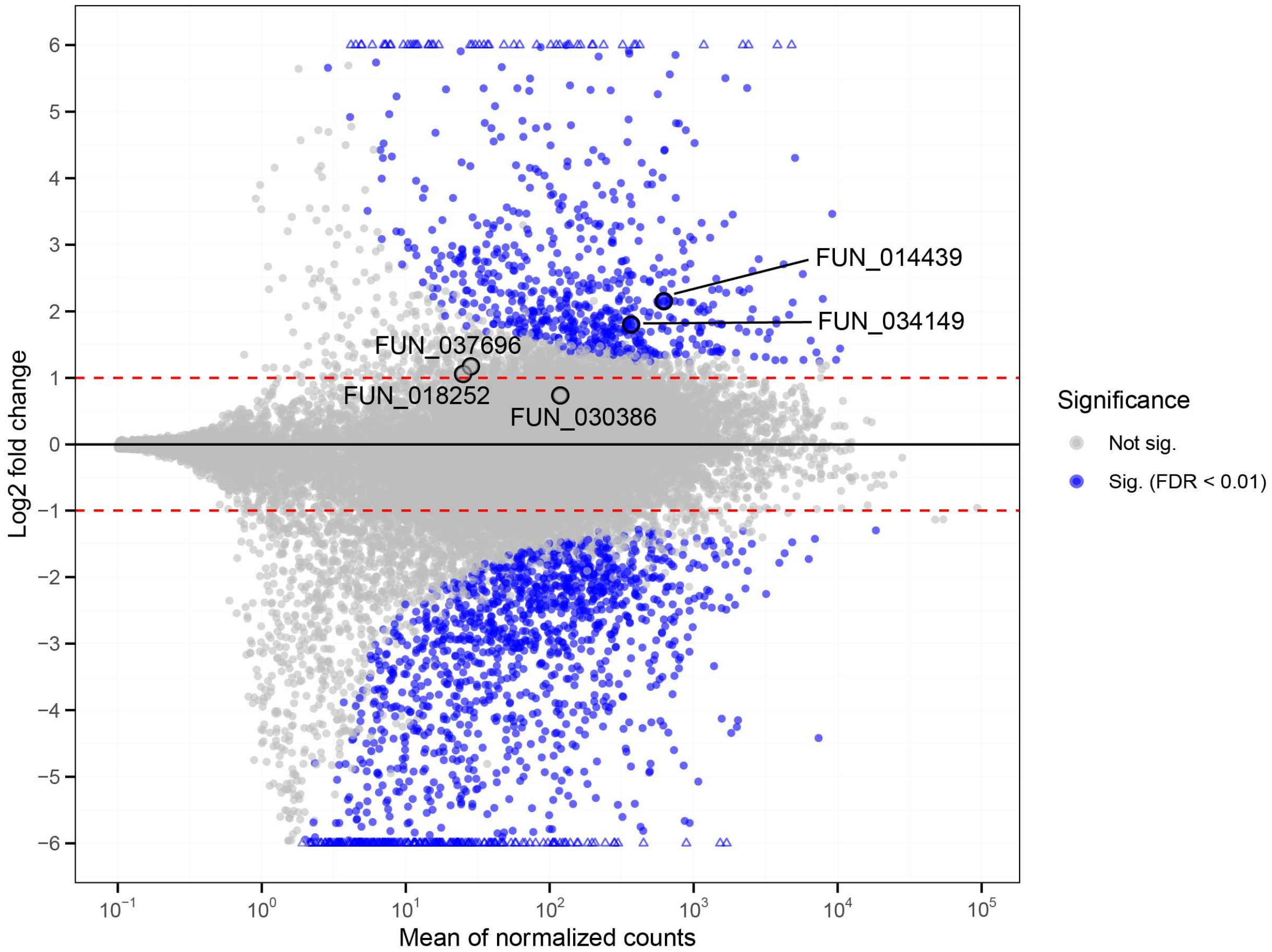

Figure S34. CCMP1742 MA-plot of differential gene expression across all time points comparing cold-adapted (7°C) and control (20°C) cultures. Y axis indicates the $\log_2$ fold changes (LFC) over the mean of normalized counts (x axis). Genes with an adjusted p-value ≤ 0.01 and a shrunken LFC of < -1 or >1 generated with apeglm [70] are indicated in blue. which fall out of the window are plotted as open triangles. Gene labels correspond to genes present in clusters Gh-AKD1 and Gh-AKD2 in Figure 9 and Figure S33. Plot generated using ggplot2 [69].

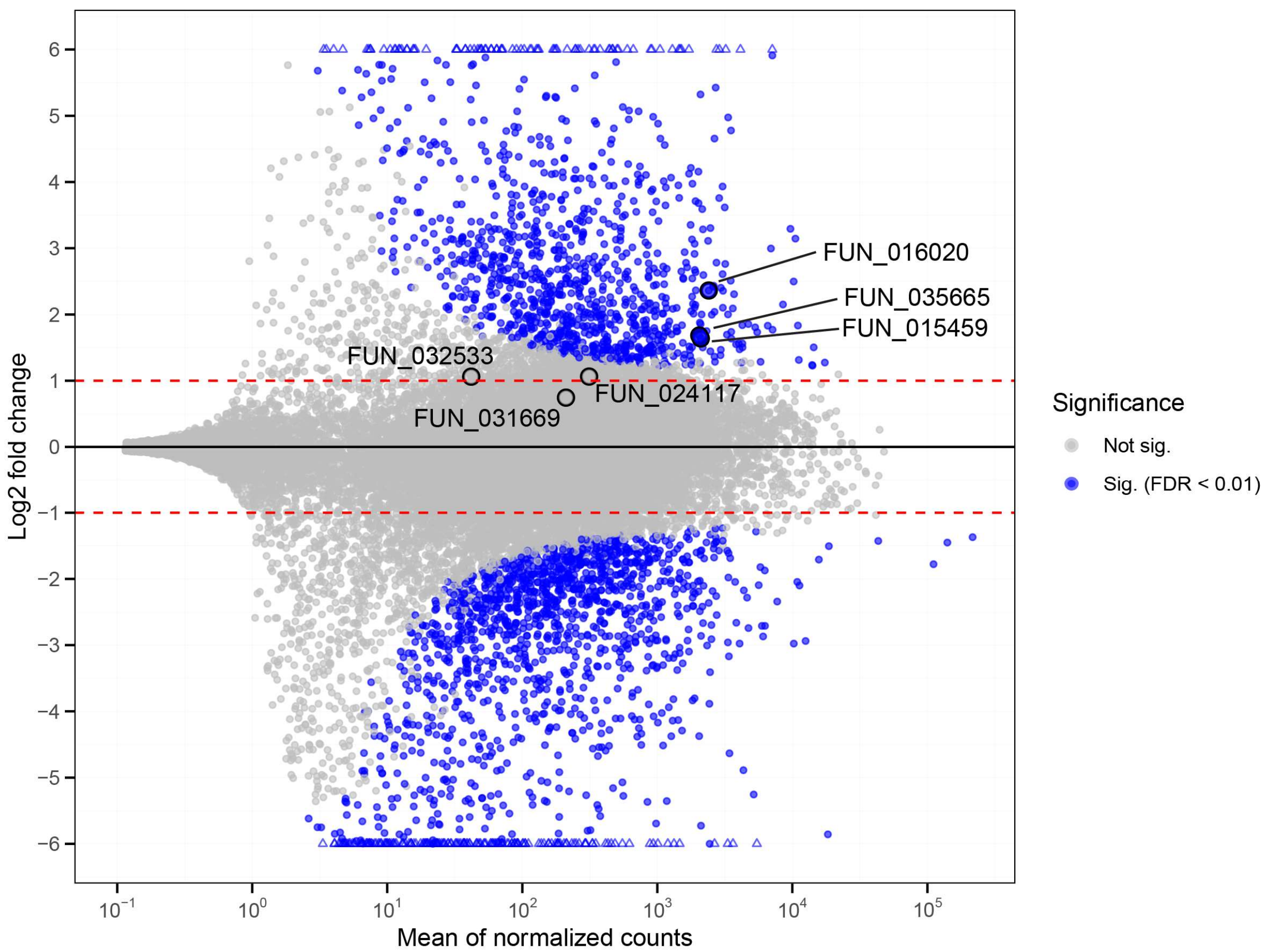

Figure S35. CCMP2758 MA-plot of differential gene expression across all time points comparing cold-adapted (7°C) and control (20°C) cultures. Y axis indicates the log_2 fold changes (LFC) over the mean of normalized counts (x axis). Genes with an p-value ≤0.01 and a shrunken LFC of < -1 or >1 generated with apeglm [70] are indicated in blue. Points which fall out of the window are plotted as open triangles. Gene labels correspond to genes present in clusters Gh-AKD1 and Gh-AKD2 in Figure 9 and Figure S33. Plot generated using ggplot2 [69].

Supplementary Data Captions

Data S1–S7 (captions below) are available from Zenodo at https://doi.org/10.5281/zenodo.15844033

Data S1. Gas Chromatography (GC) and GC-Mass Spectrometry (GC-MS) lipid data files. This folder contains raw data for all lipid analyses. The files are organized into subfolders based on the figure they support. The dataset includes the raw GC files used for quantification and the GC-MS files used for compound identification, including the specific chromatograms that generated the representative peaks shown in Figure 2. Where applicable, the subfolders also contain a summary table with the quantitative results extracted from the chromatograms and a sample metadata file that lists the experimental conditions (e.g., temperature, time point) and links them to the corresponding data file.

Data S2. *De novo* genome assemblies of *G. huxleyi* strains CCMP1742 and CCMP2758 generated during the evaluation of various assembly tools shown in Figure S2. These include long read assemblers (CANU v1.8, Flye v2.8.1,shasta v0.5.1, and wtdbg2 v2.3 with the wtpoa-cns v2.3 consenser), the short read assembler SPAdes v3.14.1, and hybrid assemblers that use both short and long reads (Wengan v0.2—using either DiscovarDeNovo [WenganD] or Minia3 [WenganM] as the short-read assembler—hybridSPAdes v3.14.1, biosyntheticSPAdes v3.14.1, and HASLR v0.8a1), and a metagenomic assembler, OPERA-MS v 0.8.2, in hybrid mode (using MegaHIT as short read assembler). A metagenomic assembler, OPERA-MS v0.8.2 run in hybrid mode with MegaHIT, was also evaluated. Furthermore, two scaffolders, WenganD and OPERA-LG v2.0.5, were applied to two selected assemblies (hybridSPAdes and biosyntheticSPAdes).

Data S3. *De novo* genome and metagenome assemblies of *G. huxleyi* strains CCMP1742 and CCMP2758 generated at different stages of the assembly process outlined in Figure 3. The included assemblies correspond to specific steps: (1) the initial hybrid metagenomic assemblies generated using metaSPAdes (v 3.14.1) and OPERA-MS (with SPAdes as the short-read assembler); (3) the hybrid assemblies created with SPAdes and biosyntheticSPAdes after a read-filtering step, where reads were mapped to the metagenomic assembly using Minimap2 and those mapping to contigs identified as likely contamination by CAT classification, coverage, and GC content were removed; (4) the scaffolded assemblies generated using OPERA-LG; and (7) the final assemblies after the identification and removal of remaining non-eukaryotic scaffolds.

Data S4. Funannotate genome annotation output files. This folder contains the "annotate_results" directories generated by Funannotate for the CCMP1742 and CCMP2758 assemblies. These assemblies were produced using metaSPAdes as a hybrid metagenomic assembler and biosyntheticSPAdes as a hybrid genome assembler, from which reads and contigs identified as likely contamination have been removed as described in Figure 3.

Supplementary Data S5. Cell count data for cold-shock and cold-adaptation experiments in *G. huxleyi* strains CCMP1742 and CCMP2758. This folder contains three files: two with data from the cold-shock experiments (one for each strain, CCMP1742 and CCMP2758) and one with data from the temperature acclimation experiment. The cold-shock datasets provide a summary of cell counts and viability measurements. For each sample, cell abundance was first measured on an unstained aliquot based on chlorophyll red autofluorescence (PerCP) versus forward scatter (FSC). A separate aliquot was then stained with SYTOX Green nucleic acid stain to determine cell viability, with a formalin-killed control used to define the gating strategy for dead cells. The data includes results from both untreated samples ("FCM" tab) and live/dead tests ("FCM_Live_dead" tab). Technical replicates for these experiments are included where applicable. The temperature acclimation dataset describes the cellular abundance data for strains grown at 20°C and 7°C. It reports raw counts for two distinct cell clusters ("Up" and "Down"), identified by their chlorophyll red autofluorescence (PerCP) versus forward scatter (FSC), and the total cell sum of both clusters for each sample. Data are presented as individual technical replicates ("raw" tabs), the average and standard deviation of those replicates per flask ("per_flask" tabs), and the average and standard deviation for each time point across three biological replicates (except t=0, n=1).

Data S6. Source data and statistical analysis for Figures 5, 6, and 7, and Figures S10, S15, S16, S23, S25 and S**31**. This file contains the underlying dataset and the results of the statistical analyses used to generate the indicated figures. The dataset includes the underlying numerical values and categorical labels plotted in the figures, as well as the full outputs of statistical analyses—including test statistics, degrees of freedom, p-values, confidence intervals, and post-hoc comparisons where applicable.

Data S7. RNA-seq alignment and deduplication metrics. This file contains the output metrics from the STAR aligner and UMI-tools deduplication for RNA-seq data. It includes key alignment statistics—such as the number of input reads, uniquely mapped reads (%), and reads mapped to multiple loci (%)—for strains CCMP1742 and CCMP2758 before and after deduplication, and for strain CCMP1516 (non-deduplicated). The file also includes the deduplication results from running "umi_tools dedup" command for samples of CCMP1742 and CCMP2758 strains, reporting metrics such as number of input reads, final output counts and mean and maximum number of unique UMIs per position.

Data S8. Phylogeny of desaturases. This folder contains the file used to generate Figure 9 and Figure S33. The folder contains the alignment and trimmed alignment, IQ-TREE output files, and iTOL annotation files.

Supplementary References

1. De La Cerda GY, Landis JB, Eifler E, Hernandez AI, Li F, Zhang J, et al. Balancing read length and sequencing depth: Optimizing Nanopore long-read sequencing for monocots with an emphasis on the Liliales. Appl Plant Sci. 2023;11:e11524. https://doi.org/10.1002/aps3.11524.

2. Read BA, Kegel J, Klute MJ, Kuo A, Lefebvre SC, Maumus F, et al. Pan genome of the phytoplankton *Emiliania* underpins its global distribution. Nature. 2013;499:209–13. https://doi.org/10.1038/nature12221.

3. Magoc T, Pabinger S, Canzar S, Liu X, Su Q, Puiu D, et al. GAGE-B: an evaluation of genome assemblers for bacterial organisms. Bioinformatics. 2013;29:1718–25. https://doi.org/10.1093/bioinformatics/btt273.

4. Hanschen ER, Starkenburg SR. The state of algal genome quality and diversity. Algal Research. 2020;50:101968. https://doi.org/10.1016/j.algal.2020.101968.

5. Gurevich A, Saveliev V, Vyahhi N, Tesler G. QUAST: quality assessment tool for genome assemblies. Bioinformatics. 2013;29:1072–5. https://doi.org/10.1093/bioinformatics/btt086.

6. Koren S, Walenz BP, Berlin K, Miller JR, Bergman NH, Phillippy AM. Canu: scalable and accurate long-read assembly via adaptive k-mer weighting and repeat separation. Genome Res. 2017;27:722–36. https://doi.org/10.1101/gr.215087.116.

7. Kolmogorov M, Yuan J, Lin Y, Pevzner PA. Assembly of long, error-prone reads using repeat graphs. Nat Biotechnol. 2019;37:540–6. https://doi.org/10.1038/s41587-019-0072-8.

8. Shafin K, Pesout T, Lorig-Roach R, Haukness M, Olsen HE, Bosworth C, et al. Nanopore sequencing and the Shasta toolkit enable efficient de novo assembly of eleven human genomes. Nat Biotechnol. 2020;38:1044–53. https://doi.org/10.1038/s41587-020-0503-6.

9. Ruan J, Li H. Fast and accurate long-read assembly with wtdbg2. Nat Methods. 2020;17:155–8. https://doi.org/10.1038/s41592-019-0669-3.

10. Hanschen ER, Hovde BT, Starkenburg SR. An evaluation of methodology to determine algal genome completeness. Algal Research. 2020;51:102019. https://doi.org/10.1016/j.algal.2020.102019.

11. Rontani J-F, Prahl FG, Volkman JK. Re-Examination of the Double Bond Positions in Alkenones and Derivatives: Biosynthetic Implications. Journal of Phycology. 2006;42:800–13. https://doi.org/10.1111/j.1529-8817.2006.00251.x.

12. Zheng Y, Dillon JT, Zhang Y, Huang Y. Discovery of alkenones with variable methylene-interrupted double bonds: implications for the biosynthetic pathway. J Phycol. 2016;52:1037–50. https://doi.org/10.1111/jpy.12461.

13. Rontani J-F, Marchand D, Volkman JK. $NaBH_4$ reduction of alkenones to the corresponding alkenols: a useful tool for their characterisation in natural samples. Organic Geochemistry. 2001;32:1329–41. https://doi.org/10.1016/S0146-6380(01)00091-2.

14. Chen H, Du L. Iterative Polyketide Biosynthesis by Modular Polyketide Synthases in Bacteria. Appl Microbiol Biotechnol. 2016;100:541–57. https://doi.org/10.1007/s00253-015-7093-0.

15. Moss SJ, Martin CJ, Wilkinson B. Loss of co-linearity by modular polyketide synthases: a mechanism for the evolution of chemical diversity. Nat Prod Rep. 2004;21:575–93. https://doi.org/10.1039/B315020H.

16. El-Sayed AK, Hothersall J, Cooper SM, Stephens E, Simpson TJ, Thomas CM. Characterization of the mupirocin biosynthesis gene cluster from *Pseudomonas fluorescens* NCIMB 10586. Chem Biol. 2003;10:419–30. https://doi.org/10.1016/s1074-5521(03)00091-7.

17. Prahl FG, Rontani J-F, Volkman JK, Sparrow MA, Royer IM. Unusual C_{35} and C_{36} alkenones in a paleoceanographic benchmark strain of *Emiliania huxleyi*. Geochimica et Cosmochimica Acta. 2006;70:2856–67. https://doi.org/10.1016/j.gca.2006.03.009.

18. Xu L, Reddy CM, Farrington JW, Frysinger GS, Gaines RB, Johnson CG, et al. Identification of a novel alkenone in Black Sea sediments. Organic Geochemistry. 2001;32:633–45.

19. Rontani J-F, Prahl FG, Volkman JK. Characterization of unusual alkenones and alkyl alkenoates by electron ionization gas chromatography/mass spectrometry. Rapid Commun Mass Spectrom. 2006;20:583–8. https://doi.org/10.1002/rcm.2346.

20. van Soelen EE, Lammers JM, Eglinton T. I, Sinninghe Damsté JS, Reichart GJ. Organic Geochemistry Unusual C35 to C38 alkenones in mid-Holocene sediments from a restricted estuary (Charlotte Harbor, Florida). Organic Geochemistry. 2014;70:20–8. https://doi.org/10.1016/j.orggeochem.2014.01.021.

21. Lopez JF, de Oteyza TG, Teixidor P, Grimalt JO. Long chain alkenones in hypersaline and marine coastal microbial mats. Organic Geochemistry. 2005;36:861–72. https://doi.org/10.1016/j.orggeochem.2005.01.008.

22. Moore BS, Hertweck C. Biosynthesis and attachment of novel bacterial polyketide synthase starter units. Natural Product Reports. 2002;19:70–99. https://doi.org/10.1039/B003939J.

23. Song L, Jenner M, Masschelein J, Jones C, Bull MJ, Harris SR, et al. Discovery and Biosynthesis of Gladiolin: A Burkholderia gladioli Antibiotic with Promising Activity against Mycobacterium tuberculosis. J Am Chem Soc. 2017;139:7974–81. https://doi.org/10.1021/jacs.7b03382.

24. Ray L, Moore BS. Recent advances in the biosynthesis of unusual polyketide synthase substrates. Nat Prod Rep. 2016;33:150–61. https://doi.org/10.1039/C5NP00112A.

25. Shelest E, Heimerl N, Fichtner M, Sasso S. Multimodular type I polyketide synthases in algae evolve by module duplications and displacement of AT domains in trans. BMC Genomics. 2015;16:1015. https://doi.org/10.1186/s12864-015-2222-9.

26. Kwan DH, Schulz F. The Stereochemistry of Complex Polyketide Biosynthesis by Modular Polyketide Synthases. Molecules. 2011;16:6092–115. https://doi.org/10.3390/molecules16076092.

27. Yin Z, Dickschat JS. *Cis* double bond formation in polyketide biosynthesis. Nat Prod Rep. 2021;38:1445–68. https://doi.org/10.1039/D0NP00091D.

28. Staunton J, Weissman KJ. Polyketide biosynthesis: a millennium review. Nat Prod Rep. 2001;18:380–416. https://doi.org/10.1039/a909079g.

29. Klassen JL, Currie CR. Gene fragmentation in bacterial draft genomes: extent, consequences and mitigation. BMC Genomics. 2012;13:14. https://doi.org/10.1186/1471-2164-13-14.

30. Meleshko D, Mohimani H, Traccana V, Hajirasouliha I, Medema MH, Korobeynikov A, et al. BiosyntheticSPAdes: Reconstructing Biosynthetic Gene Clusters From Assembly Graphs. Genome Res. 2019;:gr.243477.118. https://doi.org/10.1101/gr.243477.118.

31. Dillon JT, Longo WM, Zhang Y, Torozo R, Huang Y. Identification of double-bond positions in isomeric alkenones from a lacustrine haptophyte. Rapid Commun Mass Spectrom. 2016;30:112–8. https://doi.org/10.1002/rcm.7414.

32. Sperling P, Ternes P, Zank TK, Heinz E. The evolution of desaturases. Prostaglandins, Leukotrienes and Essential Fatty Acids. 2003;68:73–95. https://doi.org/10.1016/S0952-3278(02)00258-2.

33. Endo H, Hanawa Y, Araie H, Suzuki I, Shiraiwa Y. Overexpression of *Tisochrysis lutea* Akd1 identifies a key cold-induced alkenone desaturase enzyme. Scientific Reports. 2018;8:11230. https://doi.org/10.1038/s41598-018-29482-8.

34. Altschul SF, Wootton JC, Gertz EM, Agarwala R, Morgulis A, Schäffer AA, et al. Protein database searches using compositionally adjusted substitution matrices. FEBS J. 2005;272:5101–9. https://doi.org/10.1111/j.1742-4658.2005.04945.x.

35. Altschul SF, Madden TL, Schäffer AA, Zhang J, Zhang Z, Miller W, et al. Gapped BLAST and PSI-BLAST: a new generation of protein database search programs. Nucleic Acids Res. 1997;25:3389–402. https://doi.org/10.1093/nar/25.17.3389.

36. Emms DM, Kelly S. OrthoFinder: phylogenetic orthology inference for comparative genomics. Genome Biology. 2019;20:238. https://doi.org/10.1186/s13059-019-1832-y.

37. Sun J, Lu F, Luo Y, Bie L, Xu L, Wang Y. OrthoVenn3: an integrated platform for exploring and visualizing orthologous data across genomes. Nucleic Acids Research. 2023;51:W397–403. https://doi.org/10.1093/nar/gkad313.

38. Cook RD. Detection of influential observation in linear regression. Technometrics. 1977;19:15–8.

39. Prahl FG, Muehlhausen LA, Zahnle DL. Further evaluation of long-chain alkenones as indicators of paleoceanographic conditions. Geochimica et Cosmochimica Acta. 1988;52:2303–10. https://doi.org/10.1016/0016-7037(88)90132-9.

40. Zheng Y, Tarozo R, Huang Y. Optimizing chromatographic resolution for simultaneous quantification of long chain alkenones, alkenoates and their double bond positional isomers. Organic Geochemistry. 2017;111:136–43. https://doi.org/10.1016/j.orggeochem.2017.06.013.

41. Longo WM, Theroux S, Giblin AE, Zheng Y, Dillon JT, Huang Y. Temperature calibration and phylogenetically distinct distributions for freshwater alkenones: Evidence from northern Alaskan lakes. Geochimica et Cosmochimica Acta. 2016;180:177–96. https://doi.org/10.1016/j.gca.2016.02.019.

42. O'Neil GW, Keller A, Balila J, Golden S, Sipila N, Stone B, et al. Monitoring Changes to Alkenone Biosynthesis in Commercial *Tisochrysis lutea* Microalgae. ACS Omega. 2024;9:16374–83. https://doi.org/10.1021/acsomega.4c00087.

43. Russell NJ. Mechanisms of thermal adaptation in bacteria: blueprints for survival. Trends in Biochemical Sciences. 1984;9:108–12. https://doi.org/10.1016/0968-0004(84)90106-3.

44. Patterson GW. Effect of culture temperature on fatty acid composition of *Chlorella sorokiniana*. Lipids. 1970;5:597–600. https://doi.org/10.1007/BF02531336.

45. Carrier G, Baroukh C, Rouxel C, Duboscq-Bidot L, Schreiber N, Bougaran G. Draft genomes and phenotypic characterization of *Tisochrysis lutea* strains. Toward the production of domesticated strains with high added value. Algal Research. 2018;29:1–11. https://doi.org/10.1016/j.algal.2017.10.017.

46. Shi Q, Araie H, Bakku RK, Fukao Y, Rakwal R, Suzuki I, et al. Proteomic analysis of lipid body from the alkenone-producing marine haptophyte alga *Tisochrysis lutea*. Proteomics. 2015;15:4145–58. https://doi.org/10.1002/pmic.201500010.

47. Yoneda K, Kobayashi C, Araie H, Morita R, Harada R, Shigeta Y, et al. Characterization of Delta-7 Alkenone Desaturase in Haptophyte *Gephyrocapsa huxleyi* Through Heterologous Expression in *Tisochrysis lutea*. Mar Biotechnol (NY). 2025;27:44. https://doi.org/10.1007/s10126-025-10427-y.

48. Guillard RRL, Hargraves PE. *Stichochrysis immobilis* is a diatom, not a chrysophyte. Phycologia. 1993;32:234–6. https://doi.org/10.2216/i0031-8884-32-3-234.1.

49. Grabherr MG, Haas BJ, Yassour M, Levin JZ, Thompson DA, Amit I, et al. Full-length transcriptome assembly from RNA-Seq data without a reference genome. Nat Biotechnol. 2011;29:644–52. https://doi.org/10.1038/nbt.1883.

50. Lomsadze A, Ter-Hovhannisyan V, Chernoff YO, Borodovsky M. Gene identification in novel eukaryotic genomes by self-training algorithm. Nucleic Acids Research. 2005;33:6494–506. https://doi.org/10.1093/nar/gki937.

51. Chan PP, Lin BY, Mak AJ, Lowe TM. tRNAscan-SE 2.0: improved detection and functional classification of transfer RNA genes. Nucleic Acids Research. 2021;49:9077–96. https://doi.org/10.1093/nar/gkab688.

52. Kautsar SA, Suarez Duran HG, Blin K, Osbourn A, Medema MH. plantiSMASH: automated identification, annotation and expression analysis of plant biosynthetic gene clusters. Nucleic Acids Research. 2017;45:W55–63. https://doi.org/10.1093/nar/gkx305.

53. Käll L, Krogh A, Sonnhammer ELL. A Combined Transmembrane Topology and Signal Peptide Prediction Method. Journal of Molecular Biology. 2004;338:1027–36. https://doi.org/10.1016/j.jmb.2004.03.016.

54. Cantalapiedra CP, Hernández-Plaza A, Letunic I, Bork P, Huerta-Cepas J. eggNOG-mapper v2: Functional Annotation, Orthology Assignments, and Domain Prediction at the Metagenomic Scale. Molecular Biology and Evolution. 2021;38:5825–9. https://doi.org/10.1093/molbev/msab293.

55. Huerta-Cepas J, Szklarczyk D, Heller D, Hernández-Plaza A, Forslund SK, Cook H, et al. eggNOG 5.0: a hierarchical, functionally and phylogenetically annotated orthology resource based on 5090 organisms and 2502 viruses. Nucleic Acids Research. 2019;47:309–14. https://doi.org/10.1093/nar/gky1085.

56. Almagro Armenteros JJ, Tsirigos KD, Sønderby CK, Petersen TN, Winther O, Brunak S, et al. SignalP 5.0 improves signal peptide predictions using deep neural networks. Nat Biotechnol. 2019;37:420–3. https://doi.org/10.1038/s41587-019-0036-z.

57. Jones P, Binns D, Chang H-Y, Fraser M, Li W, McAnulla C, et al. InterProScan 5: genome-scale protein function classification. Bioinformatics. 2014;30:1236–40. https://doi.org/10.1093/bioinformatics/btu031.

58. McKinney W. Data Structures for Statistical Computing in Python. scipy. 2010. https://doi.org/10.25080/Majora-92bf1922-00a.

59. Waskom ML. seaborn: statistical data visualization. Journal of Open Source Software. 2021;6:3021. https://doi.org/10.21105/joss.03021.

60. Harris CR, Millman KJ, van der Walt SJ, Gommers R, Virtanen P, Cournapeau D, et al. Array programming with NumPy. Nature. 2020;585:357–62. https://doi.org/10.1038/s41586-020-2649-2.

61. Hunter JD. Matplotlib: A 2D Graphics Environment. Computing in Science & Engineering. 2007;9:90–5. https://doi.org/10.1109/MCSE.2007.55.

62. Katoh K, Standley DM. MAFFT Multiple Sequence Alignment Software Version 7: Improvements in Performance and Usability. Molecular Biology and Evolution. 2013;30:772–80. https://doi.org/10.1093/molbev/mst010.

63. Capella-Gutiérrez S, Silla-Martínez JM, Gabaldón T. trimAl: a tool for automated alignment trimming in large-scale phylogenetic analyses. Bioinformatics. 2009;25:1972–3. https://doi.org/10.1093/bioinformatics/btp348.

64. Kalyaanamoorthy S, Minh BQ, Wong TKF, von Haeseler A, Jermiin LS. ModelFinder: fast model selection for accurate phylogenetic estimates. Nat Methods. 2017;14:587–9. https://doi.org/10.1038/nmeth.4285.

65. Guindon S, Dufayard J-F, Lefort V, Anisimova M, Hordijk W, Gascuel O. New Algorithms and Methods to Estimate Maximum-Likelihood Phylogenies: Assessing the Performance of PhyML 3.0. Systematic Biology. 2010;59:307–21. https://doi.org/10.1093/sysbio/syq010.

66. Letunic I, Bork P. Interactive Tree Of Life (iTOL) v5: an online tool for phylogenetic tree display and annotation. Nucleic Acids Research. 2021;49:W293–6. https://doi.org/10.1093/nar/gkab301.

67. De Leeuw JW, V.D. Meer FW, Rijpstra WIC, Schenck PA. On the occurrence and structural identification of long chain unsaturated ketones and hydrocarbons in sediments. Physics and Chemistry of the Earth. 1980;12:211–7. https://doi.org/10.1016/0079-1946(79)90105-8.

68. López JF, Grimalt JO. Reassessment of the structural composition of the alkenone distributions in natural environments using an improved method for double bond location based on GC-MS analysis of cyclopropylimines. J Am Soc Mass Spectrom. 2006;17:710–20. https://doi.org/10.1016/j.jasms.2006.01.015.

69. Wickham H. ggplot2: Elegant Graphics for Data Analysis. Springer-Verlag New York; 2016.

70. Zhu A, Ibrahim JG, Love MI. Heavy-tailed prior distributions for sequence count data: removing the noise and preserving large differences. Bioinformatics. 2019;35:2084–92. https://doi.org/10.1093/bioinformatics/bty895.
